# Global ethnic disparities in cerebral small vessel disease imaging markers and vascular risk factors: a systematic review and meta‐analysis

**DOI:** 10.1002/alz.70976

**Published:** 2026-02-09

**Authors:** Nikita K. Husein, Keshuo Lin, David K. E. Chan, Jiyang Jiang, John D. Crawford, Perminder S. Sachdev, Wei Wen

**Affiliations:** ^1^ Centre for Healthy Brain Ageing (CHeBA) Discipline of Psychiatry and Mental Health, School of Clinical Medicine University of New South Wales Kensington New South Wales Australia; ^2^ Stats Central, Statistical Consulting Unit, School of Mathematics and Statistics University of New South Wales Kensington New South Wales Australia; ^3^ Research Imaging NSW UNSW Sydney Kensington New South Wales Australia; ^4^ Neuropsychiatric Institute Prince of Wales Hospital Randwick New South Wales Australia

**Keywords:** cardiometabolic risk factor, cerebral small vessel disease, ethnic groups, magnetic resonance imaging, meta‐analysis, neuroimaging, vascular risk factors

## Abstract

Ethnic disparities in cerebral small vessel disease (CSVD) have been reported, but no systematic synthesis has compared markers and vascular risk factors across populations. We conducted a PROSPERO‐registered systematic review and meta‐analysis (CRD42024518105) including over two million community‐dwelling adults. A two‐tier ethnicity framework was applied. Tier 1 used broad US Office of Management and Budget‐aligned categories to maximize comparability, showing that Asians with higher cerebral microbleeds, Whites with greater metabolic risks, Blacks with higher hypertension, and Hispanics with elevated diabetes. Tier 2 enabled finer intra‐Asian subgrouping, revealing Chinese cohorts with greater white matter hyperintensity (WMH) and hemorrhagic burden, Japanese with a lacunar‐predominant profile but lower microbleeds, and Koreans with blood pressure–linked WMH. Ethnicity also moderated key risk–lesion associations, including stronger effects of diabetes on WMH and lacunes in Chinese and of systolic blood pressure on WMHs in Koreans. These findings highlight that CSVD phenotypes differ across populations, reflecting complex interactions between vascular, genetic, and environmental factors.

**Highlights:**

- First meta‐analysis of CSVD markers stratified by global ethnic groups.
- Tier 1: Asians had higher CMBs and lacunes; Whites had higher metabolic risk.
- Tier 2: Chinese showed higher WMHs and CMBs; Japanese had more lacunes, fewer CMBs.
- Risk–CSVD associations varied by ethnicity, including diabetes and blood pressure.
- Results suggest need for ethnicity‐informed prevention and harmonised reporting.

## 1 INTRODUCTION

Cerebral small vessel disease (CSVD) is a common neurological condition affecting the brain's smallest blood vessels. Previously considered clinically silent, CSVD is now increasingly recognized for its relationship to increased risk for stroke, cognitive impairment, gait abnormality, and neuropsychiatric symptoms.^1^ Several markers of CSVD can be detected using advanced magnetic resonance imaging (MRI)‐based imaging techniques.^2^, ^3^ The updated STandards for ReportIng Vascular changes on nEuroimaging (STRIVE) criteria highlight key CSVD neuroimaging markers, such as white matter hyperintensities (WMHs), lacunes, cerebral microbleeds, microinfarcts, and enlarged perivascular spaces.^4^, ^5^

Vascular risk factors are central to CSVD pathogenesis. Hypertension is the strongest predictor, conferring up to a threefold higher risk of lesions, while diabetes, smoking, hyperlipidemia, and obesity also contribute significantly.^6^ Aging further accelerates risk; population studies show CSVD burden doubles with each additional decade of life.^7^ These findings underscore the importance of midlife vascular risk factor control, particularly blood pressure.

However, much of this evidence comes from predominantly White cohorts, such as Framingham and Rotterdam.^8^ Growing data highlight important ethnic disparities. Black and Hispanic groups in the United States have higher WMH volumes and lacunar prevalence than Whites, only partly explained by hypertension and diabetes,^8^, ^9^ and are disproportionately affected by adverse vascular risk exposures.^10^, ^11^ Asian populations show distinct patterns: East Asians have nearly double the incidence of intracerebral hemorrhage compared to Europeans^12^ and a higher frequency of lacunar stroke.^13^ Community studies report lacunes in over 30% of middle‐aged East Asians versus under 10% in some European cohorts of similar age.^7^ Recent global data further suggest CSVD burden is greater in low‐ and middle‐income countries.^9^

In summary, CSVD is a clinically important and globally prevalent disease with well‐defined vascular risk factors. Yet its burden and risk–lesion relationships vary across ethnic groups, suggesting differences in exposure, vulnerability, and context.^10^, ^12^ Expanding CSVD research beyond White populations is essential to clarify these disparities and inform tailored prevention strategies. This meta‐analysis addresses this gap by synthesising CSVD neuroimaging markers and vascular risk factors across multiple ethnicities.

### 1.1 Scope

This study is the first systematic review and meta‐analysis to compare ethnic differences in CSVD markers – including WMHs, lacunes, cerebral microbleeds (CMBs), and microinfarcts and related vascular risk factors in community‐dwelling adults. We applied a two‐tier ethnicity framework. Tier 1 used broad US Office of Management and Budget (OMB)‐aligned categories (Asian, Black or African American, White, and Hispanic/Latino) to maximize comparability and power. Studies with sufficient numbers were meta‐analyzed, while underpowered groups were retained and narratively reported, ensuring full use of the dataset. Tier 2 analyses were restricted to Asian populations, where ≥3 studies per subgroup enabled robust comparisons between Chinese (Mainland, Hong Kong, and Taiwan), Japanese, and South Korean groups. Other ethnicities lacked adequate representation and were therefore analyzed only at Tier 1. Our review evaluates study‐level ethnic differences in CSVD burden and risk factor prevalence, with interaction analyses testing whether ethnicity moderated these associations. By combining broad Tier 1 contrasts, narrative synthesis of smaller groups, and finer Tier 2 Asian subgrouping, the study provides both population‐wide insights and clinically relevant intra‐Asian comparisons, while remaining transparent about the limits of available evidence.

### 1.2 Target audience of review

The target audience for this review included clinicians, researchers, and public health professionals interested in CSVD, ethnic health disparities, and vascular risk stratification in aging populations.

## 2 METHODS

This systematic review was conducted in accordance with the *Cochrane Handbook for Systematic Reviews of Interventions* (version 6.4), with study eligibility and question formulation guided by the Population, Intervention/exposure, Comparison, Outcome, and Study (PICOS) design framework. The review protocol was registered in PROSPERO (CRD42024518105), and the manuscript was prepared following the Preferred Reporting Items for Systematic Reviews and Meta‐Analyses (PRISMA) 2020 guideline (Table S1). The Grading of Recommendation, Assessment, Development and Evaluation (GRADE) approach was used to assess the certainty of evidence for key outcomes and to structure judgments about the quality and strength of findings.

### 2.1 Research question

The PICOS design framework was used. The population included community‐dwelling adults from population‐based studies, excluding disease‐enriched or clinical samples. The exposure included cardiometabolic and sociodemographic risk factors, such as diabetes, hypertension, body mass index (BMI), smoking, alcohol use, and socioeconomic indicators. This review did not include a formal comparison group. Outcomes included MRI‐defined CSVD neuroimaging markers, including WMHs, lacunes, cerebral microbleeds, and microinfarcts. Only observational population‐based studies were included.

### 2.2 Search strategy

A comprehensive search was built with the aid of a medical librarian and performed in PubMed, Embase (OVID), Web of Science, and PsycINFO (OVID) from database inception to August 2025, with no language restrictions, including peer‐reviewed articles and preprints. Search filters were applied to exclude randomized controlled trials, editorials, conference abstracts, and review articles. Full search terms for all the databases are provided in Section S1 and Table S2.

**FIGURE 1:**
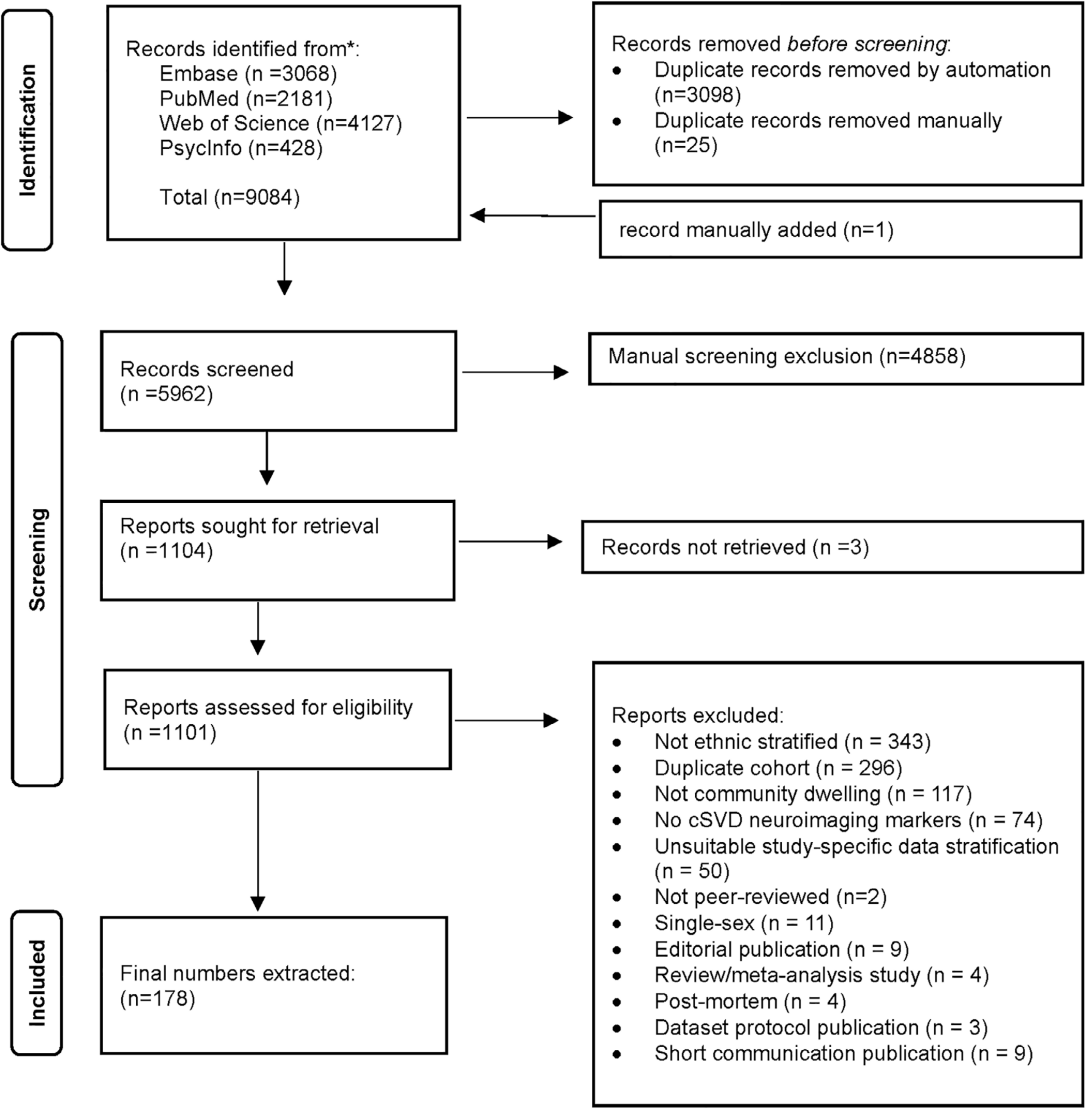
Study selection flowchart. Preferred Reporting Items for Systematic Reviews and Meta‐Analyses (PRISMA)‐based flowchart depicting identification, screening, and eligibility process for included studies. A total of 9084 records were identified from four databases: Embase (*n* = 3068), PubMed (*n* = 2181), Web of Science (*n* = 4127), and PsycInfo (*n* = 428). After removal of 3098 automated and 25 manual duplicates, 5962 records were screened. Following title/abstract screening, 4858 records were excluded. Of 1104 full texts sought for retrieval, 1101 were assessed, with 923 excluded (e.g., not ethnicity‐stratified [*n* = 343], duplicate cohort [*n* = 296], not community‐dwelling [*n* = 117], no cerebral small vessel disease neuroimaging markers [*n* = 74]). Ultimately, 178 studies were included in the systematic review and meta‐analysis, with one record manually added.

A total of 9817 records were identified from searches across the aforementioned databases. After removing 4712 duplicates and adding one record manually, 5114 records were eligible for initial screening. Following title and abstract screening, 4164 records were excluded. Of the 949 studies retrieved for full‐text review, 790 were excluded for reasons illustrated in Figure 1. This left 159 studies eligible for inclusion in the systematic review and meta‐analysis.

### 2.3 Eligibility criteria

We included studies that examined CSVD neuroimaging markers in adults (≥18 years) from community or general populations. Eligible studies were required to use MRI‐based imaging, specifically T1‐weighted imaging, T2‐weighted imaging, Fluid‐Attenuated Inversion Recovery (FLAIR), susceptibility‐weighted imaging (SWI), diffusion tensor imaging (DTI), diffusion‐weighted imaging (DWI) to assess STRIVE criteria for CSVD lesions, including^5^ WMH volume and the Fazekas scale^14^ for CMBs, lacunes, microinfarcts, or enlarged perivascular spaces (ePVS), though due to lower ethnic diversity availability in ePVS, the measure was excluded in the analysis. Other studies with semi‐quantitative measures, measuring white matter lesions (WMLs) other than the Fazekas scale were not meta‐analyzed but is detailed in Table S3. Studies needed to report cardiovascular risk factors (hypertension, diabetes, hyperlipidemia, BMI, smoking, and alcohol use) and sociodemographic factors (education, socioeconomic status, and geographic location) and include ethnicity‐stratified data to allow meaningful comparisons across ethnic groups. Ethnicity was treated as a core exposure variable, and the search strategy incorporated controlled vocabulary and free‐text terms for major racial and ethnic categories across databases (e.g., MeSH and Emtree), including both broad descriptors (e.g., “ethnic group” and “racial background”) and population‐specific identifiers (e.g., “East Asian,” “African American,” “Hispanic or Latino,” and “White”) to ensure comprehensive retrieval of studies reporting ethnicity‐stratified data.

Studies were excluded if they (1) focused on clinical populations (e.g., stroke and dementia), (2) were case‐control studies, reviews, editorials, commentaries, case reports, or conference abstracts, (3) included participants receiving therapeutic interventions, (4) used non‐MRI techniques (e.g., CT), (5) did not provide ethnicity‐stratified CSVD measures or risk factors, or (6) failed to assess key CSVD neuroimaging markers or relevant cardiovascular/sociodemographic risk factors. Table S4 provides excluded references at full stage screening stage and reasoning.

### 2.4 Data extraction and risk of bias assessment

Two independent reviewers (Nikita K. Husein and Keshuo Lin) screened studies using Screenatron (title/abstracts)^15^ and Covidence (full‐texts),^16^ with disagreements resolved by a third reviewer (Wei Wen) or fourth reviewer (Jiyang Jiang) if needed. Data were extracted using a structured form in Covidence, capturing study characteristics, population demographics, imaging techniques, CSVD markers, and risk factors, with the data extraction form listed in Figure S1.

Risk of bias was assessed with the Risk of Bias Assessment Tool for Non‐Randomized Studies (RoBANS), evaluating selection bias, confounding, exposure measurement, blinding, incomplete data, and selective reporting. Disagreements were resolved through discussion (Nikita K. Husein, Wei Wen, and Jiyang Jiang). Furthermore, certainty of evidence was appraised using the GRADE approach, evaluating risk of bias, inconsistency, indirectness, imprecision, and publication bias for each outcome domain. Ratings were informed by the ROBANS‐E tool (Section S2 and Table S5), heterogeneity statistics, and visual inspection of funnel plots (Figures S2–S4). Summary‐of‐findings tables were generated accordingly to present the strength of ethnic and risk factor‐related associations. Sensitivity analyses were also conducted to further validate the robusticity of analytical approaches (Table S6).

### 2.5 Ethnicity and race grouping

Primary analyses used Tier 1 classifications based on standardized OMB categories: Asian, Black, White, and Hispanic populations (Table 1). Following Flanagin et al.^17^ recommendations, race and ethnicity were treated as sociocultural constructs requiring transparent, context‐specific classification.

**TABLE 1 alz70976-tbl-0001:** Study and participant characteristics by ethnic group.

Ethnicity group	Tier	*k*	*N*	Age (SD)	Female (%)	Countries
Overall		173	2,081,553	64.1 (7.4)	1,076,254 (51.7%)	
Alaska Native	1	1	749	72.8 (5.7)	507 (67.7%)	United States
Black	1	9	2428	67.6 (6.7)	1533 (63.1%)	Congo, UK, United States
Hispanic	1	10	3105	68.0 (7.4)	1920 (61.8%)	Brazil, Ecuador, United States, Venezuela
White	1	46	47,939	66.0 (7.1)	25,068 (52.3%)	Austria, Finland, France, Germany, Iceland, Netherlands, Norway, Ireland, Polish, Spain, Sweden, UK, United States
Asian	1	120	2,027,332	62.8 (7.5)	1,047,226 (51.7%)	Hong Kong, India, Japan, Mainland China, Malaysia, Singapore, South Korea, Taiwan, UK, United States
Alaska Native–United States	2	1	749	72.8 (5.7)	507 (67.7%)	United States
Black–Congo	2	1	77	71.6 (8.6)	37 (48.1%)	Congo
Black–UK	2	1	120	70.5 (8.5)	79 (65.8%)	UK
Black–United States	2	7	2231	66.7 (6.2)	1417 (63.5%)	United States
Hispanic–Brazil	2	1	177	82.8 (4.3)	60 (33.9%)	Brazil
Hispanic–Ecuador	2	3	886	67.4 (6.9)	504 (56.9%)	Ecuador
Hispanic–United States	2	5	1613	67.1 (7.1)	1040 (64.5%)	United States
Hispanic–Venezuela	2	1	429	59.3 (13.0)	316 (73.7%)	Venezuela
White–Austria	2	1	584	67.4 (9.2)	362 (62.0%)	Austria
White–Finland	2	1	152	70.6 (2.9)	95 (62.5%)	Finland
White–France	2	5	4847	67.0 (7.4)	2846 (58.7%)	France
White–Germany	2	10	14,153	62.5 (10.3)	6255 (44.2%)	Germany
White–Iceland	2	2	4432	77.5 (4.9)	2634 (59.4%)	Iceland
White–Netherlands	2	5	12,233	69.0 (7.6)	6461 (52.8%)	Netherlands
White–Norway	2	1	873	5.8 (4.2)	464 (53.2%)	Norway
White–Ireland	2	1	497	68.5 (7.5)	258 (51.9%)	Ireland
White–Polish	2	1	554	61.0 (5.6)	367 (66.2%)	Poland
White–Spain	2	2	671	59.0 (5.0)	405 (60.4%)	Spain
White–Sweden	2	7	3516	72.2 (5.5)	1878 (53.4%)	Sweden
White–UK	2	2	376	72.9 (5.9)	131 (34.8%)	United Kingdom
White–United States	2	8	5051	66.9 (6.3)	2912 (57.7%)	United States
Asian–Chinese–Hong Kong	2	4	2236	70.0 (5.5)	1415 (63.3%)	Hong Kong
Asian–Chinese–Mainland China	2	44	1,936,095	61.6 (8.1)	1,003,958 (51.9%)	Mainland China
Asian–Chinese–Taiwan	2	4	2154	64.8 (7.8)	1204 (55.9%)	Taiwan
Asian–Chinese–United States	2	1	720	59.8 (2.9)	414 (57.5%)	United States
Asian–Indian–India	2	1	2599	64.7 (6.4)	1207 (46.4%)	India
Asian–Japanese–Japan	2	38	48,181	63.9 (6.6)	24,041(49.9%)	Japan
Asian–Malay–Malaysia	2	1	54	39.6 (11.6)	37 (68.5%)	Malaysia
Asian–Malay–Singapore	2	1	276	70.9 (6.9)	153 (55.4%)	Singapore
Asian–South Asian–UK	2	2	333	76.7 (5.1)	141 (42.3%)	UK
Asian–South Korean–South Korea	2	24	34,684	61.4 (8.3)	14,656 (42.3%)	South Korea

*Note*: Summary of study distribution and participant demographics across 178 included studies (*N* = 2,081,553). Overall mean age was 64.1 years (SD = 7.4), with 51.7% female participants. Ethnic representation included Asian (*k* = 120; *N* = 2,027,332), White (*k* = 46; *N* = 47,939), Hispanic (*k* = 10; *N* = 3105), Black (*k* = 9; *N* = 2428), and Alaska Native (*k* = 1; *N* = 749) cohorts. Within Tier 2 stratifications, large samples were available for Mainland Chinese (*N* = 1,936,095), Japanese (*N* = 48,181), South Korean (*N* = 34,684), and White‐German (*N* = 14,153) populations. Smaller subgroups included South Asian participants in the UK (*N* = 333) and Malay participants in Malaysia (*N* = 54). Countries represented spanned Asia, Europe, and North and South America. Data summarised from the 178 studies included in this systematic review and meta‐analysis.

* *k*, number of studies per ethnic group.

^†^ N, total sample size per ethnic group.

^‡^ SD, standard deviation.

^§^ “Community/population based” = general‐population cohorts; “hospital/clinic routine check‐up” = clinical outpatient or screening populations.

^¶^ Countries listed refer to where study was conducted.

^#^ Income level classification based on World Bank criteria.

** T, Tesla; MRI, magnetic resonance imaging.

Asian populations included Chinese (Hong Kong, Mainland China, Taiwan, United States), Japanese, South Korean, Indian, Malay, and South Asian subgroups; Black populations included participants from Congo, UK, and the United States; White populations represented participants from Austria, Finland, France, Germany, Iceland, Netherlands, Norway, Ireland, Poland, Spain, Sweden, UK, and the United States; Hispanic populations included participants from Brazil, Ecuador, the United States, and Venezuela. Where sufficient studies existed (≥3 studies per subgroup), details are listed in Table S7.

Given the substantial Asian data availability, Tier 2 subgroup analyses were conducted within Asian ethnicities (Chinese subgroups, South Korean, and Japanese) where sufficient studies existed (≥3 studies per subgroup) (Table S8). Other ethnic groups with limited representation underwent narrative descriptions due to insufficient statistical power.

Lists of study meta‐analyses or narratively synthesized are detailed in supplementary tables, cross‐referenced using “NO_ID,” which is short for Number ID (Table S9).

### 2.6 Data analysis

All analyses were conducted using R metafor for meta‐regression model analysis and emmeans package to quantify mean differences between ethnicities.^18^, ^19^ For all analyses, we employed multilevel meta‐analysis models (rma.mv function) to account for the clustering of different Tier 1 or Tier 2 Asian‐only ethnic groups within the same study.

For CSVD[Table alz70976-tbl-0001] measures, continuous variables were analyzed using differences in the log‐transformed means (MNLN, function in metafor), including WMH volume (cubic centimeters [cm^3^]), WMH Fazekas grading was analyzed using logit‐transformed proportions (PLO function in metafor) and back‐transformed to odds of high WMH burden (number of participants with Fazekas ≥ 2) over total number of participants to better capture the odds of WMH burden category in a single measure. Categorical CSVD markers were analyzed using logit‐transformed prevalence (PLO), including CMBs, lacunes, and microinfarcts.

For cardiovascular and sociodemographic risk factors, continuous variables were analyzed using means, including age, education, blood pressure, cholesterol, and BMI. Categorical risk factors were assessed using logit‐transformed prevalence and included hypertension, diabetes, hyperlipidemia, and lifestyle factors.

Base models included ethnicity and study‐level age as covariates, while interaction models tested ethnicity moderation of the relationship between risk factor and CSVD measures (Ethnicity × Risk factor interactions). Likelihood ratio tests (LRTs) were used for model comparison. Between‐ethnic‐cohort heterogeneity was assessed with *I* ^2^ and *τ* ^2^ statistics. The *I* ^2^ statistic represents the percentage of total variance attributable to between‐study heterogeneity, with values of 0% to 25% indicating low heterogeneity, 25% to 50% indicating moderate heterogeneity, 50% to 75% indicating substantial heterogeneity, and >75% indicating high heterogeneity. The *τ* ^2^ statistic provides an estimate of the between‐study variance on the effect estimate scale. Key equations can be found in Section S3. Likelihood test ratio findings are listed in Table S10.

Ethnic differences were examined using three complementary approaches: (1) omnibus tests assessing whether ethnicity had a significant effect, (2) custom “one‐versus‐the‐rest” contrast testing using general linear hypothesis testing to compare each ethnicity against the pooled mean of all others, and (3) pairwise comparisons between ethnicities to identify specific group differences. The one‐versus‐the‐rest approach used a custom contrast matrix where diagonal elements were 1 and off‐diagonal elements were –1/(*n*–1), with multiple‐comparisons corrections applied using the Bonferroni method to maintain the family‐wise error rate at 5%. Comprehensive forest plots were generated to visualize ethnicity‐specific effect estimates with confidence intervals.

Sensitivity analyses assessed the robustness of findings by identifying influential studies using standardized residuals, recalculating effect estimates after removing potential outliers, and employing robust variance estimation to account for potential violations of model assumptions.

## 3 RESULTS

A total of 9084 records were identified across Embase, PubMed, Web of Science, and PsycInfo (Figure 1). After removing 3123 duplicates and adding one record, 5962 titles/abstracts were screened. Of these, 4858 were excluded. Full‐text review of 1101 studies led to the exclusion of 923 (e.g., no ethnic stratification, duplicate cohorts, wrong population, methodological focus, or unsuitable data), leaving 178 studies eligible.^20^, ^21^, ^22^, ^23^, ^24^, ^25^, ^26^, ^27^, ^28^, ^29^, ^30^, ^31^, ^32^, ^33^, ^34^, ^35^, ^36^, ^37^, ^38^, ^39^, ^40^, ^41^, ^42^, ^43^, ^44^, ^45^, ^46^, ^47^, ^48^, ^49^, ^50^, ^51^, ^52^, ^53^, ^54^, ^55^, ^56^, ^57^, ^58^, ^59^, ^60^, ^61^, ^62^, ^63^, ^64^, ^65^, ^66^, ^67^, ^68^, ^69^, ^70^, ^71^, ^72^, ^73^, ^74^, ^75^, ^76^, ^77^, ^78^, ^79^, ^80^, ^81^, ^82^, ^83^, ^84^, ^85^, ^86^, ^87^, ^88^, ^89^, ^90^, ^91^, ^92^, ^93^, ^94^, ^95^, ^96^, ^97^, ^98^, ^99^, ^100^, ^101^, ^102^, ^103^, ^104^, ^105^, ^106^, ^107^, ^108^, ^109^, ^110^, ^111^, ^112^, ^113^, ^114^, ^115^, ^116^, ^117^, ^118^, ^119^, ^120^, ^121^, ^122^, ^123^, ^124^, ^125^, ^126^, ^127^, ^128^, ^129^, ^130^, ^131^, ^132^, ^133^, ^134^, ^135^, ^136^, ^137^, ^138^, ^139^, ^140^, ^141^, ^142^, ^143^, ^144^, ^145^, ^146^, ^147^, ^148^, ^149^, ^150^, ^151^, ^152^, ^153^, ^154^, ^155^, ^156^, ^157^, ^158^, ^159^, ^160^, ^161^, ^162^, ^163^, ^164^, ^165^, ^166^, ^167^, ^168^, ^169^, ^170^, ^171^, ^172^, ^173^, ^174^, ^175^, ^176^, ^177^, ^178^, ^179^, ^180^, ^181^, ^182^, ^183^, ^184^, ^185^, ^186^, ^187^, ^188^, ^189^, ^190^, ^191^, ^192^, ^193^, ^194^, ^195^, ^196^, ^197^ The final dataset comprised 178 studies (Table 1), including 2,081,553 participants (mean age 64.10 years, SD = 7.40), of whom 51.7% were female.

### 3.1 Pooled analyses

Across studies (Table S11), pooled CSVD estimates were as follows: WMH volume 4.30 cm^3^ (95% CI: 3.38 to 5.48), odds of high WMH burden 0.27 (95% CI: 0.21 to 0.34), lacunes 12.30% (95% CI: 10.40 to 14.50), microinfarcts 13.20% (95% CI: 9.90 to 17.40), and CMBs 11.20% (95% CI: 9.60 to 13.00). Heterogeneity was high (*I* ^2^ ≥ 96.90%). Age was positively associated with all CSVD markers (*β* = 0.04 to 0.07, *p* ≤ 0.02).

Cardiovascular risk factors (Table S12) showed mean age 62.72 years (SD = 7.45, 95% CI: 60.64 to 64.87), SBP 130.92 mmHg (95% CI: 128.41 to 133.49), DBP 77.39 mmHg (95% CI: 76.09–78.73), cholesterol 190.46 mg/dL (95% CI: 185.20–195.84), HDL 1.35 mmol/L (95% CI: 1.26–1.44), and BMI 24.60 kg/m^2^ (95% CI: 23.83–25.37). Prevalences were: hypertension 33.10% (95% CI: 27.80–38.80), diabetes 13.30% (95% CI: 11.20–15.70), hyperlipidemia 33.40% (95% CI: 28.60–38.50), obesity 14.00% (95% CI: 10.80–18.00), high alcohol 27.70% (95% CI: 23.40–32.50), and smoking 15.40% (95% CI: 12.40–18.90). Heterogeneity remained high (*I^2^* ≥ 98.20%).

### 3.2 Tier 1 ethnic group differences

**FIGURE 2:**
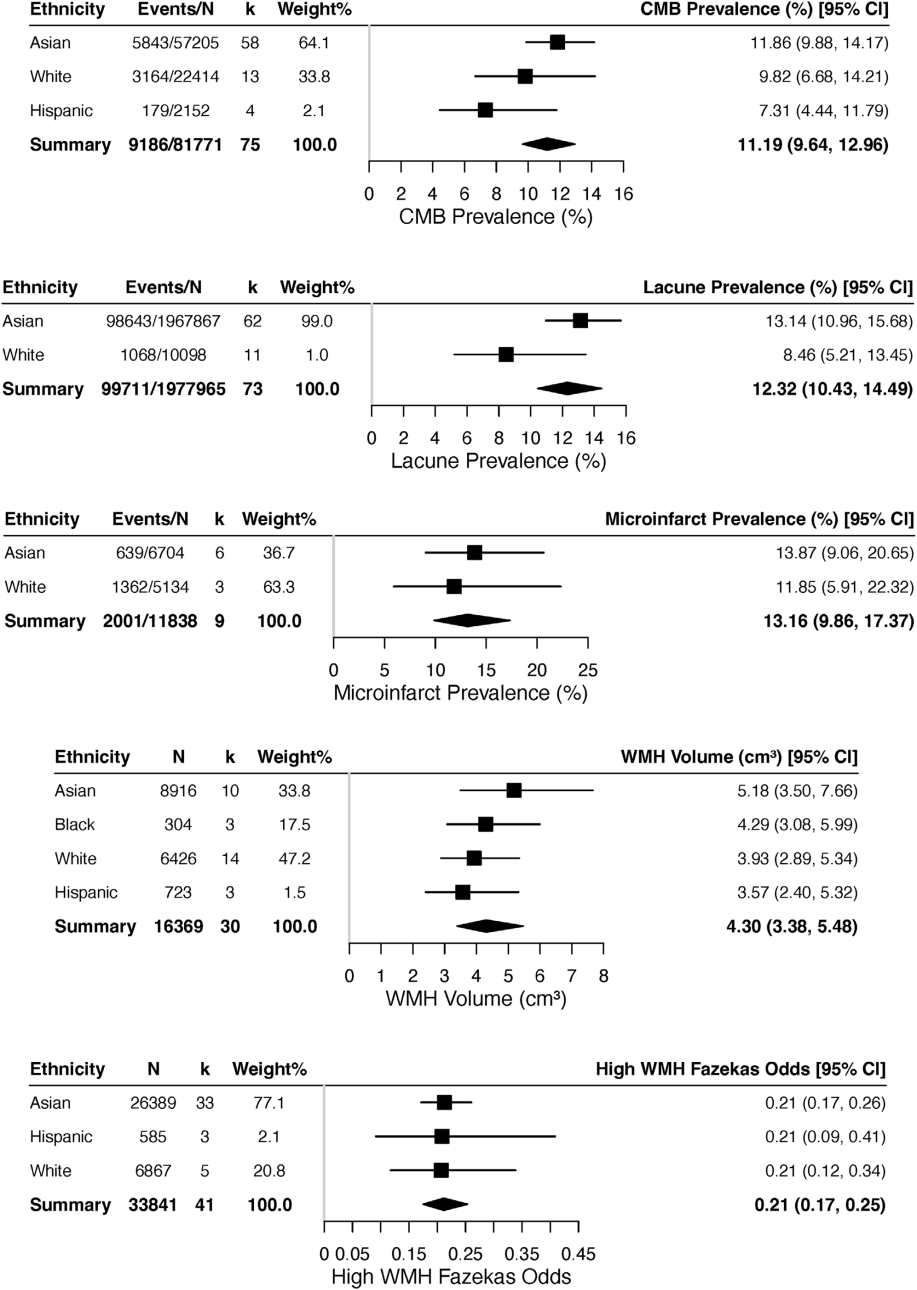
Forest plots of cerebral small vessel disease (CSVD) imaging markers by Tier 1 ethnic group. Pooled prevalence and mean estimates for CSVD markers, stratified by ethnicity. Cerebral microbleed (CMB) prevalence was highest among Asian participants (11.86%, 95% confidence interval [CI]: 9.88 to 14.17), followed by White (9.82%, 95% CI: 6.68 to 14.21) and Hispanic (7.31%, 95% CI: 4.44 to 11.79). Lacune prevalence was greater in Asian (13.14%, 95% CI: 10.96 to 15.68) than White (8.46%, 95% CI: 5.21 to 13.45) groups. Microinfarct prevalence was estimated at 13.87% (95% CI: 9.06 to 20.65) in Asian and 11.85% (95% CI: 5.91 to 22.32) in White cohorts. White matter hyperintensity (WMH) volume was highest among Asian participants (5.18 cm^3^, 95% CI: 3.50 to 7.66), compared to Black (4.29 cm^3^, 95% CI: 3.08 to 5.99), White (3.93 cm^3^, 95% CI: 2.89 to 5.34), and Hispanic (3.57 cm^3^, 95% CI: 2.40 to 5.32). Odds of high WMH burden were consistent across groups (Asian 0.21, 95% CI: 0.17 to 0.26; White 0.21, 95% CI: 0.12 to 0.34; Hispanic 0.21, 95% CI: 0.09 to 0.41).

**FIGURE 3:**
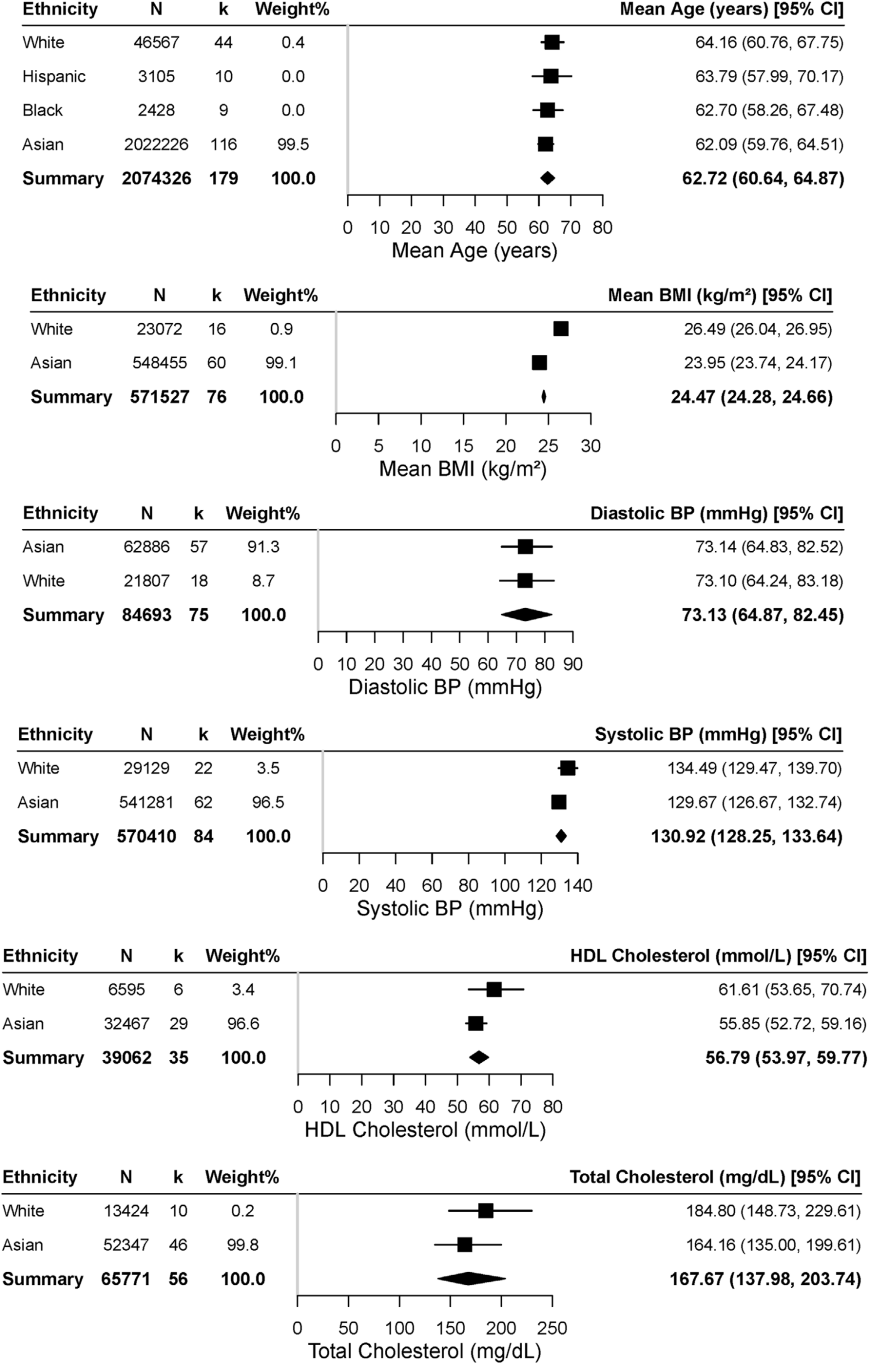
Forest plots of continuous risk factor and demographic and clinical characteristics by Tier 1 ethnic group. Meta‐analytic estimates of demographic and cardiometabolic variables across ethnic groups. Mean age at study entry was 62.72 years (95% confidence interval [CI]: 60.64 to 64.87), with Asian participants younger (62.09 years, 95% CI: 59.76 to 64.51) than White (64.16 years, 95% CI: 60.76 to 67.75), Hispanic (63.79 years, 95% CI: 57.99 to 70.17), and Black (62.70 years, 95% CI: 58.26 to 67.48). BMI was higher in White participants (26.49 kg/m^2^, 95% CI: 26.04 to 26.95) compared to Asian (23.95 kg/m^2^, 95% CI: 23.74 to 24.17). Blood pressure (BP) was elevated across groups, with diastolic BP at 73.13 mmHg (95% CI: 64.87 to 82.45) and systolic BP at 130.92 mmHg (95% CI: 128.25 to 133.64). Lipid levels showed lower high‐density lipoprotein in Asian (55.85 mmol/L, 95% CI: 52.72 to 59.16) compared to White (61.61 mmol/L, 95% CI: 53.65 to 70.74), while total cholesterol was higher in White (184.80 mg/dL, 95% CI: 148.73 to 229.61) than Asian (164.16 mg/dL, 95% CI: 135.00 to 199.61).

**FIGURE 4:**
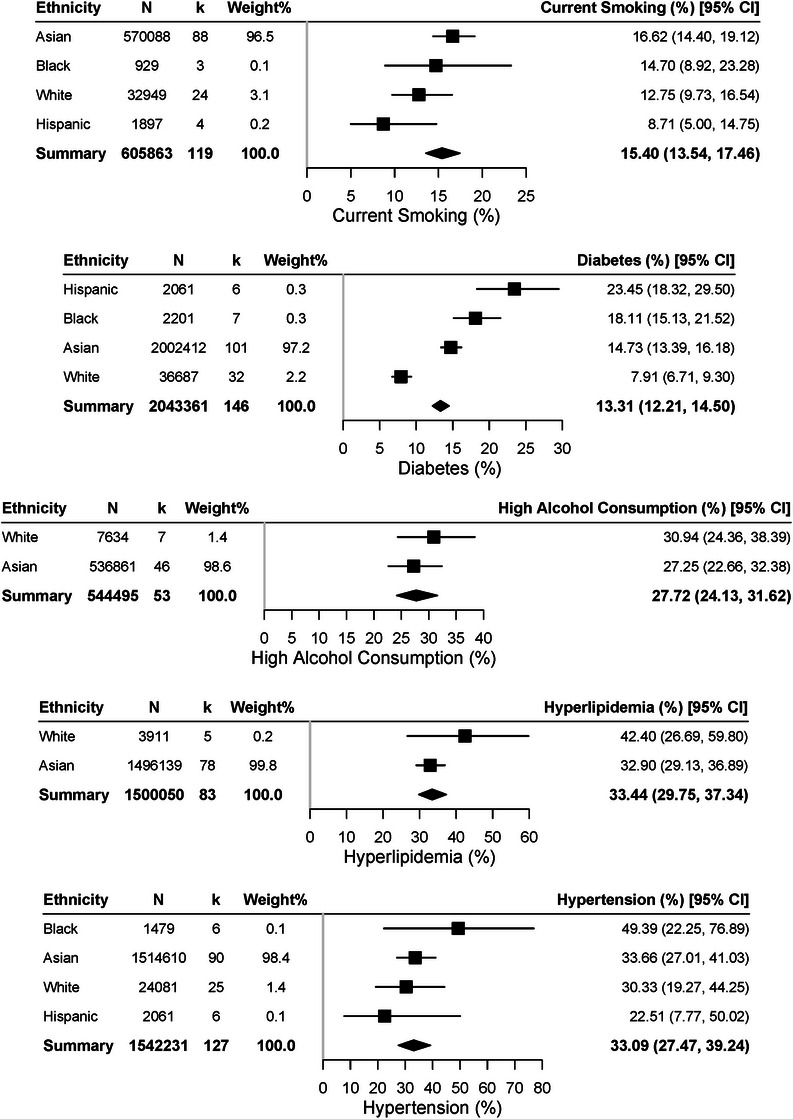
Forest plots of vascular risk factors prevalence by Tier 1 ethnic group. Pooled prevalence estimates of major vascular risk factors. Current smoking was most frequent among Asian participants (16.62%, 95% confidence interval [CI]: 14.40 to 19.12), with lower rates in White (12.75%, 95% CI: 9.73 to 16.54), Black (14.70%, 95% CI: 8.92 to 23.28), and Hispanic (8.71%, 95% CI: 5.00 to 14.75). Diabetes prevalence was highest in Hispanic (23.45%, 95% CI: 18.32 to 29.50), followed by Black (18.11%, 95% CI: 15.13 to 21.52), Asian (14.73%, 95% CI: 13.39 to 16.18), and White (7.91%, 95% CI: 6.71 to 9.30). High alcohol consumption was more common in White (30.94%, 95% CI: 24.36 to 38.39) than Asian (27.25%, 95% CI: 22.66 to 32.38). Hyperlipidemia was more frequent in White (42.40%, 95% CI: 26.89 to 59.80) than Asian (32.90%, 95% CI: 29.13 to 36.89). Hypertension was highest in Black participants (49.39%, 95% CI: 22.25 to 76.89), compared with Asian (33.66%, 95% CI: 27.01 to 41.03), White (30.33%, 95% CI: 19.27 to 44.25), and Hispanic (22.51%, 95% CI: 7.77 to 50.02.

Figures 2, 3, 4 show pooled comparisons. Asians had numerically higher CMB prevalence (11.86%, 95% CI: 9.88–14.17) and lacunes (13.14%, 95% CI: 10.96–15.68) than Whites (9.82% and 8.46%) and higher WMH volume (5.18 cm^3^, 95% CI: 3.50–7.66) than Hispanics (3.57 cm^3^, 95% CI: 2.40–5.32). Whites had higher BMI (26.49 kg/m^2^, 95% CI: 26.04–26.95), cholesterol (184.80 mg/dL, 95% CI: 148.73–229.61), and HDL (61.61 mmol/L, 95% CI: 53.65–70.74). Hispanics showed higher diabetes prevalence (23.45%, 95% CI: 18.32–29.50), Asians higher smoking (16.62%, 95% CI: 14.40–19.12), and Blacks higher hypertension (49.39%, 95% CI: 22.25–76.89).

Omnibus tests (Table S13) showed no significant ethnic differences in WMH volume, odds of high WMH burden, lacunes, or microinfarcts, with a trend for CMBs (QM = 5.65, *p* = 0.06). Pairwise contrasts (Table S14) showed Asians with numerically higher lacunes (OR = 1.64, 95% CI: 0.93–2.90, *p* = 0.09) and CMBs versus Whites, but non‐significant. One‐versus‐pooled tests (Table S15) indicated Asians with significantly higher CMB prevalence (OR = 1.45, 95% CI: 1.06–1.98, *p* = 0.019), Whites with numerically lower lacunes, and Hispanics with lower WMH volume.

For cardiovascular risks, omnibus tests showed significant ethnic differences in cholesterol, BMI, and diabetes (all *p* < 0.001). Pairwise contrasts confirmed higher diabetes in Asians (OR = 2.01, 95% CI: 1.58–2.55, *p* < 0.0001), Blacks (OR = 2.57, 95% CI: 2.12–3.13, *p* < 0.0001), and Hispanics (OR = 3.57, 95% CI: 2.46–5.16, *p* < 0.0001) compared with Whites. Asians also had lower cholesterol (OR = 0.88, 95% CI: 0.82–0.93, *p* < 0.0001) and BMI (OR = 0.90, 95% CI: 0.89–0.92, *p* < 0.0001). Other contrasts were non‐significant. One‐versus‐pooled tests showed Whites with higher cholesterol/BMI but lower diabetes, Asians lower cholesterol/BMI, Hispanics higher diabetes but lower smoking, and Blacks higher diabetes.

### 3.3 Tier 2 Asian subgroup differences

Subgroup analyses (Figures S5–S7) showed Mainland Chinese with higher CMB prevalence (14.42%, 95% CI: 11.45–18.00) and WMH volume (5.67 cm^3^, 95% CI: 2.26–14.26), and Japanese with higher lacunes (14.60%, 95% CI: 11.00–20.80). Hong Kong Chinese were older (67.23 years, 95% CI: 64.33–70.25) with higher diabetes (17.36%) and hypertension (50.34%). Mainland Chinese had higher BMI (24.61 kg/m^2^) and SBP (131.66 mmHg), Japanese higher HDL (61.13 mmol/L) and cholesterol (205.42 mg/dL), and Hong Kong Chinese higher smoking (20.99%).

Omnibus tests (Table S16) showed significant subgroup differences for CMBs (QM = 18.36, *p* < 0.001). Pairwise contrasts (Table S17) found Japanese with lower CMBs versus Hong Kong Chinese (OR = 0.48, *p* = 0.15) and Koreans with lower lacunes versus pooled (OR = 0.66, *p* = 0.40), though not significant. One‐versus‐pooled contrasts (Table S18) showed significantly higher CMBs in Mainland Chinese (14.20%, *p* = 0.007) and lower in Japanese (8.90%, *p* = 0.03).

For vascular risks, omnibus tests showed significant subgroup differences in age, cholesterol, HDL, BMI, and diabetes (all *p* ≤ 0.003). Pairwise contrasts indicated Hong Kong Chinese were older than Koreans (mean difference 8.6 years, *p* < 0.001) and Mainland Chinese (5.6 years, *p* = 0.001), and Mainland Chinese had lower cholesterol (−12.7 mg/dL, *p* < 0.001) and HDL (−0.23 mmol/L, *p* < 0.001) than Japanese. One‐versus‐pooled tests confirmed Hong Kong Chinese were older (70.0 vs. 66.0 years, *p* = 0.002), Koreans younger (61.4 vs. 66.7, *p* = 0.03), Japanese with lower BMI (23.0 vs. 24.3 kg/m^2^, *p* < 0.001) and lower diabetes prevalence (11.8% vs. 17.5%, OR = 0.65, 95% CI: 0.48–0.87, *p* = 0.005).

### 3.4 Interaction effects

Interaction meta‐regressions (Tables S19–S20; Figures S8–S9) evaluated whether the associations between vascular risk factors and CSVD markers differed by ethnicity.

In Tier 1, studies with higher proportions of women showed stronger associations with lacunes in Asians than in other groups (*β* = 0.10, SE = 0.046, *p* = 0.03). Each 10% increase in the proportion of females corresponded to 11% higher odds of lacunes in Asians (OR = 1.11, 95% CI: 1.01–1.21), compared with no meaningful change in other populations (OR = 1.00, 95% CI: 0.95–1.06). Hypertension prevalence was also more strongly related to lacunes in Asians than in other ethnic groups (*β* = 0.04, SE = 0.018, *p* = 0.03). For each 10% higher prevalence of hypertension, the odds of lacunes increased by 6% in Asians (OR = 1.06, 95% CI: 1.02–1.10), compared with 2% in other studies (OR = 1.02, 95% CI: 0.99–1.05). A trend‐level positive interaction was observed between diastolic blood pressure (DBP) and CMBs (*β* = 0.15, SE = 0.09, *p* = 0.10). Higher DBP was associated with greater microbleed burden in Asians (OR = 1.16, 95% CI: 0.97 to 1.39), whereas no such association was evident in other groups (OR = 1.00, 95% CI: 0.90 to 1.12).

In Tier 2 (East Asian subgroups), the association of SBP with odds of high WMH burden was stronger in South Koreans than in other Asian groups (*β* = 0.21, SE = 0.096, *p* = 0.03). A 10 mmHg higher SBP corresponded to 23% higher odds of severe WMH burden in South Korean cohorts (OR = 1.23, 95% CI: 1.02 to 1.49), compared with no clear change in others (OR = 1.00, 95% CI: 0.91 to 1.11). Diabetes prevalence showed a stronger association with high WMH burden in Mainland Chinese than in other Asian groups (*β* = 0.20, SE = 0.09, *p* = 0.03). Each 10% higher prevalence of diabetes was linked to 22% higher odds of severe WMH burden in Mainland Chinese cohorts (OR = 1.22, 95% CI: 1.02 to 1.46), compared with only 4% in other Asian studies (OR = 1.04, 95% CI: 0.94 to 1.16). A trend‐level interaction was also noted for BMI in South Koreans (*β* = 0.94, SE = 1.85, *p* = 0.61). Higher BMI was associated with numerically greater odds of high WMH burden in South Korean studies (OR ≈ 1.10, 95% CI: 0.80 to 1.50), compared with other Asian groups (OR ≈ 1.00, 95% CI: 0.90 to 1.11), although this difference was not statistically significant.

### 3.5 Narrative results for Tier 1 ethnic groups not meta‐analysed

In Alaska Natives, the Strong Heart Study reported lacunes at 21.90% (vs pooled 12.30%), with diabetes (48.86%) and obesity (54.21%) well above pooled estimates (13.90% and 19.40%) (NO_ID 328).^24^

Among Blacks, lesion prevalence was often above pooled. Healthy Aging in Neighborhoods of Diversity across the Life Span (HANDLS) cohort reported microinfarcts at 15.00% (pooled 13.20%) and diabetes 22.00% (pooled 13.90%) (NO_ID 462).^22^ A Congolese study found WMH volume 11.67 cm^3^ and prevalence 11.69% versus pooled 4.30 cm^3^ and 0.27 odds of high WMH burden, despite low vascular risks (NO_ID 4985).^32^ In Northern Manhattan Study (NOMAS) cohort, CMBs were 11.37% (pooled 11.20%), but hypertension (76.78%) and smoking (63.98%) far exceeded pooled 33.10% and 18.20% (NO_ID 5062).^25^

Hispanic cohorts showed heterogeneity. Maracaibo reported lacunes at 8.86% (vs pooled 12.30%) and CMBs 11.89% (pooled 11.20%) (NO_ID 121).^37^ Health and Aging Brain Study ‐ Health Disparities (HABS‐HD) cohort showed lower WMH volume (2.17 cm^3^) but markedly higher diabetes (35.73% vs 13.90%) (NO_ID 228).^30^ By contrast, Atahualpa and Pietà reported a WMH prevalence of 24% to 31% (above pooled odds of high WMH burden) with excess hypertension and diabetes (NO_ID 445^31^; NO_ID 527).^27^ The Los Angeles Latino Eye Study showed WMH volumes near pooled, but diabetes higher at 32% (NO_ID 252; [p201]).^23^ In NOMAS, CMBs were lower (5.21% vs 11.20%) yet vascular exposures exceeded pooled values (NO_ID 5062).^25^

Overall, Tier 1 cohorts showed lacune and WMH burdens often above pooled estimates, while CMBs and microinfarcts were closer to pooled. Hypertension and diabetes were consistently elevated (Table S12).

### 3.6 Narrative results for Tier 2 Asian subgroups not meta‐analyseed

In Hong Kong Chinese (CU‐RISK), WMH burden exceeded pooled Tier 2 estimates, with CMBs moderate but smoking > 35%, nearly double pooled 18.20% (NO_ID 482; ^36^; NO_IDs 314).^26^ Taiwanese Chinese (I‐Lan) showed higher WMH odds than pooled Tier 2, CMBs of 8% to 12% (consistent with pooled), and hypertension 36% to 44% (vs pooled 33.10%) (NO_ID 393; ^28^; NO_ID 686).^29^ In the US Chinese diaspora, lacunes were modest (6% to 8%) and WMHs low, but vascular risks were high (hypertension > 60%, diabetes > 20%, and smoking ∼25%) (NO_ID 4983^21^). Singaporean Chinese (EDIS) had lacunes of 13% to 14% (pooled 12.30%) with elevated smoking (22%) and hypertension (45%) (NO_ID 954).^34^

For South Asians in India, smoking was 28% (vs. pooled 18.20%), although CSVD markers were unavailable (NO_ID 4951; ^35^). Malaysian Malays showed high smoking (31.50%) without pooled CSVD data (NO_ID 134).^33^

Overall, Tier 2 subgroups showed higher WMHs in Hong Kong and Taiwanese Chinese, while lacunes and CMB burdens in diaspora and Singaporean cohorts were closer to pooled. Vascular risks – especially smoking, hypertension, and diabetes – were consistently higher than Tier 2 benchmarks, with largest excesses in Hong Kong, diaspora, and Malay cohorts (Table S18).

## 4 DISCUSSION

A comprehensive review and meta‐analysis of 178 studies and over two million participants revealed significant ethnic disparities in CSVD markers and vascular risk factors. Key CSVD markers – WMH volume, Fazekas grade, lacunes, CMBs, and microinfarcts – showed ethnic variation, as did cardiovascular risk factors such as cholesterol, BMI, and diabetes. These disparities highlight underlying biological and environmental mechanisms and underscore the need for precision medicine strategies.

In Tier 1 analyses, broad ethnic categories provided statistical power but revealed paradoxes. White populations had higher levels of traditional metabolic risks – SBP (134.49 mmHg), cholesterol (201.40 mg/dL), and BMI (26.49 kg/m^2^) – yet Asians showed a higher CMB burden. This discrepancy challenges a one‐to‐one link between risk exposure and CSVD pathology. Risk effects may be modified by treatment use, genetic context, or environment.^198^

This is strongly supported by findings from the Cohort Studies of Memory in an International Consortium (COSMIC). COSMIC demonstrated that the association between hypertension and stroke with WMH volumes was significantly stronger in White populations than in Asian populations^199^ and provided direct evidence that the effect of a vascular stressor is not uniform and may be modified by ethno‐regional context. Another COSMIC analysis of apolipoprotein E (*APOE*) ε4 further illustrates this complexity: while White ε4 carriers experienced faster decline in memory and Mini‐Mental State Examination (MMSE) scores, the effect in Asians was age‐dependent, showing accelerated memory decline only in those ∼80 years and older and slower MMSE decline once vascular risk factors were controlled.^200^ Our findings are consistent with the latest COSMIC synthesis,^201^ which concluded that ethno‐regional differences in risk factor effects had direct implications for dementia prevention. That analysis emphasized that interventions needed to be tailored to local contexts – for example, cardiovascular risk factors have a disproportionate impact in Asian populations, while social factors such as family interaction and social support play stronger protective roles in Asian than White cohorts. Placing our meta‐analysis alongside such global collaborative findings highlights both the mechanistic and population‐level reasons why “one‐size‐fits‐all” prevention strategies may be insufficient.

The Tier 2 analysis, a granular look at the largest available Asian subgroups, revealed a universe of distinct CSVD phenotypes that necessitate a move beyond a monolithic “Asian” category. This granular examination is crucial for developing targeted prevention strategies. The Chinese cohort, which included studies from Mainland China, Hong Kong, and Taiwan, for instance, exhibited a pronounced hemorrhagic profile. A granular examination revealed that Mainland Chinese populations had a significantly higher prevalence of CMBs (14.20%) compared to the pooled Asian mean (10.75%) and compared to Hong Kong (12.97%). Mainland Chinese populations also had the highest WMH volume and odds of high WMH burden among the Asian subgroups. This points to a pronounced vulnerability to microvascular damage, which manifests as both diffuse white matter pathology and small hemorrhagic lesions.^202^ This is further supported by studies linking hypertension and specific blood pressure measures to an increased risk of deep and infratentorial CMBs.^203^ This finding is underpinned by a confluence of genetic and environmental factors. For example, the HTRA1 gene, related to familial CSVD, has heterozygous mutations common in Chinese patients that can lead to increased vascular fragility.^204^ These findings blur the boundary between monogenic arteriopathies and sporadic CSVD, suggesting shared molecular pathways. However, most evidence comes from Japanese and Chinese populations, limiting generalizability. As highlighted by COSMIC,^201^ dementia genetics research remains heavily Eurocentric, underscoring the need for broader, multi‐ethnic genomic studies. Thus, while HTRA1 illustrates how local variants may contribute to ethnic differences in CSVD phenotypes, these associations should be interpreted with caution. While hypertension is a universal risk factor, genetic and environmental modifiers may accelerate this process in Chinese populations.^203^ Clinical evidence also confirms the elevated susceptibility of Chinese Canadians with diabetes and ischemic stroke to intracranial small vessel disease, particularly lacunar stroke and SVD.^205^ The pathological mechanism for this association is rooted in diabetes‐induced glucose and lipid metabolism disorders and systemic vascular activation, which result in widespread endothelial dysfunction and microcirculatory damage.^4^ Furthermore, high dietary sodium, common in traditional Chinese diets, has been shown to induce cerebral endothelial dysfunction and increase WMH volume independently of its effects on blood pressure.^206^ This layered explanation, which moves beyond the simple presence of traditional risk factors, provides context for the high hemorrhagic burden observed in this cohort. A contrasting finding from the meta‐analysis is that the prevalence of hypertension was considerably higher in the Hong Kong cohort (50.34%) than in the Mainland Chinese cohort (34.56%).

In contrast, Japanese cohorts presented with a numerically higher prevalence of lacunes, which are small, subcortical infarcts of presumed vascular origin.^207^ This indicates a different underlying pathology, with an “arteriosclerotic process” as the predominant mechanism.^1^ This aligns with external research identifying lacunar infarction as a frequent ischemic stroke subtype in some Japanese populations.^208^ Furthermore, studies on CSVD genomics have linked specific genes, such as COL4A2, to lacunar ischemic stroke.^209^ The finding that the Japanese cohort had a significantly healthier risk factor profile than other Asian subgroups further highlights that their CSVD pathology is less driven by the hemorrhagic burden that characterizes Chinese populations. A striking difference was found in the prevalence of CMBs, with Japanese populations having a significantly lower prevalence (8.90%, 95% CI: 6.50–12.10) compared to other Asian subgroups.^210^ The clinical implications of this distinction are significant, as a study in Japan showed that improved hypertension control was strongly linked to a decline in ischemic stroke incidence, demonstrating the direct clinical impact of addressing the primary risk factor for this phenotype.^211^

The findings from the Korean cohort add another layer of complexity. The analysis revealed a significant association between a higher mean SBP and odds of high WMH burden. This finding, observed in a cohort with a lower mean age (61.40 years) than many others, suggests an accelerated pathological process. This may be attributed to a combination of genetic predispositions and lifestyle factors such as elevated smoking rates in Korean men, which have been established as a driver of WMH progression in other populations.^204^ The distinct age‐related blood pressure increases observed in East Asian adults compared to other groups also point to unique risk trajectories that contribute to these disparities.^212^

The analysis also provided important insights into the disproportionate CSVD burden in Black and Hispanic populations. The meta‐analysis found that both Black (18.11%) and Hispanic (23.45%) populations had a significantly higher prevalence of diabetes compared to White populations (7.91%). This finding is consistent with established epidemiological data showing that Black and Hispanic adults with diabetes disproportionately experience microvascular complications compared to their White counterparts.^213^ The impact of these elevated risk factors appears to be compounded by other factors. Research in the UK Biobank, for example, demonstrates that Black individuals have an increased risk of dementia and stroke compared to White individuals, even after adjusting for traditional risk factors.^10^ The existence of multi‐ethnic cohorts within large‐scale consortia, such as the Stroke and Cognition (STROKOG) collaboration, provides a crucial context.^214^, ^215^ Furthermore, COSMIC consortium research suggests that, while dementia risk is higher in women than men, most risk factors appear to affect both sexes similarly.^216^ This indicates that the higher prevalence and severity of risk factors in underrepresented groups, exemplified by a peripheral artery disease rate that is twice as high as other ethnicities,^217^ may be amplified by social determinants of health and genetic factors that warrant further investigation.

The present study, while providing a valuable overview, is not without its limitations. It is worth noting that a major limitation of the existing literature is the paucity of genuinely multi‐ethnic studies that recruit participants from diverse backgrounds within the same cohort. Our comprehensive search, despite identifying a large number of studies, found that only 10 distinct cohorts (21 study entries) were genuinely multi‐ethnic, together comprising just 8826 participants (<0.5% of the total sample). The vast majority of studies are single‐population cohorts, making cross‐group comparisons vulnerable to confounding by differences in study methodologies, diagnostic criteria, and unseen participant‐level covariates. Consequently, the findings presented here should be considered hypothesis‐generating, not definitive.

A similar challenge was the substantial heterogeneity among the included studies, with *I* ^2^ values consistently exceeding 75% for CSVD markers. This heterogeneity stems from a variety of factors, including variability in MRI field strengths, lesion segmentation methods, and differences in cohort types and demographics. The reliance on study‐level data, while necessary for a meta‐analysis of this scale, inherently limits the ability to establish causal relationships between risk factors and CSVD lesions at an individual level. These limitations provide a powerful argument for the future direction of research, and the research community must move toward individual participant data meta‐analysis. Beneficial international data collaborations, exemplified by the COSMIC and ENIGMA consortium, have analyzed neuroimaging measures and risk factor data as well as genetic data to better understand their associations with late‐life cognition in diverse groups.^218^, ^219^ By pooling and harmonizing participant‐level data from their inception, such collaborations can better unravel the complex gene–environment interactions that drive ethnic disparities in brain health. The classification of ethnic and racial groups also poses a significant challenge, as these are sociocultural constructs that can mask significant intra‐group heterogeneity. This highlights the need for a more granular approach to classification in future studies that can more accurately reflect the true sources of biological and clinical variation.

### 4.1 Clinical implications

The findings have direct and immediate clinical implications, suggesting that instead of a one‐size‐fits‐all approach to CSVD prevention, the field must adopt ethnicity‐specific risk stratification and targeted interventions. For example, given the strong association between diabetes and WMHs in Chinese populations, combined with their elevated hemorrhagic risk, clinical management should focus not only on aggressive blood pressure and glucose control from an early age but also on patient‐specific dietary counselling aimed at reducing high sodium intake, a key contributor to cerebrovascular endothelial dysfunction.^220^ For Japanese populations, where lacunes are more prevalent, aggressive management of hypertension remains the paramount priority for preventing small vessel blockages and subsequent ischemic damage.^1^ For White populations, a multifaceted approach addressing the high burden of metabolic risk factors is essential for reducing overall CSVD burden. The findings also highlight the urgent need for tailored screening and prevention strategies for underrepresented groups, such as Black and Hispanic individuals, given their elevated diabetes burden and stroke risk, even after traditional risk factors are accounted for.

A critical consideration when interpreting the observed ethnic differences in risk–lesion associations is the distinction between biological (e.g., genetic susceptibility) and socio‐environmental mechanisms. While our interaction models reveal that a given vascular risk factor may have a differential effect size across ethnic groups, these differences should not be solely attributed to inherent genetic or biological variability. Instead, these findings may serve as a pragmatic marker of socio‐environmental differences, reflecting underlying differences in healthcare utilization, quality of care, and access to effective risk factor management. For instance, higher prevalence and stronger effects of hypertension in a particular group may reflect more severe, longer standing, or inadequately treated disease due to systemic barriers to care. These disparities in healthcare access and control of established risk factors must be thoroughly investigated as a potential cause for observed differences in CSVD burden and risk factor effects.

Based on our findings, we propose several testable hypotheses to guide future research on CSVD genomics and its implications across the lifespan^221^: (1) The elevated prevalence of CMBs in Chinese populations is primarily mediated by a combination of high dietary sodium intake and the presence of specific genetic variants (e.g., HTRA1 mutations) that increase vascular fragility, rather than solely by conventional hypertension.^222^ (2) In Japanese populations, the dominant CSVD phenotype shifts from lacunar infarcts to a more mixed WMH/CMB profile with aging, reflecting a transition from hypertensive arteriopathy to a broader microvascular pathology influenced by metabolic and lifestyle factors. (3) In Black and Hispanic populations, the excess burden of diabetes and other metabolic risk factors is compounded by systemic inflammation and social determinants of health, which are the primary drivers of the observed WMH burden and increased risk of dementia and stroke, independent of traditional vascular risk factors.

## 5 CONCLUSION AND FUTURE DIRECTIONS

This meta‐analysis provides a foundational population‐level framework for understanding ethnic disparities in CSVD. The findings demonstrate that ethnic groups do not share a uniform CSVD risk profile but rather exhibit distinct lesion phenotypes driven by a complex interplay of risk factor severity, genetics, and environmental exposures. The significantly higher prevalence of CMBs in Chinese populations, the elevated lacune burden in Japanese groups, and the pronounced metabolic risk factor profile in White populations all point to a need for precision prevention.

To address the current shortcomings and transform these hypotheses into definitive findings, multistudy collaborative harmonization efforts, such as previously discussed consortiums, are essential to standardize methodologies across existing and future cohorts. Ultimately, funding and efforts should be directed toward establishing new, multi‐ethnic cohorts that include persons from multiple ethnicities in the same study, which would allow for better control of shared environmental and methodological factors and inform the development of effective prevention strategies.

## AUTHOR CONTRIBUTIONS

Nikita K. Husein led the conceptualization, methodology, investigation, formal analysis, data curation, software development, visualization, validation, and project administration, conducted the literature search, created the figures, and wrote the original draft. Wei Wen, as NH's primary supervisor, contributed to conceptualization, methodology, supervision, validation, and review and editing, and directly verified the underlying data. Perminder S. Sachdev provided input on conceptualization, methodology, supervision and review and editing, and also verified the data. David K. E. Chan supported software development, formal analysis, data interpretation and methodology. John D. Crawford contributed to data interpretation and review and editing. Keshuo Lin acted as second reviewer for investigation and data curation. Jiyang Jiang offered early conceptual input and participated in review and editing.

## CONFLICT OF INTEREST STATEMENT

All authors declare that they have no conflicts of interest, financial or non‐financial, that could have influenced the work reported in this manuscript. PSS reports membership on advisory boards for Biogen and Roche Australia (2020–2022) and Eli Lilly (2025), unrelated to this work. No other authors have disclosures, financial or non‐financial, that could have appeared to influence this paper. Author disclosures are available in the Supporting Information.

## PROTOCOL AVAILABILITY STATEMENT

The full review protocol is available on PROSPERO (CRD42024518105). Two amendments were made to the statistical analysis plan: first to incorporate nested random‐effects models for certain risk factors based on likelihood ratio test results indicating a need to account for within‐study clustering and second to add ethnicity‐specific interaction analyses to examine relationships between risk factors and CSVD markers across ethnic groups. These amendments were documented in the PROSPERO registration.

## Supporting information



Supporting information

Supporting information

Supporting information

Supporting information

Supporting information

Supporting information

## Data Availability

Data and R code used in this study are available upon request from the corresponding author.
