# Supplementary material for: Global ethnic disparities in cerebral small vessel disease imaging markers and vascular risk factors: a systematic review and meta‐analysis
Source: Alzheimers Dement. 2026 Feb 9;22(2):e70976. doi: 10.1002/alz.70976 (PMC12884423; doi:10.1002/alz.70976)

Table of Contents

[1 Supplementary Table 1. PRISMA Guideline Checklist 1](#_Toc213921315)

[2 Supplementary Section 1. Search queries and database results 1](#_Toc213921316)

[2.1 Embase Search 1](#_Toc213921317)

[2.2 Supplementary Table 2 Web of Science 3](#_Toc213921318)

[3 Supplementary Table 3. Semi-quantitative White Matter Lesion measures 4](#_Toc213921319)

[4 Supplementary Table 4. Excluded at full-text screening stage and reasoning 3](#_Toc213921320)

[5 Supplementary Figure 1. Data Extraction Form Overview 3](#_Toc213921321)

[6 Supplementary Section 2. Risk of Bias Assessment- of Exposures (ROBANS-E) Criteria 7](#_Toc213921322)

[7 Supplementary Table 5. ROBANS-E Quality Assessment for included studies 8](#_Toc213921323)

[8 Supplementary Figure 2. CSVD- Publication Bias Funnel Plots 24](#_Toc213921324)

[9 Supplementary Figure 3. Prevalence Risk Factor- Publication Bias Funnel Plots 25](#_Toc213921325)

[10 Supplementary Figure 4. Continuous Risk Factor- Publication Bias Funnel Plots 26](#_Toc213921326)

[11 Supplementary Table 6. Sensitivity analyses 27](#_Toc213921327)

[12 Supplementary Table 7. Tier 1 Ethnicity Analyses based Study Inclusion ID Number 28](#_Toc213921328)

[13 Supplementary Table 8. Tier 2 Asian-only Ethnicity Analyses based Study Inclusion ID Number 28](#_Toc213921329)

[14 Supplementary Table 9. Full study Characteristics 28](#_Toc213921330)

[15 Supplementary Section 3. Key Equations 28](#_Toc213921331)

[16 Supplementary Table 10. Likelihood Test Ratio Findings for all outcomes 29](#_Toc213921332)

[17 Supplementary Table 11. Overall CSVD effect estimates 32](#_Toc213921333)

[18 Supplementary Table 12. Overall Risk Factor Effect estimates 32](#_Toc213921334)

[19 Supplementary Figure 5 Tier 2 Asian-only CSVD Outcome 34](#_Toc213921335)

[20 Supplementary Figure 6 Tier 2 Asian-only Continuous Risk Factor Outcome 35](#_Toc213921336)

[21 Supplementary Figure 7 Tier 2 Asian-only Prevalence Risk Factor Outcome 36](#_Toc213921337)

[22 Supplementary Table 13. Omnibus χ² Tests for Tier 1 Ethnicity Effects on CSVD Imaging Markers and Risk Factors 37](#_Toc213921338)

[23 Supplementary Table 14. Pairwise Tier 1 Ethnic Comparisons of CSVD Outcomes 37](#_Toc213921339)

[24 Supplementary Table 15. On versus All other Tier 1 Ethnic group contrasts Findings 39](#_Toc213921340)

[25 Supplementary Table 16. Omnibus χ² Tests for Tier 2 Asian Ethnicity Effects on CSVD Imaging Markers and Risk Factors 41](#_Toc213921341)

[26 Supplementary Table 17. Pairwise Tier 2 Asian Ethnic Comparisons 42](#_Toc213921342)

[27 Supplementary Table 18. On versus All other Tier 2 Asian Ethnic group contrasts Findings 45](#_Toc213921343)

[28 Supplementary Table 19. Tier 1 Ethnicity CSVD Interaction Model Analyses 48](#_Toc213921344)

[29 Supplementary Table 20. Tier 2 Asian CSVD Interaction Model Analyses 49](#_Toc213921345)

[30 Figure 8 Tier 1 Interaction analyses Plot 52](#_Toc213921346)

[31 Figure 9 Tier 2 Interaction analyses Plot 53](#_Toc213921347)

# **Supplementary Table 1. PRISMA Guideline Checklist**

| **Section and Topic** | **Item #** | **Checklist item** | **Location where item is reported** |
| --- | --- | --- | --- |
| **TITLE** |  |  |  |
| Title | 1 | Identify the report as a systematic review, meta-analysis, or both. | Title page, p.1 |
| **ABSTRACT** |  |  |  |
| Abstract | 2 | Provide a structured abstract including, as applicable: background; objectives; data sources; study eligibility criteria, participants, and interventions; study appraisal and synthesis methods; results; limitations; conclusions and implications of key findings. | Abstract, p.1–2 |
| **INTRODUCTION** |  |  |  |
| Rationale | 3 | Describe the rationale for the review in the context of what is already known. | Introduction, p.2–3 |
| Objectives | 4 | Provide an explicit statement of questions being addressed with reference to participants, interventions, comparisons, outcomes, and study design (PICOS). | Introduction, p.3 |
| **METHODS** |  |  |  |
| Protocol and registration | 5 | Indicate if a review protocol exists, if and where it can be accessed, and, if available, provide registration information including registration number. | Methods, p.3; Protocol availability, p.10 |
| Eligibility criteria | 6 | Specify study characteristics (e.g., PICOS, length of follow-up) and report characteristics (e.g., years considered, language, publication status) used as criteria for eligibility. | Methods, p.3–4 |
| Information sources | 7 | Describe all information sources (e.g., databases with dates of coverage, contact with study authors to identify additional studies) in the search and date last searched. | Methods, p.3; Supplementary Section 1 |
| Search | 8 | Present full electronic search strategy for at least one database, including any limits used. | Supplementary Section 1, Tables 2–3, p.1–4 |
| Study selection | 9 | State the process for selecting studies (i.e., screening, eligibility, included in systematic review, and, if applicable, included in the meta-analysis). | Methods, p.4; Figure 1 (Flowchart) |
| Data collection process | 10 | Describe method of data extraction from reports (e.g., piloted forms, independently, in duplicate) and any processes for obtaining and confirming data from investigators. | Methods, p.4; Supplementary Fig.1 |
| Data items | 11 | List and define all variables for which data were sought and any assumptions and simplifications made. | Methods, p.4; Supplementary Table 9 |
| Risk of bias in individual studies | 12 | Describe methods used for assessing risk of bias of individual studies and how this information is to be used in any data synthesis. | Methods, p.4; Supplementary Section 2 & Table 5 |
| Summary measures | 13 | State the principal summary measures (e.g., risk ratio, difference in means). | Methods, p.4–5 |
| Synthesis of results | 14 | Describe the methods of handling data and combining results of studies, including measures of consistency (e.g., I²) for each meta-analysis. | Methods, p.5; Supplementary Section 3 |
| Risk of bias across studies | 15 | Specify any assessment of risk of bias that may affect the cumulative evidence (e.g., publication bias, selective reporting within studies). | Methods, p.5; Supplementary Figs.2–4 |
| Additional analyses | 16 | Describe methods of additional analyses (e.g., sensitivity or subgroup analyses, meta-regression), if done, indicating which were pre-specified. | Methods, p.5; Supplementary Tables 6, 13–20 |
| **RESULTS** |  |  |  |
| Study selection | 17 | Give numbers of studies screened, assessed for eligibility, and included in the review, with reasons for exclusions at each stage, ideally with a flow diagram. | Results, p.5–6; Figure 1 |
| Study characteristics | 18 | For each study, present characteristics for which data were extracted and provide the citations. | Results, p.6; Table 1; Supplementary Table 9 |
| Risk of bias within studies | 19 | Present data on risk of bias of each study and, if available, any outcome level assessment. | Results, p.9; Supplementary Table 5 |
| Results of individual studies | 20 | For all outcomes considered, present, for each study: simple summary data for each intervention group and effect estimates and confidence intervals, ideally with a forest plot. | Results, p.6–8; Figures 2–4; Supplementary Tables 11–12 |
| Synthesis of results | 21 | Present results of each meta-analysis done, including confidence intervals and measures of consistency. | Results, p.6–8; Supplementary Tables 11–12 |
| Risk of bias across studies | 22 | Present results of any assessment of risk of bias across studies. | Results, p.9; Discussion p.9; Supplementary Figs.2–4 |
| Additional analysis | 23 | Give results of additional analyses, if done (e.g., sensitivity or subgroup analyses, meta-regression). | Results, p.8; Supplementary Tables 13–20 |
| **DISCUSSION** |  |  |  |
| Summary of evidence | 24 | Summarize the main findings including the strength of evidence for each main outcome; consider their relevance to key groups (e.g., healthcare providers, users, and policy makers). | Discussion, p.9–10 |
| Limitations | 25 | Discuss limitations at study and outcome level (e.g., risk of bias), and at review-level (e.g., incomplete retrieval of identified research, reporting bias). | Discussion, p.9–10 |
| Conclusions | 26 | Provide a general interpretation of the results in the context of other evidence, and implications for future research. | Discussion, p.9–10 |
| **FUNDING** |  |  |  |
| Funding | 27 | Describe sources of funding for the systematic review and other support; role of funders for the systematic review. | Funding section, p.10 |

1. *Item #*: PRISMA checklist item number.
2. *PRISMA*: Preferred Reporting Items for Systematic Reviews and Meta-Analyses

# **Supplementary Section 1. Search queries and database results**

## **Embase Search**

<https://login.wwwproxy1.library.unsw.edu.au/login?url=http://ovidsp.ovid.com/ovidweb.cgi?T=JS&NEWS=N&PAGE=main&SHAREDSEARCHID=G9qyh5Cc6zRiXhPDJMeCVNMCbjXWPFgvuotx9Dhb35rkkS1TbSwdIDVrRdwcyvrq>

Embase Classic+Embase <1947 to 2025 July 31>

1 small vessel disease*.mp. [mp=title, abstract, heading word, drug trade name, original title, device manufacturer, drug manufacturer, device trade name, keyword heading word, floating subheading word, candidate term word] 11882

2 *Binswanger encephalopathy/ or binswanger* disease*.mp. [mp=title, abstract, heading word, drug trade name, original title, device manufacturer, drug manufacturer, device trade name, keyword heading word, floating subheading word, candidate term word] 677

3 ((Microinfarct* or microscopic infarct* or microangiopath*) and (brain or cerebr*)).mp. [mp=title, abstract, heading word, drug trade name, original title, device manufacturer, drug manufacturer, device trade name, keyword heading word, floating subheading word, candidate term word] 7513

4 exp Leukoaraiosis/ or exp White matter lesion/ or (white matter hyperintens* or white matter disease* or white matter lesion* or white matter change* or changes in white matter or white matter damage or WML or WMH or leu?araiosis).mp. [mp=title, abstract, heading word, drug trade name, original title, device manufacturer, drug manufacturer, device trade name, keyword heading word, floating subheading word, candidate term word] 37021

5 exp Perivascular space/ or (peri?vascular space* or virchow?robin space* or etat crible or type 3 lacun*).mp. [mp=title, abstract, heading word, drug trade name, original title, device manufacturer, drug manufacturer, device trade name, keyword heading word, floating subheading word, candidate term word] 6637

6 ((Micro?bleed* or micro?h?emorrhag* or ("dot-like" and (suscept* or h?emosid*))) and (brain or cerebr* or cerebral small vessel disease*)).mp. [mp=title, abstract, heading word, drug trade name, original title, device manufacturer, drug manufacturer, device trade name, keyword heading word, floating subheading word, candidate term word] 7916

7 (Lacun* or subcortical infarct* or subcortical cystic infarct* or subcortical lesion*).mp. [mp=title, abstract, heading word, drug trade name, original title, device manufacturer, drug manufacturer, device trade name, keyword heading word, floating subheading word, candidate term word] 30337

8 exp ethnicity/ or exp race/ or exp "ethnic or racial aspects"/ or exp ethnic group/ or exp racial background/ or exp racial identity/ or exp racial diversity/ 630096

9 exp South Asian/ or exp British Asian/ or exp Southeast Asian/ or exp Central Asian/ or exp Asian continental ancestry group/ or exp "Asian American, Native Hawaiian and Pacific Islander"/ or exp East Asian/ or exp Asian American/ or exp Asian/ or exp West Asian/ or exp North Asian/ 331814

10 exp "Japanese (people)"/ or exp Chinese/ or exp "Korean (people)"/ or exp African American/ or exp African/ or exp Indian/ or exp American Indian/ or exp Pakistan/ or exp Singaporean/ or exp Singapore/ or exp Hispanic/ or exp South American/ or exp South America/ or exp middle east/ 1167251

11 exp Caucasian/ or (white* or caucasian* or "european descen*" or "european ancestr*" or "non-hispanic white*" or "euro-american*").mp. [mp=title, abstract, heading word, drug trade name, original title, device manufacturer, drug manufacturer, device trade name, keyword heading word, floating subheading word, candidate term word] 957731

12 8 or 9 or 10 or 11 2427672

13 2 or 3 or 4 or 5 or 6 or 7 78697

14 1 and 12 and 13 5220

15 14 and "Preprint".sa_pubt. 63

16 14 and "Article".sa_pubt. 3005

17 15 or 16 3068

**Pubmed**

**(small vessel disease OR "Cerebral Small Vessel Diseases"[Mesh]) AND (("Binswanger encephalopathy" OR "binswanger disease" OR (("Microinfarct" OR "microscopic infarct" OR "microangiopath") AND (brain OR cerebr)) OR Leukoaraiosis[Mesh] OR "Leukoencephalopathies"[Mesh] OR white matter hyperintens OR "white matter disease" OR "white matter lesion" OR "white matter change" OR "changes in white matter" OR "WML" OR "WMH" OR Perivascular space[Mesh] OR "perivascular space" OR "virchow-robin space" OR (("Microbleed" OR "microhemorrhage" OR ("dot-like" AND (suscept OR hemosid)) AND (brain OR cerebr OR "cerebral small vessel disease")) OR "Stroke, Lacunar"[Mesh] OR "Lacun" OR "subcortical infarct")) AND (Ethnic Groups[mh] OR Race[mh] OR "Ethnicity"[Mesh] OR ethnic or racial aspects OR "ethnic group" OR "racial background" OR "racial identity" OR "racial diversity" OR "White People"[Mesh] OR "Asian"[Mesh] OR "Asian American Native Hawaiian and Pacific Islander"[Mesh] OR "Hispanic or Latino"[Mesh] OR "Caribbean Hispanic people" [Supplementary Concept] OR "American Indian or Alaska Native"[Mesh] OR "Indians, North American"[Mesh] OR "Indians, Central American"[Mesh] OR "Indians, South American"[Mesh] OR "South Asian People"[Mesh] OR "Middle Eastern People"[Mesh] OR "African People"[Mesh] OR "South Asian" OR "British Asian" OR "Southeast Asian" OR "Central Asian" OR "Asian continental ancestry group" OR "Asian American, Native Hawaiian and Pacific Islander" OR "East Asian" OR "Asian American" OR "Asian" OR "West Asian" OR "North Asian" OR "Japanese (people)" OR "Chinese" OR "Korean (people)" OR "African American" OR "African" OR "Indian" OR "American Indian" OR "Pakistan" OR "middle east" OR "Singaporean" OR "Singapore" OR "Hispanic" OR "South American" OR "South America" OR "Caucasian" OR white OR caucasian OR "european descent" OR "european ancestry" OR "non-hispanic white" OR "euro-american") NOT booksdocs[Filter] NOT clinicaltrial[Filter] NOT meta-analysis[Filter] NOT randomizedcontrolledtrial[Filter] NOT review[Filter] NOT systematicreview[Filter] 2873**

**PsycInfo**

<https://login.wwwproxy1.library.unsw.edu.au/login?url=http://ovidsp.ovid.com/ovidweb.cgi?T=JS&NEWS=N&PAGE=main&SHAREDSEARCHID=1CTDl8SnKNdwFusxjZQBz6fdvFZWkKGAcNtC0e5b09Bc2leI38kSQZswvileAzyzz>

APA PsycInfo <1806 to July 2025 Week 4>

1 exp Cerebral Small Vessel Disease/ 731

2 *"Binswanger encephalopathy"/ or "binswanger* disease*".mp. 95

3 ((Microinfarct* or "microscopic infarct*" or microangiopath*) and (brain or cerebr*)).mp. 377

4 exp white matter/ or exp leukoaraiosis/ or ("white matter hyperintens*" or "white matter disease*" or "white matter lesion*" or "white matter change*" or "changes in white matter" or "white matter damage" or WML or WMH or leu?araiosis).mp. 15588

5 ("peri?vascular space*" or "virchow?robin space*" or "etat crible" or "type 3 lacun*").mp. 532

6 (Micro?bleed* or micro?h?emorrhag* or (dot-like and (suscept* or h?emosid*))).mp. and ((brain or cerebr*).mp. or exp cerebral small vessel disease/) 968

7 (Lacun* or "subcortical infarct*" or "subcortical cystic infarct*" or "subcortical lesion*").mp. 3845

8 exp ethnicity/ or exp "ethnic or racial aspects"/ or exp "ethnic group"/ or exp "racial background"/ or exp "racial identity"/ or exp "racial diversity"/ 201145

9 exp "South Asian"/ or exp "Southeast Asian"/ or exp "Central Asian"/ or exp "Asian continental ancestry group"/ or exp "Asian American, Native Hawaiian and Pacific Islander"/ or exp "East Asian"/ or exp "Asian American"/ or exp Asian/ or exp "West Asian"/ or exp "North Asian"/ 38451

10 exp Chinese/ or exp "Korean (people)"/ or exp "African American"/ or exp African/ or exp Indian/ or exp "American Indian"/ or exp Pakistan/ or exp Singaporean/ or exp Singapore/ or exp Hispanic/ or exp "South American"/ or exp "South America"/ or exp "middle east"/ 87998

11 exp Caucasian/ or (white* or caucasian* or "european descen*" or "european ancestr*" or "non-hispanic white*" or euro-american*).mp. 166304

12 2 or 3 or 4 or 5 or 6 or 7 19789

13 8 or 9 or 10 or 11 321091

14 1 and 12 and 13 428

## **Supplementary Table 2 Web of Science**

<https://www.webofscience.com/wos/woscc/summary/02ebab8b-e017-486e-9eff-1e55505d3568-01722f82d2/relevance/1>

| # | Search Query | Database | Results | Date Run |
| --- | --- | --- | --- | --- |
| 1 | TS=("small vessel disease" OR "cerebral small vessel disease") | Web of Science Core Collection | 10219 | Mon Aug 04 2025 21:37:41 GMT+1000 (Australian Eastern Standard Time) |
| 2 | TS=(ethnic* OR race OR racial* OR ancestry OR "population group*" OR white OR caucasian OR "european descen*" OR "european ancestr*" OR "non-hispanic white" OR "euro-american" OR black* OR "non-hispanic black" OR "african american*" OR "american indian*" OR "alaska native*" OR amerindian* OR "native american*" OR hispanic* OR latino* OR "caribbean hispanic*" OR "pacific islander*" OR hawaiian* OR asian* OR "east asian*" OR "south asian*" OR "southeast asian*" OR "central asian*" OR "asian american*" OR chinese OR japan* OR korea* OR singapor* OR malaysia* OR filipin* OR india*) | Web of Science Core Collection | 4720679 | Mon Aug 04 2025 21:40:21 GMT+1000 (Australian Eastern Standard Time) |
| 3 | TS=(binswanger* OR microinfarct* OR microangiopath* OR leukoaraiosis OR leukoencephalopath* OR "white matter hyperintens*" OR "white matter disease" OR "white matter lesion*" OR "white matter change*" OR WML OR WMH OR "perivascular space*" OR "virchow-robin*" OR microbleed* OR microhemorrhag* OR microhaemorrhag* OR lacun* OR "subcortical infarct*") | Web of Science Core Collection | 86923 | Mon Aug 04 2025 21:40:31 GMT+1000 (Australian Eastern Standard Time) |
| 4 | #1 AND #2 AND #3 AND DT=(ARTICLE OR "EARLY ACCESS") | Web of Science Core Collection | 4126 | Mon Aug 04 2025 21:40:43 GMT+1000 (Australian Eastern Standard Time) |

#: sequence number of search queries.
 Results: number of records retrieved.
 Date Run: date when the search was executed.

# **Supplementary Table 3. Semi-quantitative White Matter Lesion measures**

| NO_ID | Study_ID | Title | WMH/WML Scale Type |
| --- | --- | --- | --- |
| 89 | Nakazawa 2023 | Association of white matter lesions and brain atrophy with the development of dementia in a community: the Hisayama Study | Volumetric WMH (manual or automated) |
| 120 | Chu 2022 | Subclinical hypothyroidism is associated with basal ganglia enlarged perivascular spaces and overall cerebral small vessel disease load | Unclear/other |
| 128 | Jung 2022 | Relationship of the Triglyceride-Glucose Index with Subclinical White Matter Hypersensitivities of Presumed Vascular Origin Among Community-Dwelling South Koreans | Unclear/other |
| 170 | Yoshiura 2022 | Brain structural alterations and clinical features of cognitive frailty in Japanese community-dwelling older adults: the Arao study (JPSC-AD) | Shinohara classification |
| 257 | Buhrmann 2021 | Cerebellar Grey Matter Volume in Older Persons Is Associated with Worse Cognitive Functioning | ARWMC scale |
| 273 | Zhang 2021 | Association of Carotid Atherosclerosis With White Matter Hyperintensity in an Asymptomatic Japanese Population: A Cross-Sectional Study | Unclear/other |
| 393 | Chou 2019 | Location-Specific Association Between Cerebral Microbleeds and Arterial Pulsatility | Unclear/other |
| 423 | Ding 2018 | White Matter Hyperintensity Predicts the Risk of Incident Cognitive Decline in Community Dwelling Elderly | ARWMC scale |
| 451 | Ao 2018 | Large Vessel Disease Modifies the Relationship Between Kidney Injury and Cerebral Small Vessel Disease | Unclear/other |
| 474 | Higuchi 2017 | Visceral-to-subcutaneous fat ratio is independently related to small and large cerebrovascular lesions even in healthy subjects | Unclear/other |
| 561 | Power 2015 | Smoking and white matter hyperintensity progression The ARIC-MRI Study | CHS scale |
| 561 | Power 2015 | Smoking and white matter hyperintensity progression The ARIC-MRI Study | CHS scale |
| 586 | Yamashiro 2014 | Visceral fat accumulation is associated with cerebral small vessel disease | ARIC scale |
| 604 | Aarts 2014 | Inhibition of Serotonin Reuptake by Antidepressants and Cerebral Microbleeds in the General Population | Volumetric WMH (manual or automated) |
| 654 | Takahashi 2012 | Relationship Between Chronic Kidney Disease and White Matter Hyperintensities on Magnetic Resonance Imaging | Study-specific ordinal/binary |
| 663 | Nakano 2012 | Augmentation index is related to white matter lesions | PICA scale |
| 681 | Kim 2011 | Age-independent association of pulse pressure with cerebral white matter lesions in asymptomatic elderly individuals | Volumetric WMH (manual or automated) |
| 682 | Kim 2011 | Advanced Coronary Artery Calcification and Cerebral Small Vessel Diseases in the Healthy Elderly | Volumetric WMH (manual or automated) |
| 711 | Fornage 2008 | Biomarkers of inflammation and MRI-defined small vessel disease of the brain - The Cardiovascular Health Study | Volumetric WMH (manual or automated) |
| 711 | Fornage 2008 | Biomarkers of inflammation and MRI-defined small vessel disease of the brain - The Cardiovascular Health Study | Volumetric WMH (manual or automated) |
| 753 | Coomans 2023 | Interactions between vascular burden and amyloid-ÃŽÂ² pathology on trajectories of tau accumulation | Quantitative segmentation (LST/FreeSurfer) |
| 758 | Park 2023 | Classification of white matter lesions and characteristics of small vessel disease markers | STRIVE criteria |
| 816 | Hada 2020 | Periodontal Condition Is Correlated with Deep and Subcortical White Matter Hyperintensity Lesions in Japanese Adults | Japanese Brain Dock guidelines |
| 918 | Choi 2013 | Arterial Stiffness Using Cardio-Ankle Vascular Index Reflects Cerebral Small Vessel Disease in Healthy Young and Middle Aged Subjects | Volumetric WMH (manual or automated) |
| 923 | Kim 2012 | Association of obesity with cerebral microbleeds in neurologically asymptomatic elderly subjects | Study-specific ordinal/binary |

# **Supplementary Table 4. Excluded at full-text screening stage and reasoning**

Separate file provided

# **Supplementary Figure 1. Data Extraction Form Overview**


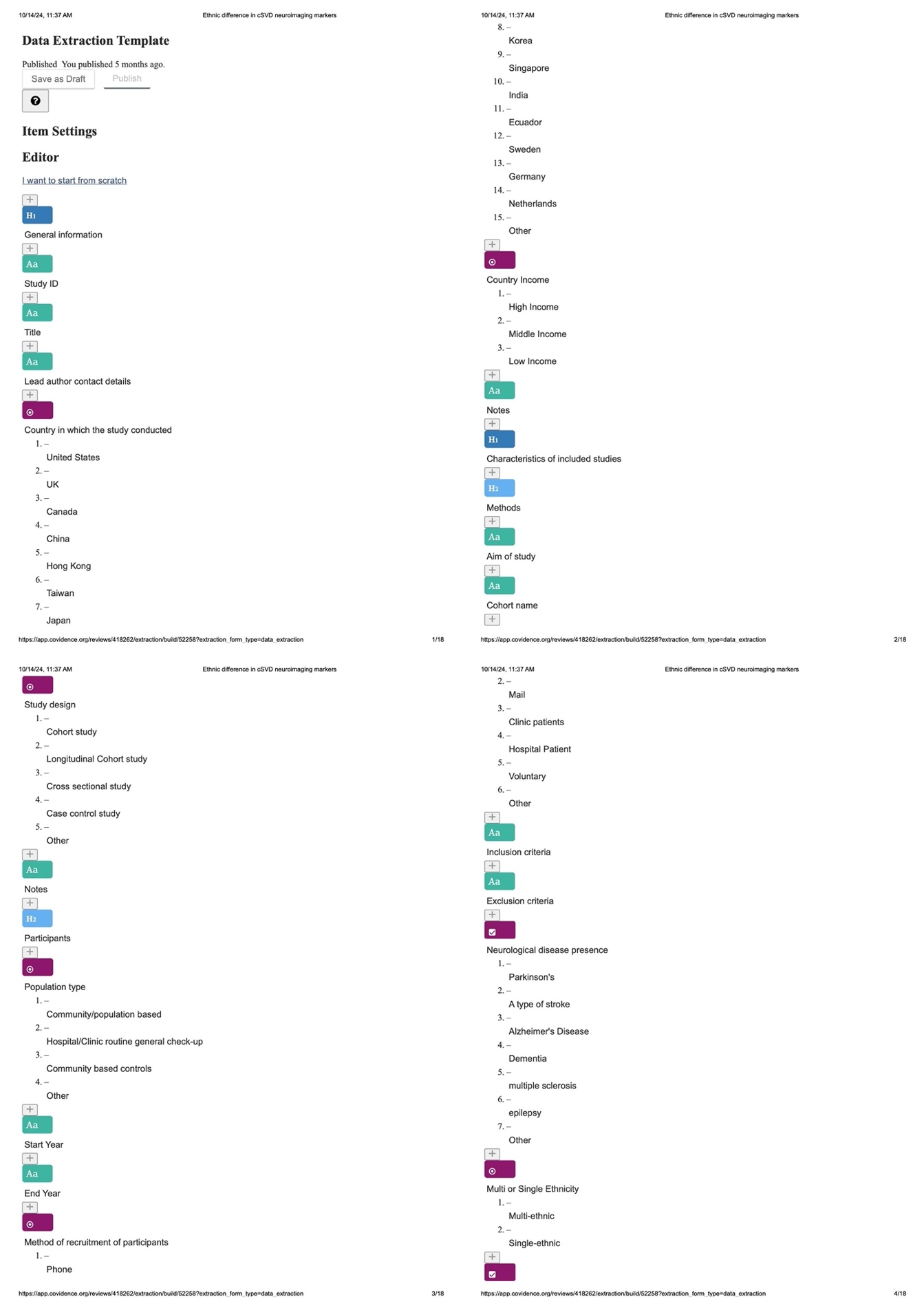


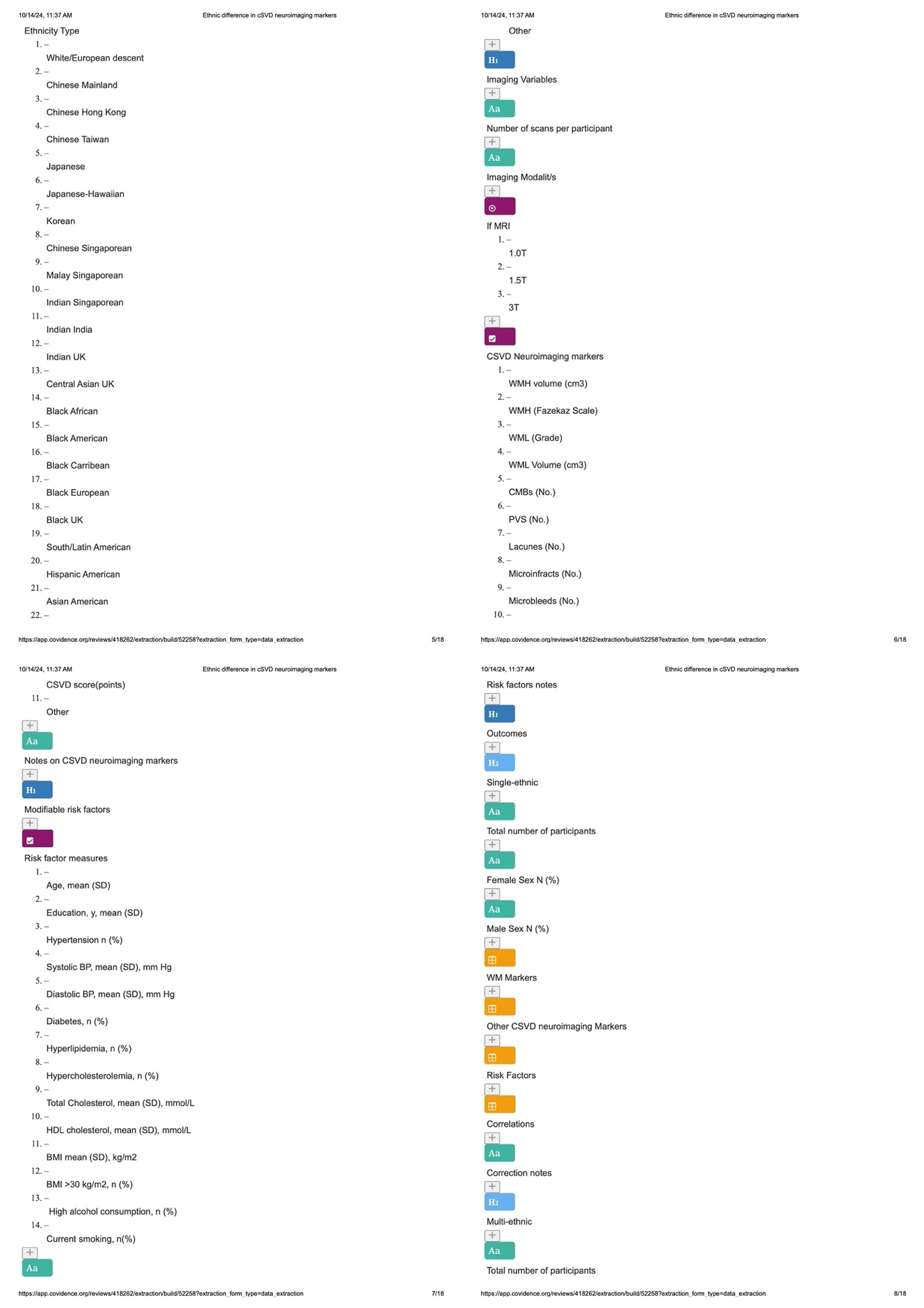


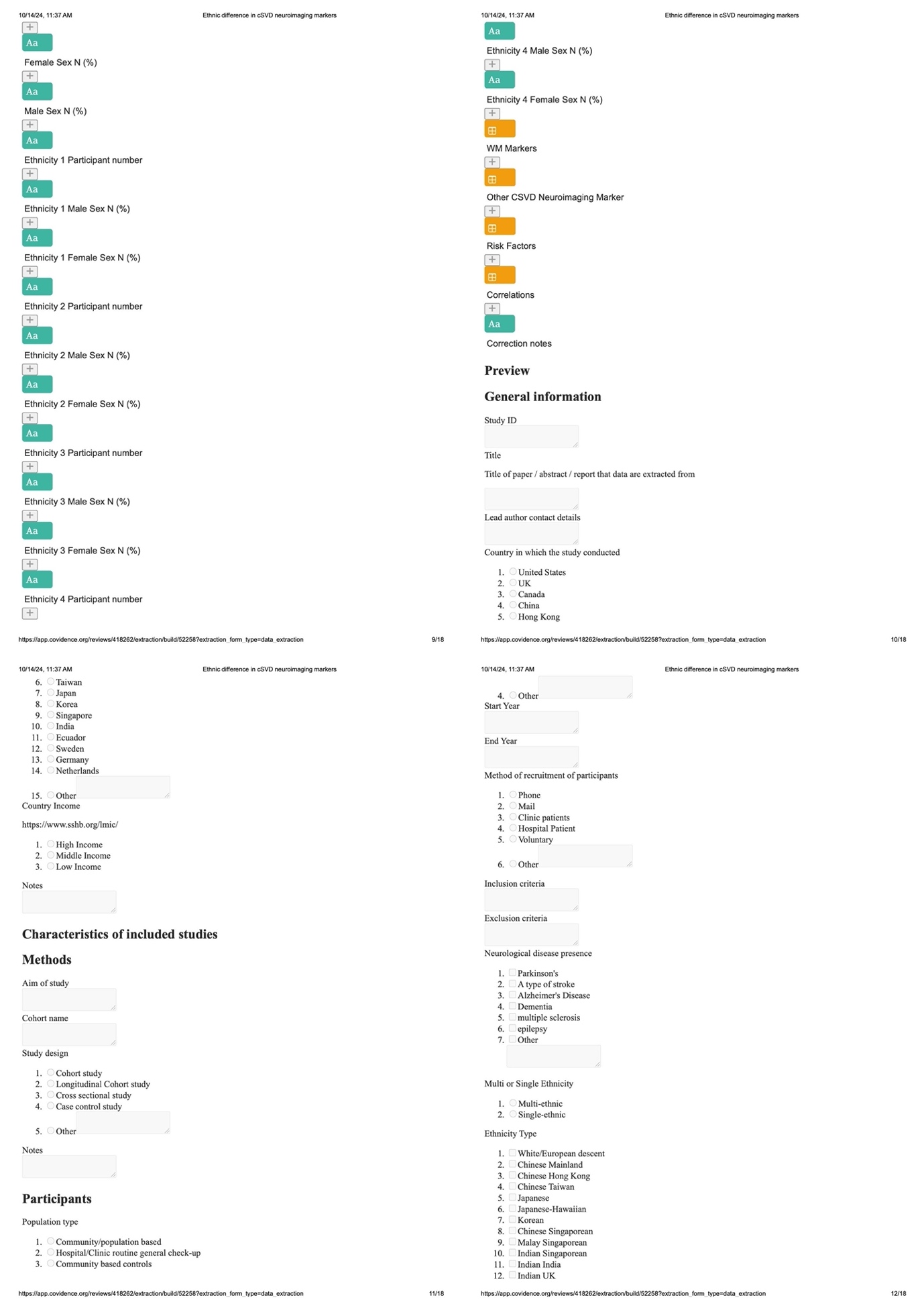


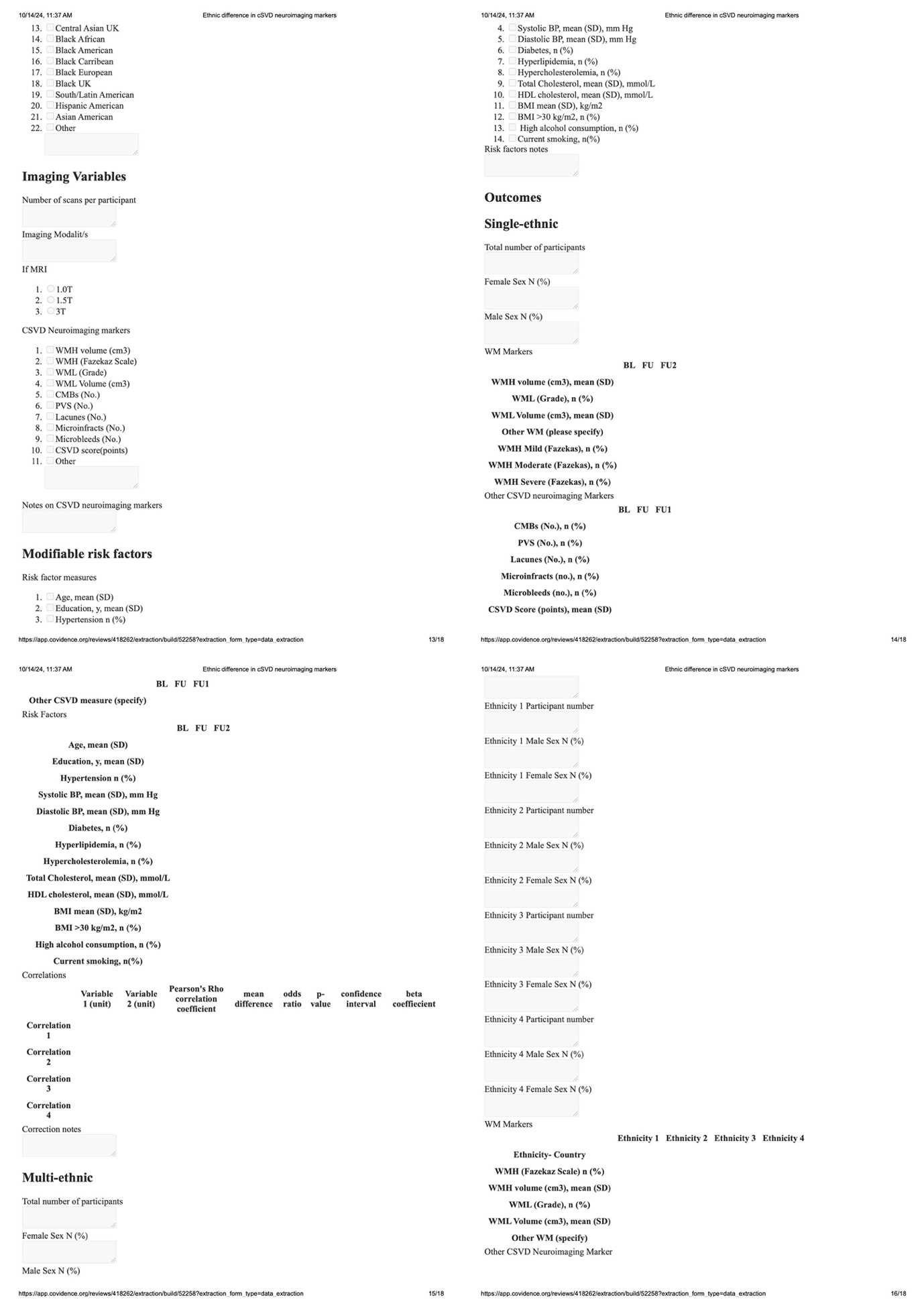


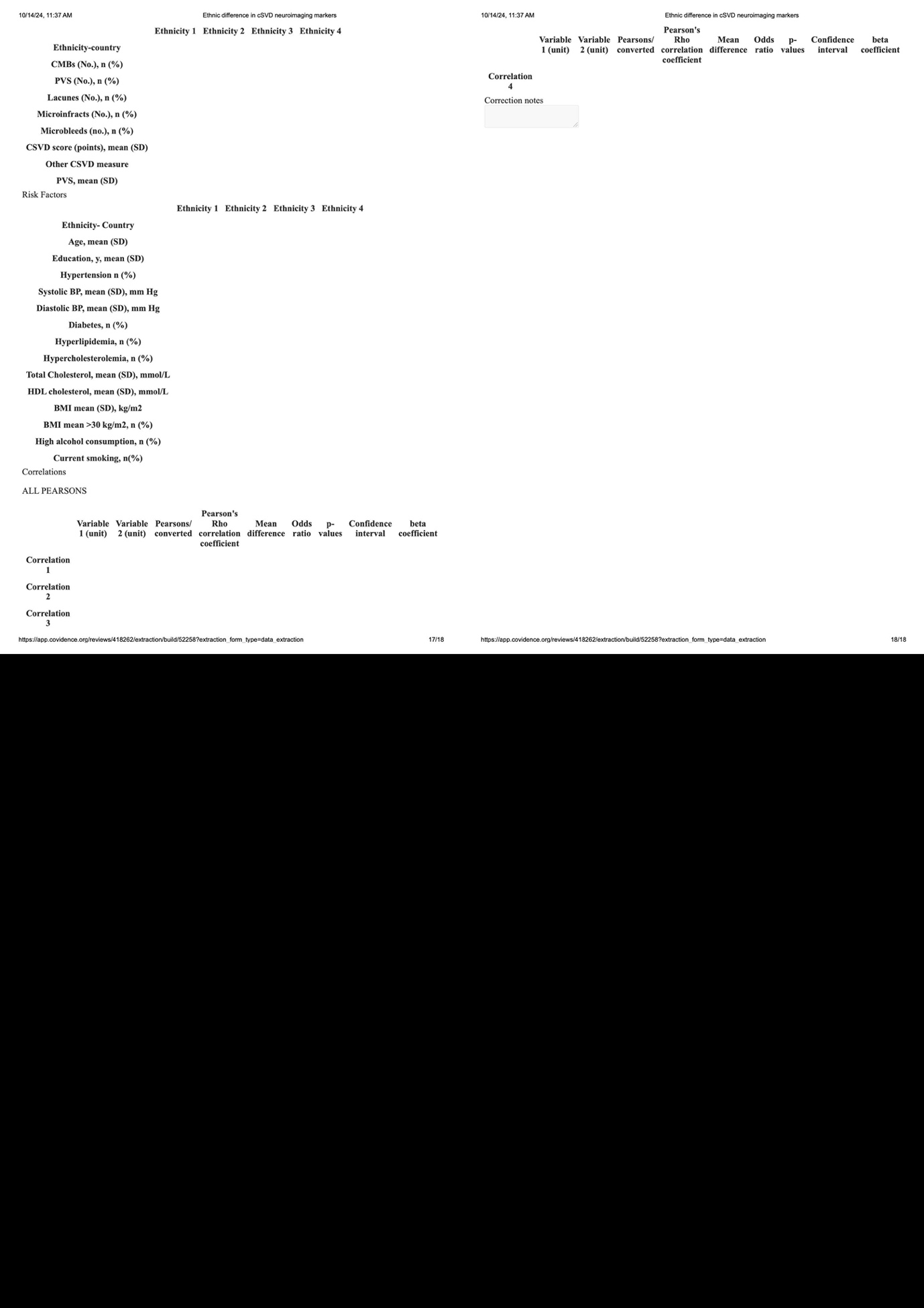


This Figure summarises the structure and main variables captured in the standardized data extraction form, including study identifiers, participant characteristics, CSVD markers (WMH, lacunes, microinfarcts, microbleeds), and associated vascular risk factors.

# **Supplementary Section 2. Risk of Bias Assessment- of Exposures (ROBANS-E) Criteria**

- **Selection Bias**: This domain considered the selection of participants, specifically whether there was any waiting period between the MRI scan and the collection of risk factors during both baseline (BL) and follow-up (FU) phases.
- **Confounding Factors**: The assessment examined if the studies adequately controlled for confounding variables such as age, living conditions/rural areas, access to healthcare, and medication use. It also evaluated whether these factors were measured accurately and whether there were differences in methodology between the baseline and follow-up assessments.
- **Exposure Measurement**: This domain reviewed whether the methodology for measuring risk factors was explicitly mentioned in the studies and whether it adhered to current standards.
- **Blinding of Outcome Assessment**: Particular attention was given to the blinding process in studies where neuroimaging markers were assessed manually, evaluating if the outcome assessments were performed in a blinded manner.
- **Incomplete Outcome Data**: The evaluation considered whether findings for both neuroimaging markers and risk factors were available for all participants, and if the availability of data was even addressed. This also included an assessment of the handling of incomplete data, specifically whether imputation methods were performed accurately, with attention to outcome, exposure, and confound variables.
- **Selective Outcome Reporting**: This domain investigated whether the studies followed a predetermined analysis plan and if the reported estimates were selected based on desirability. It also assessed if the selection of cohorts was derived from multiple analyses or statistical outcomes, raising potential concerns about selective reporting practices.

# **Supplementary Table 5. ROBANS-E Quality Assessment for included studies**

| **Study ID** | **Title** | **Selection of participants** | **Confounding factors** | **Exposure measurement adequate** | **Blinding of outcome assessment** | **Incomplete outcome data** | **Selective outcome reporting** |
| --- | --- | --- | --- | --- | --- | --- | --- |
| Wong 2019 | Prevalence and Risk Factors for Cognitive Impairment and Dementia in Indians: A Multiethnic Perspective from a Singaporean Study | High Quality | High Quality | High Quality | Low Quality | High Quality | Unclear |
| Takahashi 2006 | Multifractal analysis of deep white matter microstructural changes on MRI in relation to early-stage atherosclerosis | High Quality | Low Quality | Low Quality | High Quality | Low Quality | Unclear |
| Choi 2009 | Cerebral white matter hyperintensity is mainly associated with hypertension among the components of metabolic syndrome in Koreans | High Quality | High Quality | Low Quality | Low Quality | Low Quality | Low Quality |
| Ochi 2009 | Silent cerebral microbleeds associated with arterial stiffness in an apparently healthy subject | High Quality | High Quality | High Quality | Low Quality | Low Quality | Low Quality |
| Kim 2012 | Association of obesity with cerebral microbleeds in neurologically asymptomatic elderly subjects | High Quality | Unclear | High Quality | Low Quality | Low Quality | Low Quality |
| Choi 2013 | Arterial Stiffness Using Cardio-Ankle Vascular Index Reflects Cerebral Small Vessel Disease in Healthy Young and Middle Aged Subjects | Low Quality | High Quality | High Quality | High Quality | Low Quality | Low Quality |
| Park 2013 | Different impact of hyperhomocysteinemia on cerebral small vessel ischemia and cervico-cerebral atherosclerosis in non-stroke individuals | High Quality | High Quality | High Quality | Low Quality | High Quality | Unclear |
| Schilling 2014 | Plasma lipids and cerebral small vessel disease | High Quality | High Quality | High Quality | Low Quality | High Quality | High Quality |
| Lee 2015 | Association between Serum Alkaline Phosphatase Level and Cerebral Small Vessel Disease | High Quality | High Quality | High Quality | High Quality | High Quality | High Quality |
| Tabara 2015 | Association of Postural Instability With Asymptomatic Cerebrovascular Damage and Cognitive Decline The Japan Shimanami Health Promoting Program Study | High Quality | High Quality | High Quality | Low Quality | Low Quality | Low Quality |
| Kim 2015 | Relationship between Cerebral Microbleeds and Liver Stiffness Determined by Transient Elastography | High Quality | High Quality | High Quality | High Quality | Low Quality | Unclear |
| Honda 2015 | Volumetric analyses of cerebral white matter hyperintensity lesions on magnetic resonance imaging in a Japanese population undergoing medical check-up | High Quality | Low Quality | High Quality | Low Quality | Low Quality | Low Quality |
| Hayashi 2017 | Association of Kidney Dysfunction With Asymptomatic Cerebrovascular Abnormalities in a Japanese Population With Health Checkups | High Quality | High Quality | High Quality | Low Quality | High Quality | Unclear |
| Yang 2018 | Association of elevated blood pressure during exercise with cerebral white matter lesions | High Quality | Low Quality | High Quality | High Quality | Low Quality | Unclear |
| Rojas 2018 | Higher prevalence of cerebral white matter hyperintensities in homozygous <i>APOE-</i>Œµ<i>4</i> allele carriers aged 45-75: Results from the ALFA study | High Quality | High Quality | High Quality | High Quality | Low Quality | Unclear |
| Yang 2019 | Insulin Resistance Is a Risk Factor for Overall Cerebral Small Vessel Disease Burden in Old Nondiabetic Healthy Adult Population | High Quality | Low Quality | High Quality | Low Quality | Low Quality | Low Quality |
| Pettigrew 2020 | Cognitive reserve and rate of change in Alzheimer's and cerebrovascular disease biomarkers among cognitively normal individuals | High Quality | High Quality | High Quality | Low Quality | Low Quality | Low Quality |
| Lee 2020 | Echocardiographic index E/e' in association with cerebral white matter hyperintensity progression | High Quality | Low Quality | High Quality | High Quality | Low Quality | Low Quality |
| Wu 2020 | Insulin Resistance Is Independently Associated With Enlarged Perivascular Space in the Basal Ganglia in Nondiabetic Healthy Elderly Population | High Quality | Low Quality | High Quality | High Quality | Low Quality | Unclear |
| Hada 2020 | Periodontal Condition Is Correlated with Deep and Subcortical White Matter Hyperintensity Lesions in Japanese Adults | Unclear | Low Quality | High Quality | Low Quality | Low Quality | Unclear |
| Kim 2020 | Reduced forced vital capacity is associated with cerebral small vessel disease burden in cognitively normal individuals | High Quality | Unclear | High Quality | High Quality | High Quality | Unclear |
| Tamura 2021 | A 10-year longitudinal study of deep white matter lesions on magnetic resonance imaging | High Quality | Low Quality | High Quality | High Quality | Low Quality | Unclear |
| Lee 2021 | Effect of obstructive sleep apnea on cerebrovascular compliance and cerebral small vessel disease | High Quality | Low Quality | High Quality | High Quality | Unclear | Unclear |
| Jeong 2022 | Annual exposure to PM<sub>10</sub> is related to cerebral small vessel disease in general adult population | High Quality | High Quality | High Quality | Low Quality | Low Quality | Low Quality |
| Kuriyama 2022 | Association Between Cerebral Microbleeds and Circulating Levels of Mid-Regional Pro-Adrenomedullin | High Quality | High Quality | High Quality | High Quality | Low Quality | Unclear |
| Cai 2022 | Different mechanisms in periventricular and deep white matter hyperintensities in old subjects | Low Quality | Low Quality | Low Quality | High Quality | Low Quality | Low Quality |
| Liu 2022 | Excessive Visit-to-Visit Small and Dense Low-Density Lipoproteins Elevate Cerebral Small Vessel Disease Progression Risk in the Elderly | High Quality | High Quality | High Quality | High Quality | Low Quality | Unclear |
| Yamasaki 2022 | Factors associated with the location of perivascular space enlargement in middle-aged individuals undergoing brain screening in Japan | High Quality | Low Quality | High Quality | Low Quality | High Quality | Unclear |
| DelBrutto 2022 | Patterns of progression of cerebral small vessel disease markers in older adults of Amerindian ancestry: a population-based, longitudinal prospective cohort study | High Quality | High Quality | High Quality | High Quality | High Quality | Unclear |
| Park 2023 | Classification of white matter lesions and characteristics of small vessel disease markers | High Quality | High Quality | High Quality | High Quality | Low Quality | Unclear |
| Coomans 2023 | Interactions between vascular burden and amyloid-Œ≤ pathology on trajectories of tau accumulation | High Quality | Unclear | High Quality | Low Quality | High Quality | High Quality |
| Kartau 2023 | Neurofilament light level correlates with brain atrophy, and cognitive and motor performance | High Quality | Unclear | High Quality | High Quality | Low Quality | Unclear |
| Royse 2023 | Unhealthy white matter connectivity, cognition, and racialization in older adults | High Quality | Low Quality | High Quality | Low Quality | High Quality | Unclear |
| Shibata 2004 | Correlation of NO metabolites and 8-iso-prostaglandin F<sub>2a</sub> with periventricular hyperintensity severity | High Quality | Low Quality | High Quality | Low Quality | Low Quality | Low Quality |
| Fornage 2008 | Biomarkers of inflammation and MRI-defined small vessel disease of the brain - The Cardiovascular Health Study | High Quality | Low Quality | High Quality | High Quality | Low Quality | Low Quality |
| Wada 2008 | Cerebral small vessel disease and C-reactive protein: Results of a cross-sectional study in community-based Japanese elderly | High Quality | Low Quality | High Quality | High Quality | Low Quality | Unclear |
| Otani 2010 | Association of Kidney Dysfunction with Silent Lacunar Infarcts and White Matter Hyperintensity in the General Population: The Ohasama Study | High Quality | High Quality | Low Quality | Low Quality | Low Quality | Low Quality |
| Kuo 2010 | Metabolic risks, white matter hyperintensities, and arterial stiffness in high-functioning healthy adults | High Quality | High Quality | High Quality | Low Quality | Low Quality | Low Quality |
| Kim 2011 | Advanced Coronary Artery Calcification and Cerebral Small Vessel Diseases in the Healthy Elderly | High Quality | High Quality | High Quality | Low Quality | High Quality | Unclear |
| Kim 2011 | Age-independent association of pulse pressure with cerebral white matter lesions in asymptomatic elderly individuals | High Quality | Unclear | High Quality | Low Quality | Low Quality | Low Quality |
| Yao 2011 | Chronic kidney disease and subclinical lacunar infarction are independently associated with frontal lobe dysfunction in community-dwelling elderly subjects: the Sefuri brain MRI study | High Quality | High Quality | High Quality | High Quality |  |  |
| Takahashi 2011 | Impaired Glucose Metabolism Slows Executive Function Independent of Cerebral Ischemic Lesions in Japanese Elderly: The Takahata Study | High Quality | High Quality | High Quality | High Quality | High Quality | High Quality |
| Elnimr 2012 | Association between white matter hyperintensity and lacunar infarction on MRI and subitem scores of the Japanese version of mini-mental state examination for testing cognitive decline: the Ohasama study | High Quality | High Quality | High Quality | High Quality | High Quality | High Quality |
| Nakano 2012 | Augmentation index is related to white matter lesions | High Quality | Low Quality | High Quality | Low Quality | Low Quality | Low Quality |
| Takahashi 2012 | Relationship Between Chronic Kidney Disease and White Matter Hyperintensities on Magnetic Resonance Imaging | High Quality | High Quality | High Quality | Low Quality | Low Quality | Low Quality |
| Taguchi 2013 | Association between oral health and the risk of lacunar infarction in Japanese adults | High Quality | Low Quality | High Quality | High Quality | Low Quality | Low Quality |
| Brisset 2013 | Large-vessel correlates of cerebral small-vessel disease | High Quality | High Quality | High Quality | Low Quality | Low Quality | Low Quality |
| Kim 2014 | Circulating Matrix Metalloproteinase-9 Level Is Associated with Cerebral White Matter Hyperintensities in Non-Stroke Individuals | High Quality | High Quality | High Quality | Low Quality | Low Quality | Low Quality |
| Hashimoto 2014 | Dual Task Walking Reveals Cognitive Dysfunction in Community-dwelling Elderly Subjects: The Sefuri Brain MRI Study | Low Quality | Unclear | High Quality | Low Quality | Low Quality | Low Quality |
| Yamaguchi 2014 | Impact of Ambulatory Blood Pressure Variability on Cerebral Small Vessel Disease Progression and Cognitive Decline in Community-Based Elderly Japanese | High Quality | Low Quality | High Quality | High Quality | High Quality | High Quality |
| Aarts 2014 | Inhibition of Serotonin Reuptake by Antidepressants and Cerebral Microbleeds in the General Population | High Quality | High Quality | High Quality | High Quality | High Quality | High Quality |
| Duering 2014 | Strategic white matter tracts for processing speed deficits in age-related small vessel disease | High Quality |  | Low Quality | Low Quality | High Quality | Unclear |
| Yakushiji 2014 | Topography and associations of perivascular spaces in healthy adults The Kashima Scan Study | High Quality | High Quality | High Quality | High Quality | Low Quality | Unclear |
| Soriano-Raya 2014 | Tract-specific fractional anisotropy predicts cognitive outcome in a community sample of middle-aged participants with white matter lesions | High Quality | Low Quality | High Quality | Low Quality | Low Quality | Low Quality |
| Yamashiro 2014 | Visceral fat accumulation is associated with cerebral small vessel disease | High Quality | High Quality | High Quality | Low Quality | Low Quality | Low Quality |
| Toyoda 2015 | Association of Mild Kidney Dysfunction with Silent Brain Lesions in Neurologically Normal Subjects | High Quality | Low Quality | Low Quality | Low Quality | Low Quality | Low Quality |
| Gustavsson 2015 | Cerebral Microbleeds and White Matter Hyperintensities in Cognitively Healthy Elderly: A Cross-Sectional Cohort Study Evaluating the Effect of Arterial Stiffness | High Quality | High Quality | High Quality | High Quality | High Quality | Unclear |
| Shinoda 2015 | Inverse Relationship between Coffee Consumption and Cerebral Microbleeds in Men, but Not Women | Low Quality | Low Quality | Low Quality | Low Quality | Low Quality | Low Quality |
| Power 2015 | Smoking and white matter hyperintensity progression The ARIC-MRI Study | High Quality | High Quality | High Quality | Low Quality | High Quality | Unclear |
| Kinjo 2016 | A high normal ankle-brachial index combined with a high pulse wave velocity is associated with cerebral microbleeds | High Quality | High Quality | High Quality | High Quality | Low Quality | Unclear |
| Cho 2016 | Albuminuria, Cerebrovascular Disease and Cortical Atrophy: among Cognitively Normal Elderly Individuals | High Quality | Low Quality | High Quality | High Quality | High Quality | High Quality |
| Mitaki 2016 | C-reactive protein levels are associated with cerebral small vessel-related lesions | High Quality | High Quality | High Quality | High Quality | High Quality | High Quality |
| Cooper 2016 | Cerebrovascular Damage Mediates Relations Between Aortic Stiffness and Memory | High Quality | High Quality | High Quality | Low Quality | High Quality | Unclear |
| Lee 2016 | Coronary artery calcium is associated with cortical thinning in cognitively normal individuals | High Quality | High Quality | High Quality | Low Quality | High Quality | Unclear |
| Park 2016 | Decreased hemoglobin levels, cerebral small-vessel disease, and cortical atrophy: among cognitively normal elderly women and men | High Quality | High Quality | High Quality | High Quality | High Quality | High Quality |
| Resende 2016 | Ischemic cerebrovascular burden evaluated by magnetic resonance imaging in an elderly Brazilian community: The Piet√ study | Low Quality | Low Quality | High Quality | High Quality | Low Quality | Low Quality |
| Kim 2017 | Kidney dysfunction and cerebral microbleeds in neurologically healthy adults | High Quality | High Quality | High Quality | Low Quality | High Quality | High Quality |
| Kim 2017 | Kidney dysfunction and silent brain infarction in generally healthy adults | High Quality | High Quality | High Quality | High Quality | High Quality | High Quality |
| Ding 2017 | Large Perivascular Spaces Visible on Magnetic Resonance Imaging, Cerebral Small Vessel Disease Progression, and Risk of Dementia The Age, Gene/Environment Susceptibility-Reykjavik Study | High Quality | Unclear | High Quality | Low Quality | High Quality | Unclear |
| Hilal 2017 | Prevalence, risk factors and consequences of cerebral small vessel diseases: data from three Asian countries | High Quality | High Quality | Low Quality | Unclear | Low Quality | Unclear |
| Rundek 2017 | Relationship between carotid arterial properties and cerebral white matter hyperintensities | High Quality | High Quality | High Quality | Low Quality | Low Quality | Low Quality |
| Mitaki 2017 | Serum Lipid Fractions and Cerebral Microbleeds in a Healthy Japanese Population | High Quality | Low Quality | High Quality | Low Quality | High Quality | Unclear |
| Higuchi 2017 | Visceral-to-subcutaneous fat ratio is independently related to small and large cerebrovascular lesions even in healthy subjects | Unclear | Low Quality | High Quality | Low Quality | Low Quality | Low Quality |
| Duperron 2018 | Burden of Dilated Perivascular Spaces, an Emerging Marker of Cerebral Small Vessel Disease, Is Highly Heritable | High Quality | High Quality | High Quality | Low Quality | Low Quality | Low Quality |
| Sudre 2018 | Cardiovascular Risk Factors and White Matter Hyperintensities: Difference in Susceptibility in South Asians Compared With Europeans | High Quality | Low Quality | High Quality | Low Quality | Low Quality | Low Quality |
| Caughey 2018 | Carotid Intima-Media Thickness and Silent Brain Infarctions in a Biracial Cohort: The Atherosclerosis Risk in Communities (ARIC) Study | High Quality | High Quality | High Quality | Low Quality | Low Quality | Low Quality |
| Ao 2018 | Large Vessel Disease Modifies the Relationship Between Kidney Injury and Cerebral Small Vessel Disease | High Quality | High Quality | High Quality | High Quality | High Quality | High Quality |
| DelBrutto 2018 | Neuroimaging Signatures of Cerebral Small Vessel Disease at Blood Pressure Cutoff Levels of 130/80 and 140/90 mmHg: A Population-Based Study in Community-Dwellers Aged ‚â• 60 Years | High Quality | Unclear | High Quality | High Quality | Low Quality | Low Quality |
| Silbert 2018 | Risk Factors Associated with Cortical Thickness and White Matter Hyperintensities in Dementia Free Okinawan Elderly | High Quality | Low Quality | High Quality | Low Quality | Low Quality | Low Quality |
| Yin 2018 | Sex differences in associations between blood lipids and cerebral small vessel disease | High Quality | High Quality | High Quality | High Quality | Low Quality | Unclear |
| Rizvi 2018 | The effect of white matter hyperintensities on cognition is mediated by cortical atrophy | High Quality | Low Quality | High Quality | Low Quality | High Quality | Unclear |
| Ding 2018 | White Matter Hyperintensity Predicts the Risk of Incident Cognitive Decline in Community Dwelling Elderly | High Quality | High Quality | High Quality | Low Quality | Low Quality | Low Quality |
| Kynast 2018 | White matter hyperintensities associated with small vessel disease impair social cognition beside attention and memory | High Quality | High Quality | High Quality | High Quality | High Quality | High Quality |
| Croll 2019 | Body Composition Is Not Related to Structural or Vascular Brain Changes | High Quality | Low Quality | High Quality | High Quality | Low Quality | Low Quality |
| Kang 2019 | Can transcranial Doppler ultrasound be used for screening cerebral small vessel diseases in the community? | High Quality | Unclear | High Quality | High Quality | High Quality | Unclear |
| Li 2019 | Incidental findings on brain MRI among Chinese at the age of 55-65 years: the Taizhou Imaging Study | High Quality | High Quality | High Quality | Low Quality | High Quality | Unclear |
| Kim 2019 | Kidney Dysfunction Impact on White Matter Hyperintensity Volume in Neurologically Healthy Adults | High Quality | High Quality | High Quality | High Quality | High Quality | High Quality |
| Chou 2019 | Location-Specific Association Between Cerebral Microbleeds and Arterial Pulsatility | High Quality | High Quality | High Quality | High Quality | Low Quality | Low Quality |
| Yamada 2019 | Location-specific characteristics of perivascular spaces as the brain's interstitial fluid drainage system | High Quality | High Quality | High Quality | Low Quality | High Quality | Unclear |
| Chen 2019 | Low carotid endothelial shear stress associated with cerebral small vessel disease in an older population: A subgroup analysis of a population-based prospective cohort study | High Quality | High Quality | High Quality | High Quality | Low Quality | Low Quality |
| Hara 2019 | Synergistic effect of hypertension and smoking on the total small vessel disease score in healthy individuals: the Kashima scan study | High Quality | High Quality | High Quality | High Quality | Low Quality | Low Quality |
| Vangberg 2019 | The effect of white matter hyperintensities on regional brain volumes and white matter microstructure, a population-based study in HUNT | High Quality | Low Quality | Low Quality | Low Quality | High Quality | Unclear |
| Jiang 2019 | Total Cerebral Small Vessel Disease Burden Is Related to Worse Performance on the Mini-Mental State Examination and Incident Dementia: A Prospective 5-Year Follow-Up | High Quality | High Quality | High Quality | High Quality | High Quality | Unclear |
| Lampe 2019 | Visceral obesity relates to deep white matter hyperintensities via inflammation | High Quality | High Quality | High Quality | Low Quality | High Quality | High Quality |
| Guo 2020 | Assessing the effectiveness of statin therapy for alleviating cerebral small vessel disease progression in people ‚â•75years of age | High Quality | High Quality | High Quality | High Quality | Low Quality | Low Quality |
| Kim 2020 | Association of Low Blood Pressure with White Matter Hyperintensities in Elderly Individuals with Controlled Hypertension | High Quality | High Quality | High Quality | Low Quality | Low Quality | Low Quality |
| Suchy-Dicey 2020 | Cognitive Correlates of MRI-defined Cerebral Vascular Injury and Atrophy in Elderly American Indians: The Strong Heart Study | High Quality | Low Quality | High Quality | High Quality | High Quality | High Quality |
| Sim 2020 | Correlation Between Hippocampal Enlarged Perivascular Spaces and Cognition in Non-dementic Elderly Population | High Quality | High Quality | High Quality | High Quality | Low Quality | Unclear |
| Biesbroek 2020 | High white matter hyperintensity burden in strategic white matter tracts relates to worse global cognitive performance in community-dwelling individuals | High Quality | High Quality | Low Quality | Unclear | Unclear | High Quality |
| Cui 2020 | Home-measured orthostatic hypotension associated with cerebral small vessel disease in a community-based older population | High Quality | High Quality | High Quality | High Quality | High Quality | High Quality |
| Boots 2020 | Inflammation, Cognition, and White Matter in Older Adults: An Examination by Race | High Quality | High Quality | High Quality | High Quality | Low Quality | Unclear |
| Zeng 2020 | Severity of white matter hyperintensities: Lesion patterns, cognition, and microstructural changes | High Quality | Low Quality | High Quality | High Quality | Low Quality | Unclear |
| Wang 2020 | Strictly Lobar Cerebral Microbleeds Are Associated with Increased White Matter Volume | High Quality | High Quality | High Quality | High Quality | Low Quality | Unclear |
| Peng 2020 | Structural and Microvascular Changes in the Macular Are Associated With Severity of White Matter Lesions | High Quality | High Quality | High Quality | High Quality | Low Quality | Unclear |
| Garnier-Crussard 2020 | White matter hyperintensities across the adult lifespan: relation to age, AŒ≤ load, and cognition | High Quality | Low Quality | High Quality | Low Quality | High Quality | Low Quality |
| Xia 2020 | White matter hyperintensities associated with progression of cerebral small vessel disease: a 7-year Chinese urban community study | High Quality | High Quality | High Quality | High Quality | High Quality | High Quality |
| Gronewold 2021 | Association of Blood Pressure, Its Treatment, and Treatment Efficacy With Volume of White Matter Hyperintensities in the Population-Based 1000BRAINS Study | High Quality | High Quality | High Quality | High Quality | High Quality | High Quality |
| Zhang 2021 | Association of Carotid Atherosclerosis With White Matter Hyperintensity in an Asymptomatic Japanese Population: A Cross-Sectional Study | High Quality | High Quality | High Quality | High Quality | Low Quality | Unclear |
| Chuang 2021 | Associations of Blood Pressure and Carotid Flow Velocity with Brain Volume and Cerebral Small Vessel Disease in a Community-Based Population | High Quality | High Quality | High Quality | High Quality | Unclear | Unclear |
| Hotz 2021 | Associations of subclinical cerebral small vessel disease and processing speed in non-demented subjects: A 7-year study | High Quality | High Quality | High Quality | Low Quality | High Quality | Unclear |
| Ryd√©n 2021 | Atrial Fibrillation, Stroke, and Silent Cerebrovascular Disease A Population-based MRI Study | High Quality | High Quality | High Quality | High Quality | High Quality | High Quality |
| Ao 2021 | Brain deep medullary veins on 3-T MRI in a population-based cohort | High Quality | High Quality | High Quality | Low Quality | High Quality | High Quality |
| Buhrmann 2021 | Cerebellar Grey Matter Volume in Older Persons Is Associated with Worse Cognitive Functioning | High Quality | Low Quality | High Quality | High Quality | Low Quality | Low Quality |
| Heiland 2021 | Cerebral small vessel disease, cardiovascular risk factors, and future walking speed in old age: a population-based cohort study | High Quality | High Quality | High Quality | Low Quality | High Quality | Unclear |
| Pahlavian 2021 | Cerebroarterial pulsatility and resistivity indices are associated with cognitive impairment and white matter hyperintensity in elderly subjects: A phase-contrast MRI study | High Quality | Low Quality | High Quality | High Quality | High Quality | High Quality |
| Cao 2021 | Correlation between total homocysteine and cerebral small vessel disease: A Mendelian randomization study | High Quality | Low Quality | High Quality | High Quality | High Quality | Unclear |
| Huang 2021 | Deep white matter hyperintensity is associated with the dilation of perivascular space | High Quality | Low Quality | High Quality | High Quality | Low Quality | Low Quality |
| Jann 2021 | Evaluation of Cerebral Blood Flow Measured by 3D PCASL as Biomarker of Vascular Cognitive Impairment and Dementia (VCID) in a Cohort of Elderly Latinx Subjects at Risk of Small Vessel Disease | High Quality | High Quality | High Quality | Low Quality | Low Quality | Unclear |
| Vergoossen 2021 | Interplay of White Matter Hyperintensities, Cerebral Networks, and Cognitive Function in an Adult Population: Diffusion-Tensor Imaging in the Maastricht Study | High Quality | Low Quality | High Quality | Low Quality | High Quality | Unclear |
| Vintimilla 2021 | MRI biomarkers of small vessel disease and cognition: A cross-sectional study of a cognitively normal Mexican American cohort | High Quality | High Quality | High Quality | Low Quality | Low Quality | Low Quality |
| Hao 2021 | Natural history of silent lacunar infarction: 10-year follow-up of a community-based prospective study of 0.5 million Chinese adults | High Quality | Unclear | High Quality | High Quality | Low Quality | Low Quality |
| Wang 2021 | Quantity and Morphology of Perivascular Spaces: Associations With Vascular Risk Factors and Cerebral Small Vessel Disease | High Quality | Low Quality | High Quality | Low Quality | Low Quality | Low Quality |
| Qi 2021 | Relationship of Visceral Adipose Tissue With Dilated Perivascular Spaces | High Quality | Low Quality | High Quality | High Quality | Low Quality | Low Quality |
| Jack√≥w-Nowicka 2021 | The Impact of Common Epidemiological Factors on Gray and White Matter Volumes in Magnetic Resonance Imaging-Is Prevention of Brain Degeneration Possible? | High Quality | Low Quality | High Quality | Low Quality | High Quality | Unclear |
| Luo 2021 | The Prevalence and Risk Factors of Cerebral Microbleeds: A Community-Based Study in China | High Quality | High Quality | High Quality | Low Quality | Low Quality | Low Quality |
| Frey 2021 | White matter integrity and structural brain network topology in cerebral small vessel disease: The Hamburg city health study | High Quality | Unclear | High Quality | Low Quality | High Quality | Unclear |
| Sudre 2022 | Accelerated vascular aging: Ethnic differences in basilar artery length and diameter, and its association with cardiovascular risk factors and cerebral small vessel disease | High Quality | High Quality | High Quality | Low Quality | High Quality | Unclear |
| Brutto 2022 | Arterial stiffness and progression of white matter hyperintensities of presumed vascular origin in community-dwelling older adults of Amerindian ancestry: The Atahualpa Project Cohort | High Quality | High Quality | High Quality | High Quality | Low Quality | High Quality |
| Zhao 2022 | Association Between Intracranial Pulsatility and White Matter Hyperintensities in Asymptomatic Intracranial Arterial Stenosis: A Population-Based Study in Shandong, China | High Quality | Low Quality | High Quality | High Quality | High Quality | High Quality |
| Yu 2022 | Association between haemoglobin A1c and cerebral microbleeds in community-based stroke-free individuals: A cross-sectional study | High Quality | High Quality | High Quality | High Quality | Low Quality | Unclear |
| Rimmele 2022 | Association of Carotid Plaque and Flow Velocity With White Matter Integrity in a Middle-aged to Elderly Population | High Quality | Unclear | High Quality | Low Quality | High Quality | Unclear |
| Choi 2022 | Association of cerebral white matter hyperintensities with coronary artery calcium in a healthy population: a cross-sectional study | High Quality | High Quality | High Quality | High Quality | High Quality | High Quality |
| Han 2022 | Associations between cerebral blood flow and progression of white matter hyperintensity in community-dwelling adults: a longitudinal cohort study | High Quality | Low Quality | High Quality | High Quality | High Quality | Unclear |
| Yoshiura 2022 | Brain structural alterations and clinical features of cognitive frailty in Japanese community-dwelling older adults: the Arao study (JPSC-AD) | High Quality | High Quality | High Quality | Low Quality | High Quality | High Quality |
| Ghazali 2022 | Presence of enlarged perivascular spaces is associated with reduced processing speed in asymptomatic, working-aged adults | Unclear | Low Quality | High Quality | High Quality | Low Quality | Low Quality |
| Mu 2022 | Prevalence and Consequences of Cerebral Small Vessel Diseases: A Cross-Sectional Study Based on Community People Plotted Against 5-Year Age Strata | High Quality | Unclear | High Quality | High Quality | Low Quality | Unclear |
| Wu 2022 | Prevalence and risk factors of MRI-defined brain infarcts among Chinese adults | High Quality | Low Quality | High Quality | High Quality | High Quality | High Quality |
| Lohner 2022 | Relation Between Sex, Menopause, and White Matter Hyperintensities The Rhineland Study | High Quality | High Quality | High Quality | Low Quality | High Quality | Unclear |
| Jung 2022 | Relationship of the Triglyceride-Glucose Index with Subclinical White Matter Hypersensitivities of Presumed Vascular Origin Among Community-Dwelling Koreans | High Quality | High Quality |  | High Quality | Low Quality | Unclear |
| Tao 2022 | Retinal microvasculature and imaging markers of brain frailty in normal aging adults | High Quality | High Quality | High Quality | High Quality | Low Quality | Unclear |
| Bonberg 2022 | Sex-Specific Causes and Consequences of White Matter Damage in a Middle-Aged Cohort | High Quality | High Quality | High Quality | Unclear | High Quality | High Quality |
| Melgarejo 2022 | Subclinical Magnetic Resonance Imaging Markers of Cerebral Small Vessel Disease in Relation to Office and Ambulatory Blood Pressure Measurements | High Quality | High Quality | High Quality | Low Quality | High Quality | Unclear |
| Chu 2022 | Subclinical hypothyroidism is associated with basal ganglia enlarged perivascular spaces and overall cerebral small vessel disease load | High Quality | Low Quality | High Quality | High Quality | Low Quality | Unclear |
| Duchesne 2023 | Ambient Air Pollution Exposure and Cerebral White Matter Hyperintensities in Older Adults: A Cross-Sectional Analysis in the Three-City Montpellier Study | High Quality | High Quality | High Quality | Low Quality | High Quality | High Quality |
| Mayer 2023 | Association between Coffee Consumption and Brain MRI Parameters in the Hamburg City Health Study | High Quality | Unclear | High Quality | Low Quality | High Quality | Unclear |
| Li 2023 | Association of blood pressure with brain perfusion and structure: A population-based prospective study | High Quality | High Quality | High Quality | Low Quality | High Quality | Unclear |
| Cai 2023 | Association of intracranial atherosclerosis with cerebral small vessel disease in a community-based population | High Quality | High Quality | High Quality | High Quality | High Quality | Unclear |
| Li 2023 | Association of white matter hyperintensity accumulation with domain-specific cognitive decline: a population-based cohort study | High Quality | Low Quality | High Quality | Low Quality | Low Quality | Low Quality |
| Nakazawa 2023 | Association of white matter lesions and brain atrophy with the development of dementia in a community: the Hisayama Study | High Quality | High Quality | High Quality | High Quality | Low Quality | Unclear |
| Hotz 2023 | Associations between white matter hyperintensities, lacunes, entorhinal cortex thickness, declarative memory and leisure activity in cognitively healthy older adults: A 7-year study | High Quality | High Quality | High Quality | Low Quality | High Quality | Unclear |
| Jiang 2023 | Associations of Microvascular Dysfunction with Mild Cognitive Impairment and Cognitive Function Among Rural-Dwelling Older Adults in China | High Quality | Low Quality | High Quality | High Quality | High Quality | Unclear |
| Cho 2023 | Associations of Particulate Matter Exposures With Brain Gray Matter Thickness and White Matter Hyperintensities: Effect Modification by Low-Grade Chronic Inflammation | High Quality | High Quality | High Quality | Low Quality | High Quality | High Quality |
| Ellstr√∂m 2023 | Associations of carotid artery flow parameters with MRI markers of cerebral small vessel disease and patterns of brain atrophy | High Quality | Low Quality | High Quality | Low Quality | Low Quality | Low Quality |
| Drenth 2023 | Functional connectivity in older adults-the effect of cerebral small vessel disease | High Quality | Low Quality | High Quality | Low Quality | High Quality | High Quality |
| Hou 2023 | Impact of Cerebral Microbleeds on Gait, Balance, and Upper Extremities Function in Cerebral Small Vessel Disease | High Quality | High Quality | High Quality | High Quality | Low Quality | Low Quality |
| Wang 2023 | Retinal ganglion cell-inner plexiform layer, white matter hyperintensities, and their interaction with cognition in older adults | High Quality | Unclear | High Quality | High Quality | Low Quality | Unclear |
| Zhou 2023 | Type A personality, sleep quality, and cerebral small vessel disease: investigating the mediating role of sleep in a community-based study | High Quality | Low Quality | High Quality | Low Quality | Low Quality | Low Quality |
| Sun 2024 | Association of variability in body size with neuroimaging metrics of brain health: a population-based cohort study | High Quality | Unclear | High Quality | High Quality | High Quality | High Quality |
| Tachibana 2024 | Late-Life High Blood Pressure and Enlarged Perivascular Spaces in the Putaminal Regions of Community-Dwelling Japanese Older Persons | High Quality | High Quality | High Quality | Low Quality | High Quality | Unclear |
| Hu 2025 | Peak width of skeletonized mean diffusivity mediates the relationship between cerebral small vessel disease burden and cognitive impairment in community-dwelling older adults | High Quality | High Quality | High Quality | High Quality | Unclear | Unclear |
| Zhang 2025 | Cerebral small vessel disease among rural-dwelling Chinese older adults: prevalence, distribution, and associated factors | High Quality | High Quality | High Quality | High Quality | High Quality | High Quality |
| Yang 2025 | Association of Left Ventricular Function With Cerebral Small Vessel Disease in a Community-Based Population | High Quality | High Quality | High Quality | High Quality | High Quality | High Quality |
| Cai 2025 | Carotid artery atherosclerosis and white matter lesions in the elderly: a community-based MR imaging study | High Quality | High Quality | High Quality | High Quality | High Quality | High Quality |
| Ariko 2024 | Estimated Pulse-Wave Velocity and Magnetic Resonance Imaging Markers of Cerebral Small-Vessel Disease in the NOMAS | High Quality | High Quality | High Quality | Unclear | High Quality | High Quality |
| Wang 2024 | Association between MTHFR C677T polymorphism and cognitive impairment in patients with cerebral small vessel disease: a cross-sectional study | High Quality | High Quality | Low Quality | Low Quality | High Quality | Unclear |
| Lv 2024 | Associations of serum uric acid variability with neuroimaging metrics and cognitive decline: a population-based cohort study | High Quality | High Quality | High Quality | High Quality | Low Quality | Low Quality |
| Ackah 2025 | The burden of intracranial atherosclerosis on cerebral small vessel disease: A community cohort study | Unclear | Low Quality | Unclear | Unclear | High Quality | Low Quality |
| Vikner 2025 | MRI contrast accumulation in features of cerebral small vessel disease: blood-brain barrier dysfunction or elevated vascular density? | Low Quality | High Quality | High Quality | Low Quality | High Quality | Unclear |
| Cao 2025 | Low Qualityer Retinal Arteriolar Density Is Associated With High Qualityer Cerebral Small Vessel Disease Burden: An Optical Coherence Tomography Angiography Study | High Quality | High Quality | High Quality | High Quality | Unclear | Unclear |
| Ishida 2025 | Association of Obesity and Metabolic Health Status with Cerebral Small-Vessel Disease in Stroke-Free Individuals | High Quality | High Quality | High Quality | High Quality | Unclear | Unclear |
| Ma 2025 | Correlation Between Blood Glucose Level and Cerebral Small Vessel Disease Markers in Neurologically Asymptomatic, Nondiabetic Individuals | Low Quality | High Quality | Low Quality | High Quality | Unclear | Unclear |
| Cui 2024 | Small vessel disease and cognitive reserve oppositely modulate global network redundancy and cognitive function: A study in middle-to-old aged community participants | High Quality | High Quality | Unclear | High Quality | Unclear | Unclear |
| Jokinen 2025 | Executive functions and processing speed in covert cerebral small vessel disease | High Quality | High Quality | High Quality | Low Quality | Unclear | Unclear |
| Yohe 2025 | Predictors of white matter hyperintensities in the elderly Congolese population | Unclear | Low Quality | Low Quality | Low Quality | Low Quality | High Quality |
| Xu 2024 | Analysis of gait pattern related to high cerebral small vessel disease burden using quantitative gait data from wearable sensors | High Quality | High Quality | High Quality | Low Quality | Unclear | Unclear |
| Sugai 2024 | Augmentation of perivascular space visualization in basal ganglia and white matter hyperintensity lesion is a meaningful finding for subsequent cognitive decline | High Quality | High Quality | High Quality | High Quality | High Quality | High Quality |
| Siddiquee 2025 | Middle-age cerebral small vessel disease and cognitive function in later life: a population-based prospective cohort study | High Quality | Low Quality | High Quality | High Quality | Unclear | Unclear |
| Okada 2025 | Morphologic variation of the circle of Willis is associated with cognitive impairment in a general population: The J-SHIPP study | High Quality | Low Quality | Low Quality | Low Quality | Unclear | Unclear |
| Jali 2024 | Prevalence and Risk Factors of Cerebral Microbleeds in Community-Dwelling Adults in Urban Delhi | High Quality | High Quality | High Quality | High Quality | High Quality | High Quality |
| Courtney 2025 | Connecting the dots: microstructural properties of white matter hyperintensities predict longitudinal cognitive changes in ageing | High Quality | High Quality | High Quality | Low Quality | High Quality | Unclear |

# **Supplementary Figure 2. CSVD- Publication Bias Funnel Plots**


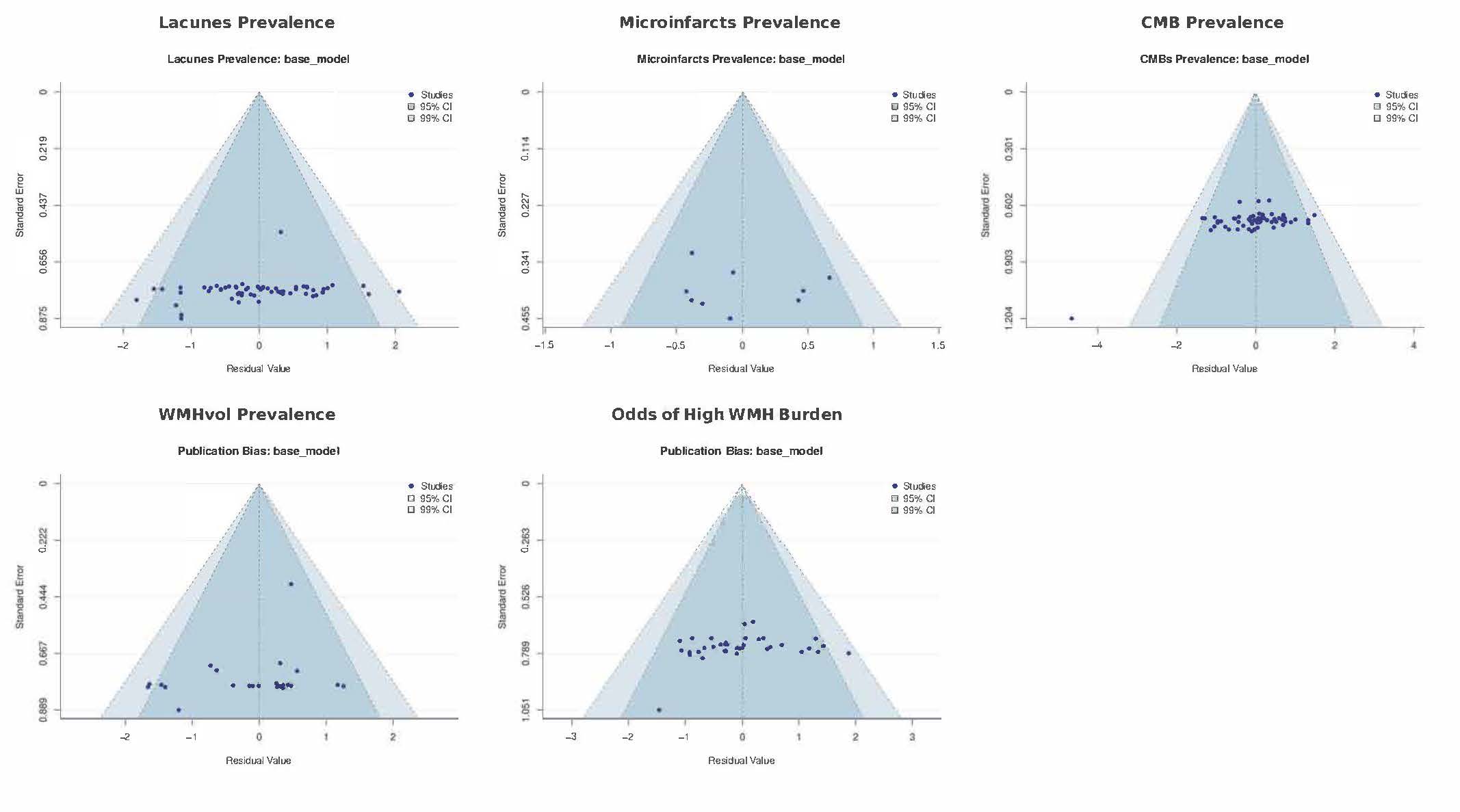


This figure presents a visual comparison of base model publication bias funnel plots for CSVD outcomes. It includes WMH volume (WMHVOL), WMH Fazekas high odds, lacunes, microinfarcts, and cerebral microbleeds (CMBs). Each plot illustrates the distribution of studies, highlighting potential publication bias with shaded regions representing 95% and 99% confidence intervals. A sixth placeholder is included for visual balance in the 2x3 grid layout.

# **Supplementary Figure 3. Prevalence Risk Factor- Publication Bias Funnel Plots**


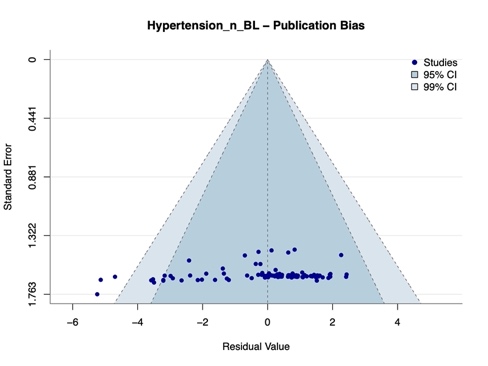

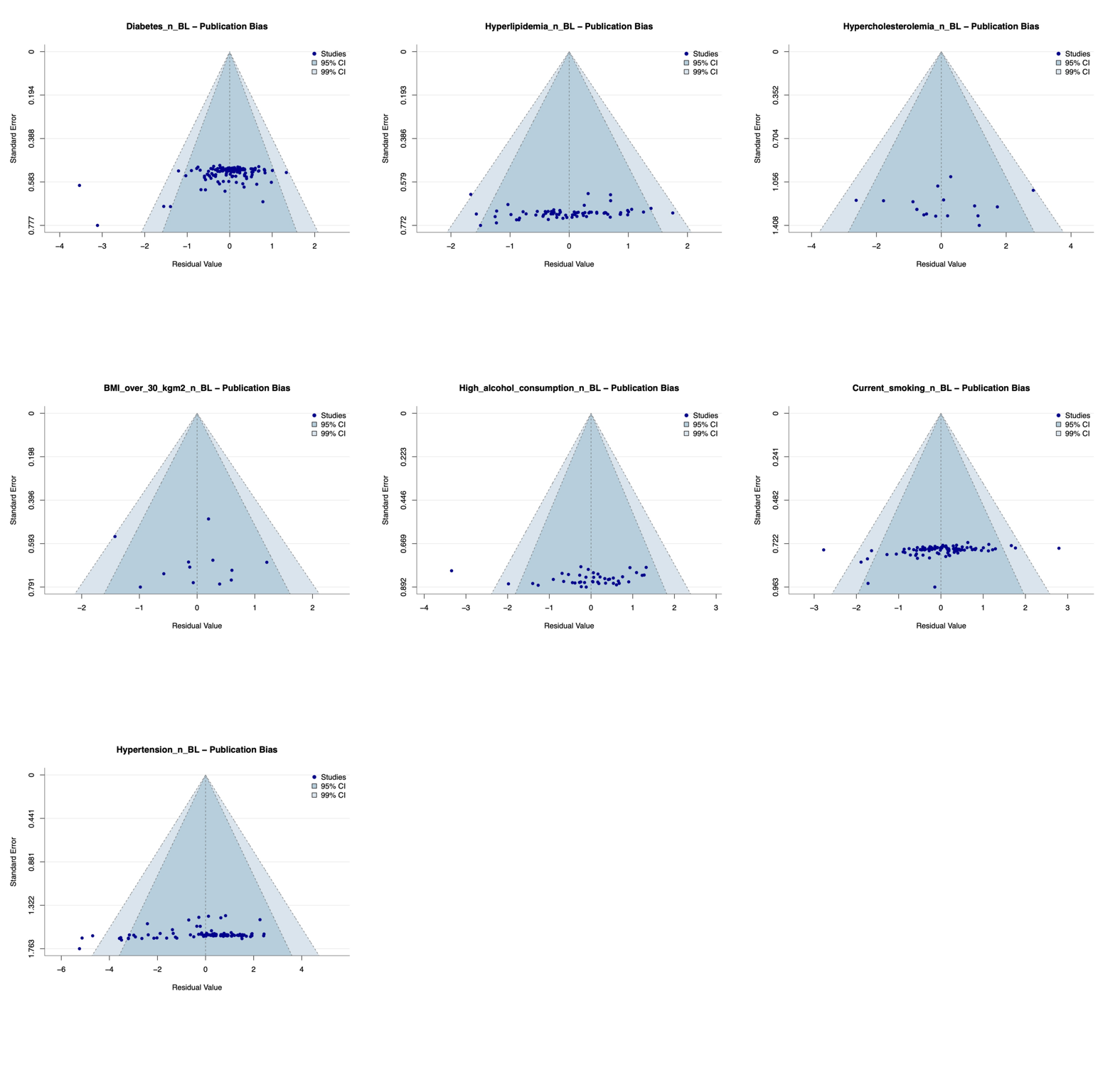


This figure presents a visual comparison of publication bias funnel plots for various risk factor prevalences. It includes diabetes, hyperlipidemia, hypercholesterolemia, obesity (BMI over 30 kg/m²), high alcohol consumption, current smoking, and hypertension. Each plot illustrates the distribution of studies, highlighting potential publication bias with shaded regions representing 95% and 99% confidence intervals. The 3x2+1 grid layout is used to provide a balanced visual presentation of the data.

# **Supplementary Figure 4. Continuous Risk Factor- Publication Bias Funnel Plots**


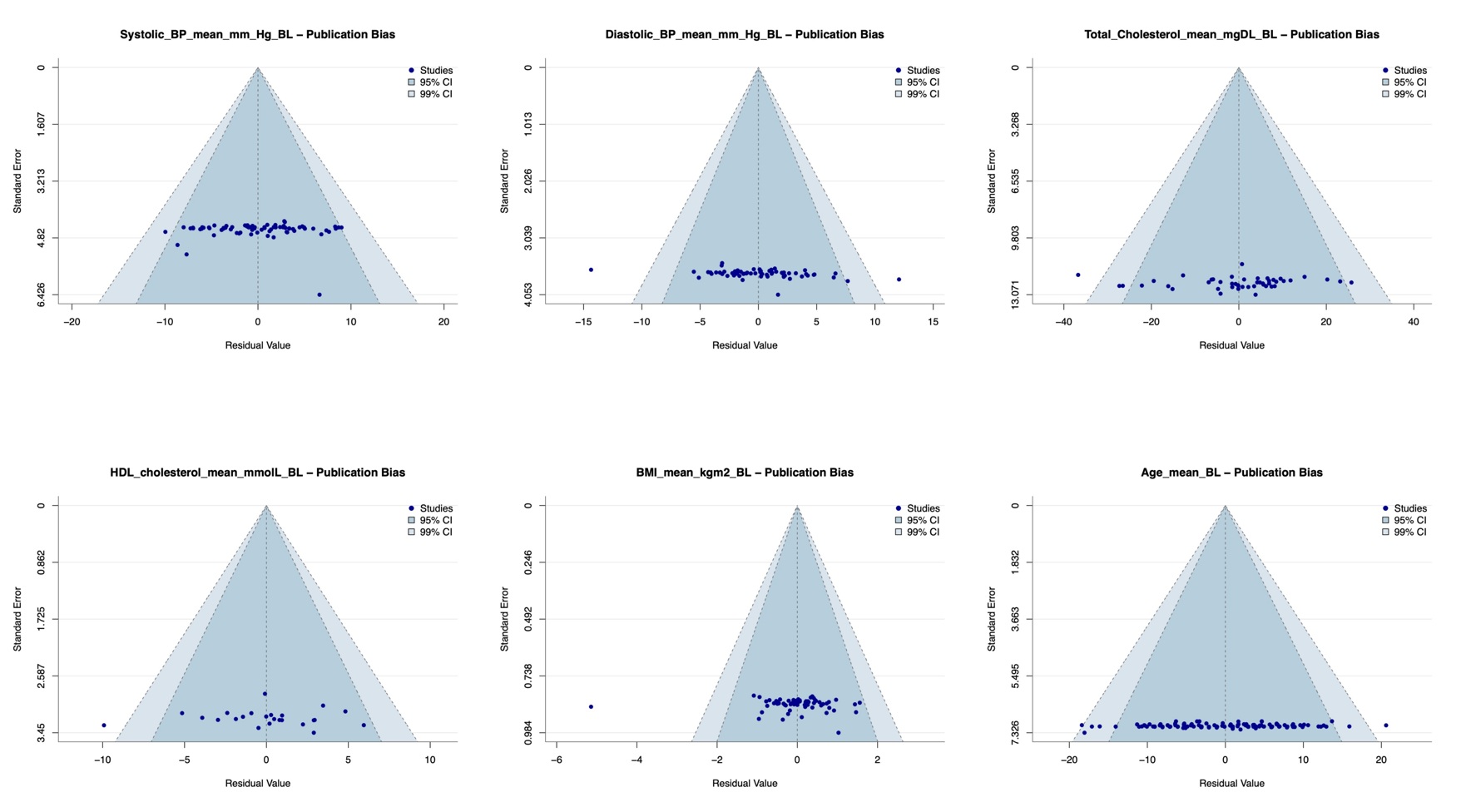


This figure presents a visual comparison of publication bias funnel plots for continuous risk factor variables. It includes systolic blood pressure, diastolic blood pressure, total cholesterol, HDL cholesterol, body mass index (BMI), and age. Each plot illustrates the distribution of studies, highlighting potential publication bias with shaded regions representing 95% and 99% confidence intervals. The 3x2 grid layout is used to facilitate an organized and comparative visual analysis of the data

# **Supplementary Table 6. Sensitivity analyses**

| Outcome | Model | N Studies | N Outliers | Max Effect Change |
| --- | --- | --- | --- | --- |
| CMBs | BMI kg/m² | 74 | 1 | 0.2 |
|  | Country Income Score | 74 | 1 | 0.1 |
|  | Current smoking prevalence | 74 | 1 | 0.1 |
|  | Diabetes prevalence | 74 | 1 | 0.5 |
|  | Diastolic BP mmHg | 74 | 1 | 3.1 |
|  | Female prevalence | 74 | 1 | 0.2 |
|  | Hypertension prevalence | 74 | 1 | 0.2 |
|  | Male prevalence | 74 | 1 | 0.2 |
|  | Systolic BP mmHg | 74 | 1 | 0.1 |
|  | base model | 74 | 1 | 0.1 |
| Lacunes | BMI kg/m² | 76 | 1 | 9.6 |
|  | Country Income Score | 76 | 1 | 0.6 |
|  | Current smoking prevalence | 76 | 1 | 0.3 |
|  | Diabetes prevalence | 76 | 1 | 0.5 |
|  | Diastolic BP mmHg | 76 | 1 | 2.1 |
|  | Female prevalence | 76 | 2 | 0.2 |
|  | Hypertension prevalence | 76 | 1 | 0.9 |
|  | Male prevalence | 76 | 1 | 0.7 |
|  | Systolic BP mmHg | 76 | 1 | 1.1 |
|  | base model | 76 | 1 | 0.1 |
| Microinfarcts | Current smoking prevalence | 32 | 0 | 0 |
|  | Diabetes prevalence | 32 | 0 | 0 |
|  | Female prevalence | 32 | 0 | 0 |
|  | Hyperlipidemia prevalence | 32 | 0 | 0 |
|  | Hypertension prevalence | 32 | 0 | 0 |
|  | Male prevalence | 32 | 0 | 0 |
|  | base model | 32 | 0 | 0 |
| WMH High | Base | 41 | 1 | 0.9 |
|  | Diabetes % | 41 | 0 | 0 |
|  | Male % | 41 | 0 | 0 |
|  | Smoking % | 41 | 0 | 0 |
| WMHvol | Current smoking | 12 | 0 | 0 |
|  | Diabetes prevalence | 18 | 0 | 0 |
|  | Female prevalence | 28 | 0 | 0 |
|  | Hypertension prevalence | 13 | 0 | 0 |
|  | Male prevalence | 28 | 0 | 0 |
|  | Systolic BP mmHg | 12 | 0 | 0 |
|  | Total Studies Meta Analysis | 35 | 1 | 2.6 |
|  | base model | 31 | 1 | 3.9 |

1. N Studies: number of studies included.
2. N Outliers: number of outlying studies identified.
3. Max Effect Change: maximum absolute change in effect estimate (%)..

# **Supplementary Table 7. Tier 1 Ethnicity Analyses based Study Inclusion ID Number**

Separate file provided.

# **Supplementary Table 8. Tier 2 Asian-only Ethnicity Analyses based Study Inclusion ID Number**

Separate file provided.

# **Supplementary Table 9. Full study Characteristics**

Separate file provided.

# **Supplementary Section 3. Key Equations**

1. **Logit Transformation of Proportions**

$$logit(p)=ln(\frac{p}{1-p})$$

- - Used for proportional outcomes (e.g., prevalence of CMBs, lacunes, microinfarcts, and categorical risk factors).
  - After meta-analysis or meta-regression, results can be back-transformed from the logit scale to the original proportion scale as:

$p=\frac{1}{1+e-logit(p)}.$

1. **General Meta-Regression Model**
   - In a (random-effects) meta-regression framework, the observed outcome (which can be a continuous measure or a logit-transformed proportion) in study ii is modeled as:

$$y_{i}=\beta_{0}+\beta_{1}X_{i1}+\beta_{2}X_{i2}+\ldots+u_{i}+\varepsilon_{i} ,$$

where

- - - $y_{i}$= outcome of interest for study ii (e.g., WMH volume, logit of prevalence),
    - $X_{i1},X_{i2},\ldots$= moderator variables (e.g., ethnicity, age, risk factors),
    - $u_{i}$= random effect for study ii (assumes $ui\sim N(0,\tau^{2})$),
    - $\varepsilon_{i}$= residual error term.

1. **Interaction Model**
   - When testing for an Ethnicity ×\times Risk Factor interaction in a meta-regression, an interaction term is added:

$$y_{i}=\beta_{0}+\beta_{1}\left( {Ethnicity}_{i} \right)+\beta_{2}\left( {RiskFactor}_{i} \right)+\beta_{3}\left( {Ethnicity}_{i}\times{RiskFactor}_{i} \right)+u_{i}+\varepsilon_{i}.$$

1. **Heterogeneity Statistics**
   - **Q statistic (**$\boldsymbol{x}^{\boldsymbol{2}}$**test for heterogeneity)**:

$$Q = \sum_{i=1}^{k} w_{i}{(y_{i}-\bar{y})}^{2}$$

where $w_{i}$are the study-specific weights (often inverse variance), $y_{i}$ is the effect estimate for study $i$, and $\bar{y}$is the weighted mean effect estimate.

- - **I² statistic (percentage of total variation due to heterogeneity)**:

$$I^{2}=\frac{Q-(k-1)}{Q}\times100\%,$$

where $k$ is the number of studies. By convention, $I^{2}>50\%$indicates substantial heterogeneity.

1. **Likelihood Ratio Test (LRT) for Model Comparisons**
   - Used to compare a base model (e.g., without interaction) against a more complex model (e.g., with Ethnicity $\times$ Risk Factor interaction). The test statistic is:

$$\Delta x^{2}=-2\left[ ln \left( L_{Base} \right)-\ln\left( L_{Complex} \right) \right],$$

which follows a chi-square distribution with degrees of freedom equal to the difference in the number of parameters between the two models.

**Notes:**

- When proportions $(p)$are used as outcomes, they are typically transformed via the logit function (Equation 1) before analysis, and back-transformed for interpretation.
- Meta-regression models (Equations 3 and 4) accommodate moderators (continuous or categorical), and random effects account for between-study heterogeneity.
- The Q and I² statistics (Equation 5) quantify and test for heterogeneity in the pooled estimates.
- The LRT (Equation 6) assesses whether more complex models (e.g., with interaction terms or nested random effects) significantly improve the fit over simpler models.

These equations collectively underpin the analytical framework described in the main text

# **Supplementary Table 10. Likelihood Test Ratio Findings for all outcomes**

| **Outcome** | **Model** | **Df** | **AIC** | **BIC** | **AICc** | **LogLik** | **LRT** | **p-value#** | **QE** | **Type** |
| --- | --- | --- | --- | --- | --- | --- | --- | --- | --- | --- |
| **Age (years)** | Full | 3 | 995.23 | 1004.12 | 995.4 | -494.61 | NA | NA | 1251287.83 | Continuous |
| **Age (years)** | Reduced | 2 | 1753.99 | 1759.92 | 1754.08 | -874.99 | 760.76 | <0.0001 | 1251287.83 | Continuous |
| **Systolic BP (mmHg)** | Full | 3 | 500.48 | 507.27 | 500.84 | -247.24 | NA | NA | 36819.44 | Continuous |
| **Systolic BP (mmHg)** | Reduced | 2 | 498.48 | 503 | 498.66 | -247.24 | 0 | 1 | 36819.44 | Continuous |
| **Diastolic BP (mmHg)** | Full | 3 | 358.91 | 365.39 | 359.31 | -176.46 | NA | NA | 8069.92 | Continuous |
| **Diastolic BP (mmHg)** | Reduced | 2 | 356.91 | 361.23 | 357.11 | -176.46 | 0 | 1 | 8069.92 | Continuous |
| **Total Cholesterol (mg/dL)** | Full | 3 | 387.58 | 393.06 | 388.15 | -190.79 | NA | NA | 13213.68 | Continuous |
| **Total Cholesterol (mg/dL)** | Reduced | 2 | 385.58 | 389.24 | 385.86 | -190.79 | 0 | 1 | 13213.68 | Continuous |
| **HDL Cholesterol (mmol/L)** | Full | 3 | 145.15 | 148.55 | 146.41 | -69.57 | NA | NA | 2583.92 | Continuous |
| **HDL Cholesterol (mmol/L)** | Reduced | 2 | 143.15 | 145.42 | 143.75 | -69.57 | 0 | 1 | 2583.92 | Continuous |
| **BMI (kg/m²)** | Full | 3 | 227.94 | 234.37 | 228.35 | -110.97 | NA | NA | 15371.45 | Continuous |
| **BMI (kg/m²)** | Reduced | 2 | 236.78 | 241.06 | 236.98 | -116.39 | 10.84 | 0.001 | 15371.45 | Continuous |
| **WMH Volume (cm³)** | Full | 3 | 125.94 | 129.35 | 127.2 | -59.97 | NA | NA | 6460.63 | Continuous |
| **WMH Volume (cm³)** | Reduced | 2 | 123.94 | 126.21 | 124.54 | -59.97 | 0 | 1 | 6460.63 | Continuous |
| **Hypertension (n)** | Full | 3 | 397.08 | 404.93 | 397.33 | -195.54 | NA | NA | 21158.42 | Prevalence |
| **Hypertension (n)** | Reduced | 2 | 395.08 | 400.31 | 395.2 | -195.54 | 0 | 1 | 21158.42 | Prevalence |
| **Diabetes (n)** | Full | 3 | 274.67 | 283.06 | 274.88 | -134.34 | NA | NA | 13532.71 | Prevalence |
| **Diabetes (n)** | Reduced | 2 | 272.67 | 278.26 | 272.77 | -134.34 | 0 | 1 | 13532.71 | Prevalence |
| **Hyperlipidemia (n)** | Full | 3 | 164.9 | 171.68 | 165.25 | -79.45 | NA | NA | 5740.23 | Prevalence |
| **Hyperlipidemia (n)** | Reduced | 2 | 162.9 | 167.42 | 163.07 | -79.45 | 0 | 1 | 5740.23 | Prevalence |
| **Hypercholesterolemia (n)** | Full | 3 | 64.33 | 66.65 | 66.33 | -29.17 | NA | NA | 1870.99 | Prevalence |
| **Hypercholesterolemia (n)** | Reduced | 2 | 62.33 | 63.88 | 63.26 | -29.17 | 0 | 1 | 1870.99 | Prevalence |
| **BMI > 30 kg/m² (n)** | Full | 3 | 33.39 | 34.58 | 36.82 | -13.69 | NA | NA | 605.94 | Prevalence |
| **BMI > 30 kg/m² (n)** | Reduced | 2 | 31.39 | 32.19 | 32.89 | -13.69 | 0 | 1 | 605.94 | Prevalence |
| **High Alcohol Consumption (n)** | Full | 3 | 113.9 | 119.12 | 114.54 | -53.95 | NA | NA | 6544.05 | Prevalence |
| **High Alcohol Consumption (n)** | Reduced | 2 | 208.46 | 211.93 | 208.77 | -102.23 | 96.55 | <0.0001 | 6544.05 | Prevalence |
| **Current Smoking (n)** | Full | 3 | 245.08 | 252.8 | 245.34 | -119.54 | NA | NA | 21583.12 | Prevalence |
| **Current Smoking (n)** | Reduced | 2 | 322.38 | 327.53 | 322.51 | -159.19 | 79.3 | <0.0001 | 21583.12 | Prevalence |
| **Lacunes (n)** | Full | 3 | 163.6 | 169.93 | 164.02 | -78.8 | NA | NA | 15997.61 | Prevalence |
| **Lacunes (n)** | Reduced | 2 | 161.6 | 165.82 | 161.81 | -78.8 | 0 | 1 | 15997.61 | Prevalence |
| **Microinfarcts (n)** | Full | 3 | 23.17 | 23.41 | 29.17 | -8.58 | NA | NA | 800.96 | Prevalence |
| **Microinfarcts (n)** | Reduced | 2 | 21.17 | 21.33 | 23.57 | -8.58 | 0 | 1 | 800.96 | Prevalence |
| **CMBs (n)** | Full | 3 | 153.51 | 159.69 | 153.96 | -73.76 | NA | NA | 2445.2 | Prevalence |
| **CMBs (n)** | Reduced | 2 | 151.51 | 155.63 | 151.73 | -73.76 | 0 | 1 | 2445.2 | Prevalence |

1. Df: degrees of freedom.
2. AIC: Akaike information criterion.
3. BIC: Bayesian information criterion.
4. AICc: small-sample corrected AIC.
5. LogLik: log-likelihood.
6. LRT: likelihood-ratio test statistic.
7. p-value: significance of LRT.
8. QE: test statistic for residual heterogeneity.

# **Supplementary Table 11. Overall CSVD effect estimates**

| CSVD Outcome | Model Type | Moderators | K | N | Meta-analysis finding (95% CI) | Heterogeneity | Age association |
| --- | --- | --- | --- | --- | --- | --- | --- |
| WMH Volume (cm3) | Reduced | Ethnicity Type + Age | 28 | 17118 | 4.30 (3.38, 5.48) | I² = 99.4%, τ² = 0.390 | β = 0.062, p = 0.000 |
| Odds of High WMH Burden (Fazekas) | Reduced | Ethnicity Type + Age | 42 | 33918 | 0.27 (0.21, 0.34) | I² = 98.4%, τ² = 0.568 | β = 0.021, p = 0.263 |
| Lacunes (Prevalence) | Reduced | Ethnicity Type + Age | 73 | 1977965 | 12.3% (10.4, 14.5) | I² = 99.5%, τ² = 0.645 | β = 0.051, p = 0.001 |
| Microinfarcts (Prevalence) | Reduced | Ethnicity Type + Age | 9 | 11838 | 13.2% (9.9, 17.4) | I² = 96.9%, τ² = 0.235 | β = 0.069, p = 0.018 |
| CMBs (Prevalence) | Reduced | Ethnicity Type + Age | 74 | 81771 | 11.2% (9.6, 13.0) | I² = 98.2%, τ² = 0.583 | β = 0.040, p = 0.007 |

1. *K*: number of studies.
2. *N*: total participants.
3. *95% CI*: confidence interval.
4. *Heterogeneity*: I² statistic.
5. *Age association*: regression coefficient (p-value) for age.

# **Supplementary Table 12. Overall Risk Factor Effect estimates**

| Outcome | Outcome Type | Moderators | K | N | Meta-analysis findings | Heterogeneity | Association with cohort/study mean age |
| --- | --- | --- | --- | --- | --- | --- | --- |
| Hypertension Count | Full | Ethnicity Type + Age | 121 | 1542231 | 33.1% (27.5, 39.2) | I² = 99.8%, τ² = 2.299 | β = 0.041, p = 0.009 |
| Diabetes Count | Full | Ethnicity Type + Age | 137 | 2043361 | 13.3% (12.2, 14.5) | I² = 99.0%, τ² = 0.319 | β = 0.038, p = 0.000 |
| Hyperlipidemia Count | Reduced | Ethnicity Type + Age | 83 | 1500050 | 33.4% (29.8, 37.3) | I² = 99.1%, τ² = 0.608 | β = 0.026, p = 0.051 |
| Hypercholesterolemia Count | Full | Ethnicity Type + Age | 22 | 13916 | 40.6% (26.4, 56.5) | I² = 99.5%, τ² = 2.306 | β = 0.000, p = 0.996 |
| BMI Over 30 Kgm2 Count | Reduced | Ethnicity Type + Age | 15 | 11776 | 14.0% (9.6, 19.9) | I² = 98.2%, τ² = 0.686 | β = -0.001, p = 0.985 |
| High Alcohol Consumption Count | Full | Ethnicity Type + Age | 52 | 544495 | 27.7% (24.1, 31.6) | I² = 99.6%, τ² = 0.766 | β = -0.002, p = 0.939 |
| Current Smoking Count | Full | Ethnicity Type + Age | 115 | 605863 | 15.4% (13.5, 17.5) | I² = 99.2%, τ² = 0.648 | β = -0.027, p = 0.001 |
| Age | Full | Ethnicity Type + Age | 166 | 2074326 | 62.72 (60.64, 64.87) | I² = 100.0%, τ² = 0.049 | Not included |
| Systolic Bp Mm Hg | Full | Ethnicity Type + Age | 82 | 570410 | 130.92 (128.41, 133.49) | I² = 99.9%, τ² = 0.013 | β = 0.072, p = 0.000 |
| Diastolic Bp Mm Hg | Full | Ethnicity Type + Age | 82 | 570410 | 77.39 (76.09, 78.73) | I² = 99.9%, τ² = 0.004 | β = 0.021, p = 0.041 |
| Total Cholesterol Mgdl | Full | Ethnicity Type + Age | 46 | 191632 | 190.46 (185.20, 195.84) | I² = 99.9%, τ² = 0.016 | β = -0.002, p = 0.911 |
| Hdl Cholesterol Mmoll | Full | Ethnicity Type + Age | 33 | 164118 | 1.35 (1.26, 1.44) | I² = 99.9%, τ² = 0.015 | β = -0.012, p = 0.284 |
| BMI Kgm2 | Full | Ethnicity Type + Age | 83 | 161454 | 24.60 (23.83, 25.37) | I² = 99.9%, τ² = 0.026 | β = 0.030, p = 0.005 |

1. *K*: number of studies.
2. N: total participants.
3. Meta-analysis findings: estimated effect (95% CI).
4. Heterogeneity: I² statistic.
5. Association with cohort/study mean age: coefficient (p-value)

# **Supplementary Figure 5 Tier 2 Asian-only CSVD Outcome**





Forest plots showing pooled meta-analytic estimates of cerebral small-vessel disease (CSVD) markers across Asian subgroups (Chinese, Japanese, Korean). Outcomes include white-matter hyperintensity (WMH) volume and Fazekas grade, lacunes, and cerebral microbleeds (CMBs). Error bars represent 95 % CIs, and pooled effects are derived from random-effects meta-regression models.

# **Supplementary Figure 6 Tier 2 Asian-only Continuous Risk Factor Outcome**





Forest plots comparing continuous vascular-risk factors (e.g., systolic/diastolic blood pressure, BMI, cholesterol, glucose) across Asian subgroups. Estimates are standardized mean differences with 95 % CIs, pooled using random-effects models.

# **Supplementary Figure 7 Tier 2 Asian-only Prevalence Risk Factor Outcome**





Forest plots comparing prevalence of vascular-risk factors (hypertension, diabetes, obesity, hyperlipidaemia, smoking, alcohol consumption) across Asian subgroups. Odds ratios are displayed with 95 % CIs from random-effects meta-analyses.

# **Supplementary Table 13. Omnibus χ² Tests for Tier 1 Ethnicity Effects on CSVD Imaging Markers and Risk Factors**

| Outcome | QM | DF | p value |
| --- | --- | --- | --- |
| WMH Volume | 3.2857 | 193 | 0.35 |
| Odds of High WMH Burden | 0.0097 | 512 | 0.995 |
| Lacunes | 2.8711 | 103 | 0.09 |
| Microinfarcts | 0.1167 | 321 | 0.733 |
| CMBs | 5.6474 | 952 | 0.059 |
| Age mean | 1.5377 | 43 | 0.674 |
| Systolic BP mean mm Hg | 2.7573 | 71 | 0.097 |
| Diastolic BP mean mm Hg | 0.0004 | 80 | 0.984 |
| Total Cholesterol mean mgDL | 17.0183 | 331 | <0.001 |
| HDL cholesterol mean mmolL | 1.5366 | 251 | 0.215 |
| BMI mean kgm2 | 103.7995 | 541 | <0.001 |
| Hypertension | 2.1472 | 903 | 0.542 |
| Diabetes | 180.488 | 973 | <0.001 |
| Hyperlipidaemia | 1.2047 | 71 | 0.272 |
| Hypercholesterolemia | 3.1722 | 383 | 0.366 |
| BMI over 30 kgm2 | 3.2969 | 792 | 0.192 |
| High alcohol consumption | 0.446 | 581 | 0.504 |
| Current smoking | 7.4879 | 123 | 0.058 |

1. *QM*: test statistic for moderators.
2. *Df*: degrees of freedom.
3. *p value*: significance of QM.

# **Supplementary Table 14. Pairwise Tier 1 Ethnic Comparisons of CSVD Outcomes**

| Outcome | Contrast | OR | p | SE | z-ratio | Df | asymp.LCL | asymp.UCL |
| --- | --- | --- | --- | --- | --- | --- | --- | --- |
| WMH Volume (Base) | Asian / White | 1.32 | 0.8253 | 0.335 | 1.091 | Inf | 0.718 | 2.424 |
| WMH Volume (Base) | Black / White | 1.093 | 0.8346 | 0.09 | 1.084 | Inf | 0.898 | 1.331 |
| WMH Volume (Base) | Hispanic / White | 0.91 | 1 | 0.14 | -0.61 | Inf | 0.63 | 1.316 |
| Odds of High WMH Burden (Base) | Asian / White | 1.034 | 1 | 0.38 | 0.09 | Inf | 0.453 | 2.359 |
| Odds of High WMH Burden (Base) | Hispanic / White | 1.009 | 1 | 0.612 | 0.015 | Inf | 0.259 | 3.933 |
| Lacunes (Base) | Asian / White | 1.637 | 0.0902 | 0.476 | 1.694 | Inf | 0.926 | 2.896 |
| Microinfarcts (Base) | Asian / White | 1.198 | 0.7326 | 0.634 | 0.342 | Inf | 0.425 | 3.382 |
| CMBs (Base) | Asian / White | 1.236 | 0.7696 | 0.301 | 0.869 | Inf | 0.716 | 2.133 |
| CMBs (Base) | Hispanic / White | 0.724 | 0.8962 | 0.308 | -0.759 | Inf | 0.279 | 1.879 |
| Hypertension | Asian / White | 1.166 | 1 | 0.404 | 0.442 | Inf | 0.509 | 2.672 |
| Hypertension | Black / White | 2.241 | 0.7231 | 1.543 | 1.172 | Inf | 0.431 | 11.649 |
| Hypertension | Hispanic / White | 0.667 | 1 | 0.466 | -0.579 | Inf | 0.125 | 3.552 |
| Diabetes | Asian / White | 2.012 | <0.0001 | 0.201 | 7.009 | Inf | 1.584 | 2.554 |
| Diabetes | Black / White | 2.574 | <0.0001 | 0.209 | 11.646 | Inf | 2.119 | 3.126 |
| Diabetes | Hispanic / White | 3.566 | <0.0001 | 0.552 | 8.217 | Inf | 2.462 | 5.164 |
| Hypercholesterolemia | Asian / White | 0.297 | 0.439 | 0.248 | -1.453 | Inf | 0.04 | 2.196 |
| Hypercholesterolemia | Black / White | 0.577 | 0.5168 | 0.232 | -1.365 | Inf | 0.22 | 1.514 |
| Hypercholesterolemia | Hispanic / White | 0.672 | 1 | 0.333 | -0.802 | Inf | 0.205 | 2.2 |
| BMI over 30 kg/m² | Asian / White | 0.89 | 1 | 0.434 | -0.24 | Inf | 0.298 | 2.654 |
| BMI over 30 kg/m² | Hispanic / White | 2.522 | 0.2404 | 1.501 | 1.554 | Inf | 0.664 | 9.577 |
| High Alcohol Consumption | Asian / White | 0.836 | 0.5043 | 0.224 | -0.668 | Inf | 0.494 | 1.414 |
| Current Smoking | Asian / White | 1.364 | 0.2236 | 0.238 | 1.783 | Inf | 0.899 | 2.07 |
| Current Smoking | Black / White | 1.179 | 1 | 0.316 | 0.615 | Inf | 0.621 | 2.24 |
| Current Smoking | Hispanic / White | 0.653 | 0.4719 | 0.197 | -1.414 | Inf | 0.317 | 1.344 |
| Hyperlipidemia | Asian / White | 0.666 | 0.2724 | 0.247 | -1.098 | Inf | 0.322 | 1.376 |
| Age (mean) | Asian / Black | 0.99 | 1 | 0.039 | -0.248 | Inf | 0.892 | 1.099 |
| Age (mean) | Asian / Hispanic | 0.973 | 1 | 0.05 | -0.529 | Inf | 0.851 | 1.114 |
| Age (mean) | Asian / White | 0.968 | 1 | 0.03 | -1.073 | Inf | 0.893 | 1.049 |
| Age (mean) | Black / Hispanic | 0.983 | 1 | 0.049 | -0.346 | Inf | 0.862 | 1.121 |
| Age (mean) | Black / White | 0.977 | 1 | 0.03 | -0.753 | Inf | 0.901 | 1.06 |
| Age (mean) | Hispanic / White | 0.994 | 1 | 0.047 | -0.124 | Inf | 0.877 | 1.127 |
| Systolic BP (mean) | Asian / White | 0.964 | 0.0968 | 0.021 | -1.661 | Inf | 0.924 | 1.007 |
| Diastolic BP (mean) | Asian / White | 1.001 | 0.9844 | 0.031 | 0.019 | Inf | 0.941 | 1.064 |
| Total Cholesterol (mean) | Asian / White | 0.876 | <0.0001 | 0.028 | -4.125 | Inf | 0.822 | 0.933 |
| HDL Cholesterol (mean) | Asian / White | 0.907 | 0.2151 | 0.072 | -1.24 | Inf | 0.776 | 1.059 |
| BMI (mean) | Asian / White | 0.904 | <0.0001 | 0.009 | -10.188 | Inf | 0.887 | 0.922 |

1. *OR*: odds ratio.
2. *p*: p-value for contrast.
3. *SE*: standard error.
4. *z-ratio*: z test statistic.
5. *Df*: degrees of freedom.
6. *asymp.LCL*: asymptotic lower 95% confidence limit.
7. *asymp.UCL*: asymptotic upper 95% confidence limit

# **Supplementary Table 15. On versus All other Tier 1 Ethnic group contrasts Findings**

| Outcome | Ethnicity | Contrast | Estimate | SE | Lower CI | Upper CI | p value |
| --- | --- | --- | --- | --- | --- | --- | --- |
| WMH Volume | Asian | Asian vs others | 1.322 | 0.338 | 0.8 | 2.183 | 0.276 |
| WMH Volume | Black | Black vs others | 1.028 | 0.123 | 0.814 | 1.3 | 0.815 |
| WMH Volume | Hispanic | Hispanic vs others | 0.806 | 0.133 | 0.583 | 1.113 | 0.19 |
| WMH Volume | White | White vs others | 0.913 | 0.102 | 0.734 | 1.136 | 0.414 |
| Odds of High WMH Burden | Asian | Asian vs others | 1.029 | 0.338 | 0.541 | 1.957 | 0.93 |
| Odds of High WMH Burden | Hispanic | Hispanic vs others | 0.993 | 0.527 | 0.35 | 2.811 | 0.989 |
| Odds of High WMH Burden | White | White vs others | 0.979 | 0.422 | 0.421 | 2.279 | 0.961 |
| Hypertension | Asian | Asian vs others | 1.019 | 0.365 | 0.505 | 2.058 | 0.957 |
| Hypertension | Black | Black vs others | 2.437 | 1.618 | 0.663 | 8.955 | 0.18 |
| Hypertension | Hispanic | Hispanic vs others | 0.484 | 0.325 | 0.13 | 1.804 | 0.28 |
| Hypertension | White | White vs others | 0.831 | 0.353 | 0.361 | 1.912 | 0.663 |
| Diabetes | Asian | Asian vs others | 0.961 | 0.101 | 0.782 | 1.18 | 0.703 |
| Diabetes | Black | Black vs others | 1.335 | 0.118 | 1.123 | 1.587 | 0.001 |
| Diabetes | Hispanic | Hispanic vs others | 2.061 | 0.303 | 1.545 | 2.75 | <0.0001 |
| Diabetes | White | White vs others | 0.378 | 0.03 | 0.324 | 0.442 | <0.0001 |
| Hyperlipidemia | Asian | Asian vs others | 0.666 | 0.247 | 0.322 | 1.376 | 0.272 |
| Hyperlipidemia | White | White vs others | 1.501 | 0.556 | 0.727 | 3.101 | 0.272 |
| Hypercholesterolemia | Asian | Asian vs others | 0.407 | 0.315 | 0.09 | 1.854 | 0.245 |
| Hypercholesterolemia | Black | Black vs others | 0.987 | 0.421 | 0.428 | 2.277 | 0.976 |
| Hypercholesterolemia | Hispanic | Hispanic vs others | 1.21 | 0.531 | 0.512 | 2.858 | 0.664 |
| Hypercholesterolemia | White | White vs others | 2.055 | 0.916 | 0.858 | 4.923 | 0.106 |
| BMI over 30 kgm2 | Asian | Asian vs others | 0.56 | 0.257 | 0.228 | 1.376 | 0.206 |
| BMI over 30 kgm2 | Hispanic | Hispanic vs others | 2.674 | 1.459 | 0.918 | 7.793 | 0.071 |
| BMI over 30 kgm2 | White | White vs others | 0.668 | 0.303 | 0.274 | 1.625 | 0.373 |
| High alcohol consumption | Asian | Asian vs others | 0.836 | 0.224 | 0.494 | 1.414 | 0.504 |
| High alcohol consumption | White | White vs others | 1.196 | 0.321 | 0.707 | 2.024 | 0.504 |
| Current smoking | Asian | Asian vs others | 1.489 | 0.306 | 0.994 | 2.228 | 0.053 |
| Current smoking | Black | Black vs others | 1.226 | 0.317 | 0.738 | 2.036 | 0.432 |
| Current smoking | Hispanic | Hispanic vs others | 0.557 | 0.157 | 0.321 | 0.968 | 0.038 |
| Current smoking | White | White vs others | 0.984 | 0.173 | 0.697 | 1.389 | 0.926 |
| Age mean | Asian | Asian vs others | 0.977 | 0.032 | 0.916 | 1.042 | 0.478 |
| Age mean | Black | Black vs others | 0.99 | 0.032 | 0.93 | 1.054 | 0.75 |
| Age mean | Hispanic | Hispanic vs others | 1.013 | 0.046 | 0.927 | 1.107 | 0.778 |
| Age mean | White | White vs others | 1.021 | 0.026 | 0.972 | 1.073 | 0.414 |
| Systolic BP mean mm Hg | Asian | Asian vs others | 0.964 | 0.021 | 0.924 | 1.007 | 0.097 |
| Systolic BP mean mm Hg | White | White vs others | 1.037 | 0.023 | 0.993 | 1.083 | 0.097 |
| Diastolic BP mean mm Hg | Asian | Asian vs others | 1.001 | 0.031 | 0.941 | 1.064 | 0.984 |
| Diastolic BP mean mm Hg | White | White vs others | 0.999 | 0.031 | 0.94 | 1.062 | 0.984 |
| Total Cholesterol mean mgDL | Asian | Asian vs others | 0.876 | 0.028 | 0.822 | 0.933 | <0.0001 |
| Total Cholesterol mean mgDL | White | White vs others | 1.142 | 0.037 | 1.072 | 1.216 | <0.0001 |
| HDL cholesterol mean mmolL | Asian | Asian vs others | 0.907 | 0.072 | 0.776 | 1.059 | 0.215 |
| HDL cholesterol mean mmolL | White | White vs others | 1.103 | 0.087 | 0.945 | 1.288 | 0.215 |
| BMI mean kgm2 | Asian | Asian vs others | 0.904 | 0.009 | 0.887 | 0.922 | <0.0001 |
| BMI mean kgm2 | White | White vs others | 1.106 | 0.011 | 1.085 | 1.128 | <0.0001 |
| Lacunes | Asian | Asian vs others | 1.637 | 0.476 | 0.926 | 2.896 | 0.09 |
| Lacunes | White | White vs others | 0.611 | 0.178 | 0.345 | 1.08 | 0.09 |
| Microinfarcts | Asian | Asian vs others | 1.198 | 0.634 | 0.425 | 3.382 | 0.733 |
| Microinfarcts | White | White vs others | 0.835 | 0.442 | 0.296 | 2.355 | 0.733 |
| CMBs | Asian | Asian vs others | 1.452 | 0.231 | 1.063 | 1.983 | 0.019 |
| CMBs | Hispanic | Hispanic vs others | 0.651 | 0.222 | 0.334 | 1.272 | 0.209 |
| CMBs | White | White vs others | 1.057 | 0.334 | 0.569 | 1.963 | 0.86 |

1. *Estimate*: estimated marginal mean difference (or log-OR).
2. *SE*: standard error.
3. *Lower CI*: lower 95% confidence limit.
4. *Upper CI*: upper 95% confidence limit.
5. *p value*: significance.

# **Supplementary Table 16. Omnibus χ² Tests for Tier 2 Asian Ethnicity Effects on CSVD Imaging Markers and Risk Factors**

| Outcome | QM | DF | p value |
| --- | --- | --- | --- |
| Odds of High WMH Burden | 2.73 | 862 | 0.255 |
| WMH Volume | 0.1929 | 681 | 0.661 |
| Lacunes | 7.0196 | 704 | 0.135 |
| Microinfarcts | 0.2097 | 21 | 0.647 |
| CMBs | 18.3642 | 673 | <0.001 |
| Age mean | 157.8806 | 834 | <0.001 |
| Current smoking | 6.2329 | 133 | 0.101 |
| Systolic BP mean mm Hg | 1.6199 | 762 | 0.445 |
| Diastolic BP mean mm Hg | 2.9909 | 112 | 0.224 |
| Total Cholesterol mean mgDL | 17.8783 | 872 | <0.001 |
| HDL cholesterol mean mmolL | 15.6685 | 932 | <0.001 |
| BMI mean kgm2 | 40.4425 | 383 | <0.001 |
| Hypertension | 0.8228 | 533 | 0.844 |
| Diabetes | 15.8769 | 724 | 0.0032 |
| Hyperlipidaemia | 1.8383 | 613 | 0.607 |
| High alcohol consumption | 2.4946 | 352 | 0.287 |

1. *QM*: test statistic for moderators.
2. *Df*: degrees of freedom.
3. *p value*: significance of QM.

# **Supplementary Table 17. Pairwise Tier 2 Asian Ethnic Comparisons**

| Outcome | Contrast | OR | p | SE | z-ratio | Df | asymp.LCL | asymp.UCL |
| --- | --- | --- | --- | --- | --- | --- | --- | --- |
| Lacunes (Base) | Chinese (Mainland China) / Chinese (Hong Kong) | 1.154 | 1 | 0.393 | 0.42 | Inf | 0.493 | 2.7 |
| Lacunes (Base) | Chinese (Taiwan) / Chinese (Hong Kong) | 0.726 | 1 | 0.409 | -0.568 | Inf | 0.178 | 2.963 |
| Lacunes (Base) | Japanese (Japan) / Chinese (Hong Kong) | 1.266 | 1 | 0.464 | 0.643 | Inf | 0.506 | 3.163 |
| Lacunes (Base) | South Korean (South Korea) / Chinese (Hong Kong) | 0.656 | 0.4024 | 0.169 | -1.642 | Inf | 0.345 | 1.246 |
| Microinfarcts (Base) | South Korean (South Korea) / Japanese (Japan) | 1.241 | 0.647 | 0.584 | 0.458 | Inf | 0.493 | 3.122 |
| CMBs (Base) | Chinese (Mainland China) / Chinese (Hong Kong) | 1.13 | 1 | 0.389 | 0.355 | Inf | 0.495 | 2.578 |
| CMBs (Base) | Japanese (Japan) / Chinese (Hong Kong) | 0.476 | 0.1478 | 0.18 | -1.966 | Inf | 0.193 | 1.175 |
| CMBs (Base) | South Korean (South Korea) / Chinese (Hong Kong) | 0.534 | 0.1287 | 0.165 | -2.025 | Inf | 0.254 | 1.121 |
| Hypertension | Chinese (Mainland China) / Chinese (Hong Kong) | 0.521 | 1 | 0.444 | -0.766 | Inf | 0.068 | 3.999 |
| Hypertension | Japanese (Japan) / Chinese (Hong Kong) | 0.496 | 1 | 0.422 | -0.824 | Inf | 0.065 | 3.805 |
| Hypertension | South Korean (South Korea) / Chinese (Hong Kong) | 0.457 | 1 | 0.401 | -0.893 | Inf | 0.056 | 3.733 |
| Diabetes | Chinese (Mainland China) / Chinese (Hong Kong) | 0.818 | 1 | 0.277 | -0.595 | Inf | 0.351 | 1.904 |
| Diabetes | Chinese (Taiwan) / Chinese (Hong Kong) | 0.654 | 1 | 0.279 | -0.994 | Inf | 0.225 | 1.901 |
| Diabetes | Japanese (Japan) / Chinese (Hong Kong) | 0.55 | 0.3141 | 0.187 | -1.759 | Inf | 0.235 | 1.286 |
| Diabetes | South Korean (South Korea) / Chinese (Hong Kong) | 0.983 | 1 | 0.341 | -0.05 | Inf | 0.413 | 2.338 |
| Hyperlipidemia | Chinese (Mainland China) / Chinese (Hong Kong) | 1.043 | 1 | 0.482 | 0.091 | Inf | 0.345 | 3.155 |
| Hyperlipidemia | Japanese (Japan) / Chinese (Hong Kong) | 0.837 | 1 | 0.395 | -0.377 | Inf | 0.27 | 2.591 |
| Hyperlipidemia | South Korean (South Korea) / Chinese (Hong Kong) | 0.8 | 1 | 0.383 | -0.466 | Inf | 0.254 | 2.521 |
| High Alcohol Consumption | Japanese (Japan) / Chinese (Mainland China) | 1.622 | 0.2734 | 0.527 | 1.488 | Inf | 0.783 | 3.359 |
| High Alcohol Consumption | South Korean (South Korea) / Chinese (Mainland China) | 1.514 | 0.6574 | 0.643 | 0.977 | Inf | 0.585 | 3.92 |
| Current Smoking | Chinese (Taiwan) / Chinese (Mainland China) | 0.608 | 0.8677 | 0.285 | -1.06 | Inf | 0.198 | 1.87 |
| Current Smoking | Japanese (Japan) / Chinese (Mainland China) | 0.688 | 0.1468 | 0.131 | -1.969 | Inf | 0.437 | 1.084 |
| Current Smoking | South Korean (South Korea) / Chinese (Mainland China) | 0.634 | 0.1058 | 0.137 | -2.105 | Inf | 0.378 | 1.064 |
| Age (mean) | Chinese (Hong Kong) / South Korean (South Korea) | 1.104 | <0.0001 | 0.009 | 12.482 | Inf | 1.08 | 1.129 |
| Age (mean) | Chinese (Hong Kong) / Chinese (Mainland China) | 1.098 | 0.0092 | 0.031 | 3.313 | Inf | 1.014 | 1.189 |
| Age (mean) | Chinese (Hong Kong) / Japanese (Japan) | 1.064 | 0.3679 | 0.032 | 2.088 | Inf | 0.979 | 1.156 |
| Age (mean) | Chinese (Hong Kong) / Chinese (Taiwan) | 1.04 | 1 | 0.063 | 0.645 | Inf | 0.877 | 1.234 |
| Age (mean) | Chinese (Mainland China) / Chinese (Taiwan) | 0.947 | 1 | 0.056 | -0.918 | Inf | 0.802 | 1.118 |
| Age (mean) | Chinese (Mainland China) / Japanese (Japan) | 0.969 | 1 | 0.025 | -1.219 | Inf | 0.901 | 1.042 |
| Age (mean) | Chinese (Mainland China) / South Korean (South Korea) | 1.006 | 1 | 0.028 | 0.213 | Inf | 0.931 | 1.086 |
| Age (mean) | Chinese (Taiwan) / Japanese (Japan) | 1.023 | 1 | 0.061 | 0.38 | Inf | 0.865 | 1.21 |
| Age (mean) | Chinese (Taiwan) / South Korean (South Korea) | 1.062 | 1 | 0.064 | 0.993 | Inf | 0.896 | 1.258 |
| Age (mean) | Japanese (Japan) / South Korean (South Korea) | 1.038 | 1 | 0.03 | 1.293 | Inf | 0.957 | 1.126 |
| Systolic BP (mean) | Chinese (Mainland China) / Japanese (Japan) | 1.047 | 0.6116 | 0.038 | 1.271 | Inf | 0.96 | 1.142 |
| Systolic BP (mean) | Chinese (Mainland China) / South Korean (South Korea) | 1.026 | 1 | 0.039 | 0.663 | Inf | 0.936 | 1.124 |
| Systolic BP (mean) | Japanese (Japan) / South Korean (South Korea) | 0.98 | 1 | 0.034 | -0.597 | Inf | 0.902 | 1.064 |
| Diastolic BP (mean) | Japanese (Japan) / South Korean (South Korea) | 0.719 | 0.3576 | 0.152 | -1.558 | Inf | 0.433 | 1.193 |
| Diastolic BP (mean) | Chinese (Mainland China) / Japanese (Japan) | 1.338 | 0.5481 | 0.292 | 1.333 | Inf | 0.793 | 2.257 |
| Diastolic BP (mean) | Chinese (Mainland China) / South Korean (South Korea) | 0.962 | 1 | 0.223 | -0.165 | Inf | 0.552 | 1.678 |
| Total Cholesterol (mean) | Chinese (Mainland China) / Japanese (Japan) | 0.849 | <0.0001 | 0.033 | -4.224 | Inf | 0.774 | 0.932 |
| Total Cholesterol (mean) | Chinese (Mainland China) / South Korean (South Korea) | 0.922 | 0.066 | 0.033 | -2.29 | Inf | 0.847 | 1.004 |
| Total Cholesterol (mean) | Japanese (Japan) / South Korean (South Korea) | 1.086 | 0.0798 | 0.04 | 2.217 | Inf | 0.993 | 1.187 |
| HDL Cholesterol (mean) | Chinese (Mainland China) / Japanese (Japan) | 0.732 | 0.0012 | 0.065 | -3.53 | Inf | 0.593 | 0.905 |
| HDL Cholesterol (mean) | Japanese (Japan) / South Korean (South Korea) | 1.162 | 0.038 | 0.07 | 2.493 | Inf | 1.006 | 1.342 |
| HDL Cholesterol (mean) | Chinese (Mainland China) / South Korean (South Korea) | 0.851 | 0.2763 | 0.082 | -1.684 | Inf | 0.677 | 1.07 |
| BMI (mean) | Chinese (Mainland China) / Japanese (Japan) | 1.051 | <0.0001 | 0.008 | 6.248 | Inf | 1.029 | 1.073 |
| BMI (mean) | Japanese (Japan) / South Korean (South Korea) | 0.972 | 0.009 | 0.009 | -3.174 | Inf | 0.95 | 0.995 |
| BMI (mean) | Chinese (Taiwan) / Japanese (Japan) | 1.04 | 0.063 | 0.016 | 2.559 | Inf | 0.999 | 1.084 |
| BMI (mean) | Chinese (Mainland China) / South Korean (South Korea) | 1.022 | 0.0966 | 0.009 | 2.407 | Inf | 0.998 | 1.046 |
| BMI (mean) | Chinese (Mainland China) / Chinese (Taiwan) | 1.01 | 1 | 0.016 | 0.649 | Inf | 0.969 | 1.053 |
| BMI (mean) | Chinese (Taiwan) / South Korean (South Korea) | 1.012 | 1 | 0.016 | 0.708 | Inf | 0.969 | 1.056 |

1. *OR*: odds ratio.
2. *p*: p-value for contrast.
3. *SE*: standard error.
4. *z-ratio*: z test statistic.
5. *Df*: degrees of freedom.
6. *asymp.LCL*: asymptotic lower 95% confidence limit.
7. *asymp.UCL*: asymptotic upper 95% confidence limit.

# **Supplementary Table 18. On versus All other Tier 2 Asian Ethnic group contrasts Findings**

| Outcome | Ethnicity | Contrast | Estimate | SE | Lower CI | Upper CI | p value |
| --- | --- | --- | --- | --- | --- | --- | --- |
| WMH Volume | Chinese (Mainland China) | Chinese (Mainland China) vs others | 1.386 | 1.03 | 0.323 | 5.951 | 0.661 |
| WMH Volume | South Korean (South Korea) | South Korean (South Korea) vs others | 0.721 | 0.536 | 0.168 | 3.097 | 0.661 |
| Odds of High WMH Burden | Chinese (Mainland China) | Chinese (Mainland China) vs others | 1.63 | 0.494 | 0.901 | 2.952 | 0.107 |
| Odds of High WMH Burden | Japanese (Japan) | Japanese (Japan) vs others | 0.918 | 0.302 | 0.482 | 1.75 | 0.795 |
| Odds of High WMH Burden | South Korean (South Korea) | South Korean (South Korea) vs others | 0.668 | 0.238 | 0.332 | 1.344 | 0.258 |
| Hypertension | Chinese (Hong Kong) | Chinese (Hong Kong) vs others | 2.039 | 1.693 | 0.401 | 10.376 | 0.391 |
| Hypertension | Chinese (Mainland China) | Chinese (Mainland China) vs others | 0.855 | 0.333 | 0.398 | 1.836 | 0.687 |
| Hypertension | Japanese (Japan) | Japanese (Japan) vs others | 0.801 | 0.319 | 0.366 | 1.749 | 0.577 |
| Hypertension | South Korean (South Korea) | South Korean (South Korea) vs others | 0.717 | 0.311 | 0.306 | 1.679 | 0.443 |
| Diabetes | Chinese (Hong Kong) | Chinese (Hong Kong) vs others | 1.364 | 0.457 | 0.707 | 2.631 | 0.354 |
| Diabetes | Chinese (Mainland China) | Chinese (Mainland China) vs others | 1.061 | 0.156 | 0.795 | 1.415 | 0.688 |
| Diabetes | Chinese (Taiwan) | Chinese (Taiwan) vs others | 0.802 | 0.237 | 0.45 | 1.43 | 0.455 |
| Diabetes | Japanese (Japan) | Japanese (Japan) vs others | 0.646 | 0.1 | 0.477 | 0.874 | 0.005 |
| Diabetes | South Korean (South Korea) | South Korean (South Korea) vs others | 1.335 | 0.218 | 0.969 | 1.839 | 0.077 |
| Hyperlipidemia | Chinese (Hong Kong) | Chinese (Hong Kong) vs others | 1.127 | 0.511 | 0.464 | 2.739 | 0.791 |
| Hyperlipidemia | Chinese (Mainland China) | Chinese (Mainland China) vs others | 1.192 | 0.256 | 0.782 | 1.816 | 0.413 |
| Hyperlipidemia | Japanese (Japan) | Japanese (Japan) vs others | 0.889 | 0.206 | 0.564 | 1.402 | 0.614 |
| Hyperlipidemia | South Korean (South Korea) | South Korean (South Korea) vs others | 0.837 | 0.204 | 0.519 | 1.348 | 0.463 |
| High alcohol consumption | Chinese (Mainland China) | Chinese (Mainland China) vs others | 0.638 | 0.195 | 0.351 | 1.162 | 0.142 |
| High alcohol consumption | Japanese (Japan) | Japanese (Japan) vs others | 1.318 | 0.43 | 0.695 | 2.499 | 0.397 |
| High alcohol consumption | South Korean (South Korea) | South Korean (South Korea) vs others | 1.189 | 0.479 | 0.539 | 2.619 | 0.668 |
| Current smoking | Chinese (Mainland China) | Chinese (Mainland China) vs others | 1.556 | 0.33 | 1.027 | 2.357 | 0.037 |
| Current smoking | Chinese (Taiwan) | Chinese (Taiwan) vs others | 0.802 | 0.367 | 0.327 | 1.968 | 0.63 |
| Current smoking | Japanese (Japan) | Japanese (Japan) vs others | 0.946 | 0.203 | 0.62 | 1.441 | 0.794 |
| Current smoking | South Korean (South Korea) | South Korean (South Korea) vs others | 0.848 | 0.203 | 0.531 | 1.355 | 0.49 |
| Age mean | Chinese (Hong Kong) | Chinese (Hong Kong) vs others | 1.076 | 0.025 | 1.028 | 1.127 | 0.002 |
| Age mean | Chinese (Mainland China) | Chinese (Mainland China) vs others | 0.958 | 0.024 | 0.912 | 1.006 | 0.085 |
| Age mean | Chinese (Taiwan) | Chinese (Taiwan) vs others | 1.025 | 0.059 | 0.915 | 1.148 | 0.673 |
| Age mean | Japanese (Japan) | Japanese (Japan) vs others | 0.996 | 0.027 | 0.945 | 1.049 | 0.882 |
| Age mean | South Korean (South Korea) | South Korean (South Korea) vs others | 0.951 | 0.021 | 0.91 | 0.994 | 0.025 |
| Systolic BP mean mm Hg | Chinese (Mainland China) | Chinese (Mainland China) vs others | 1.036 | 0.034 | 0.971 | 1.106 | 0.279 |
| Systolic BP mean mm Hg | Japanese (Japan) | Japanese (Japan) vs others | 0.967 | 0.029 | 0.912 | 1.025 | 0.263 |
| Systolic BP mean mm Hg | South Korean (South Korea) | South Korean (South Korea) vs others | 0.998 | 0.032 | 0.938 | 1.062 | 0.941 |
| Diastolic BP mean mm Hg | Chinese (Mainland China) | Chinese (Mainland China) vs others | 1.135 | 0.226 | 0.768 | 1.676 | 0.526 |
| Diastolic BP mean mm Hg | Japanese (Japan) | Japanese (Japan) vs others | 0.733 | 0.133 | 0.514 | 1.045 | 0.086 |
| Diastolic BP mean mm Hg | South Korean (South Korea) | South Korean (South Korea) vs others | 1.202 | 0.232 | 0.823 | 1.756 | 0.341 |
| Total Cholesterol mean mgDL | Chinese (Mainland China) | Chinese (Mainland China) vs others | 0.885 | 0.028 | 0.831 | 0.942 | <0.0001 |
| Total Cholesterol mean mgDL | Japanese (Japan) | Japanese (Japan) vs others | 1.131 | 0.038 | 1.059 | 1.208 | <0.0001 |
| Total Cholesterol mean mgDL | South Korean (South Korea) | South Korean (South Korea) vs others | 1 | 0.031 | 0.941 | 1.062 | 0.987 |
| HDL cholesterol mean mmolL | Chinese (Mainland China) | Chinese (Mainland China) vs others | 0.789 | 0.069 | 0.666 | 0.936 | 0.007 |
| HDL cholesterol mean mmolL | Japanese (Japan) | Japanese (Japan) vs others | 1.26 | 0.074 | 1.123 | 1.412 | <0.0001 |
| HDL cholesterol mean mmolL | South Korean (South Korea) | South Korean (South Korea) vs others | 1.006 | 0.067 | 0.882 | 1.146 | 0.933 |
| BMI mean kgm2 | Chinese (Mainland China) | Chinese (Mainland China) vs others | 1.028 | 0.008 | 1.011 | 1.044 | <0.0001 |
| BMI mean kgm2 | Chinese (Taiwan) | Chinese (Taiwan) vs others | 1.014 | 0.015 | 0.984 | 1.044 | 0.362 |
| BMI mean kgm2 | Japanese (Japan) | Japanese (Japan) vs others | 0.962 | 0.008 | 0.947 | 0.977 | <0.0001 |
| BMI mean kgm2 | South Korean (South Korea) | South Korean (South Korea) vs others | 0.998 | 0.009 | 0.981 | 1.016 | 0.854 |
| Lacunes | Chinese (Hong Kong) | Chinese (Hong Kong) vs others | 1.095 | 0.333 | 0.604 | 1.987 | 0.765 |
| Lacunes | Chinese (Mainland China) | Chinese (Mainland China) vs others | 1.309 | 0.306 | 0.828 | 2.07 | 0.249 |
| Lacunes | Chinese (Taiwan) | Chinese (Taiwan) vs others | 0.734 | 0.363 | 0.279 | 1.933 | 0.532 |
| Lacunes | Japanese (Japan) | Japanese (Japan) vs others | 1.47 | 0.408 | 0.853 | 2.533 | 0.165 |
| Lacunes | South Korean (South Korea) | South Korean (South Korea) vs others | 0.646 | 0.158 | 0.4 | 1.043 | 0.074 |
| Microinfarcts | Japanese (Japan) | Japanese (Japan) vs others | 0.806 | 0.38 | 0.32 | 2.028 | 0.647 |
| Microinfarcts | South Korean (South Korea) | South Korean (South Korea) vs others | 1.241 | 0.584 | 0.493 | 3.122 | 0.647 |
| CMBs | Chinese (Hong Kong) | Chinese (Hong Kong) vs others | 1.516 | 0.463 | 0.833 | 2.76 | 0.174 |
| CMBs | Chinese (Mainland China) | Chinese (Mainland China) vs others | 1.784 | 0.384 | 1.17 | 2.722 | 0.007 |
| CMBs | Japanese (Japan) | Japanese (Japan) vs others | 0.563 | 0.151 | 0.333 | 0.951 | 0.032 |
| CMBs | South Korean (South Korea) | South Korean (South Korea) vs others | 0.657 | 0.147 | 0.424 | 1.018 | 0.06 |

1. *Estimate*: estimated marginal mean difference (or log-OR).
2. *SE*: standard error.
3. *Lower CI*: lower 95% confidence limit.
4. *Upper CI*: upper 95% confidence limit.
5. *p value*: significance.

# **Supplementary Table 19. Tier 1 Ethnicity CSVD Interaction Model Analyses**

| CSVD_Outcome | Moderator | Interaction_Term (Ethnicity × Moderator) | β | SE | p_value |
| --- | --- | --- | --- | --- | --- |
| Lacunes (Prevalence) | BMI (kg/m²) | Asian × BMI | -3.6918 | 6.0059 | 0.5388 |
| Lacunes (Prevalence) | Systolic BP (mmHg) | Asian × SBP | -0.0363 | 0.1369 | 0.7906 |
| Lacunes (Prevalence) | Diastolic BP (mmHg) | Asian × DBP | 0.0879 | 0.1554 | 0.5716 |
| Lacunes (Prevalence) | Female prevalence | Asian × Female % | 0.0982 | 0.046 | 0.0329 |
| Lacunes (Prevalence) | Diabetes prevalence | Asian × Diabetes % | 0.0965 | 0.0742 | 0.1931 |
| Lacunes (Prevalence) | Hypertension prevalence | Asian × Hypertension % | 0.0387 | 0.0176 | 0.0273 |
| Lacunes (Prevalence) | Current smoking prevalence | Asian × Current smoking % | 0.026 | 0.0201 | 0.1952 |
| WMH Volume | Systolic BP (mmHg) | Asian × SBP | 0.096 | 0.076 | 0.2066 |
| WMH Volume | Diastolic BP (mmHg) | Asian × DBP | 0.0294 | 0.0837 | 0.725 |
| WMH Volume | Female prevalence | Asian × Female % | 0.0232 | 0.0367 | 0.5285 |
| WMH Volume | Female prevalence | Black × Female % | -0.0012 | 0.0304 | 0.9672 |
| WMH Volume | Female prevalence | Hispanic × Female % | 0.0139 | 0.0405 | 0.7307 |
| Odds of high WMH burden | Female prevalence | Female % | -0.0121 | 0.0515 | 0.814 |
| Odds of high WMH burden | Female prevalence | Hispanic × Female % | 0.0328 | 0.0339 | 0.3329 |
| Odds of high WMH burden | Diabetes prevalence | Asian × Diabetes % | -0.0833 | 0.0597 | 0.1626 |
| CMBs (Prevalence) | BMI (kg/m²) | Asian × BMI (kg/m²) | 0.0416 | 0.749 | 0.9557 |
| CMBs (Prevalence) | Systolic BP (mmHg) | Asian × Systolic BP (mmHg) | -0.0026 | 0.0624 | 0.9674 |
| CMBs (Prevalence) | Diastolic BP (mmHg) | Asian × Diastolic BP (mmHg) | 0.1525 | 0.0919 | 0.097 |
| CMBs (Prevalence) | Female prevalence | Hispanic × Female % | -0.0711 | 0.0578 | 0.2187 |
| CMBs (Prevalence) | Female prevalence | Asian × Female % | -0.0221 | 0.0228 | 0.3309 |
| CMBs (Prevalence) | Diabetes prevalence | Hispanic × Diabetes prevalence | 0.0094 | 0.0587 | 0.8722 |
| CMBs (Prevalence) | Diabetes prevalence | Asian × Diabetes prevalence | 0.0007 | 0.0786 | 0.9931 |
| CMBs (Prevalence) | Hypertension prevalence | Hispanic × Hypertension prevalence | -0.0006 | 0.0158 | 0.9685 |
| CMBs (Prevalence) | Hypertension prevalence | Asian × Hypertension prevalence | -0.028 | 0.0251 | 0.2639 |
| CMBs (Prevalence) | Current smoking prevalence | Hispanic × Current smoking prevalence | 0.0319 | 0.0225 | 0.1567 |
| CMBs (Prevalence) | Current smoking prevalence | Asian × Current smoking prevalence | 0.0218 | 0.0148 | 0.1405 |
| Microinfarcts (Prevalence) | Female prevalence | Asian × Female % | 0.7591 | 0.4761 | 0.1109 |
| Microinfarcts (Prevalence) | Diabetes prevalence | Asian × Diabetes prevalence | -0.0487 | 0.3457 | 0.8881 |
| Microinfarcts (Prevalence) | Current smoking prevalence | Asian × Current smoking prevalence | 0.1209 | 0.1114 | 0.2779 |

1. *β*: regression coefficient.
2. *SE*: standard error.
3. *p-value*: significance.

# **Supplementary Table 20. Tier 2 Asian CSVD Interaction Model Analyses**

| CSVD Outcome | Model or Moderator | Effect (Ethnicity×Moderator) | β | SE | p value |
| --- | --- | --- | --- | --- | --- |
| Odds of high WMH burden | BMI (kg/m²) | Mainland China×BMI | -1.0687 | 1.7756 | 0.5472 |
| Odds of high WMH burden | BMI (kg/m²) | South Korea×BMI | 0.9372 | 1.8515 | 0.6127 |
| Odds of high WMH burden | Systolic BP (mmHg) | Mainland China×SBP | -0.0247 | 0.05 | 0.6215 |
| Odds of high WMH burden | Systolic BP (mmHg) | South Korea×SBP | 0.2077 | 0.0959 | 0.0304 |
| Odds of high WMH burden | Diastolic BP (mmHg) | Mainland China×DBP | 0.0805 | 0.1544 | 0.6023 |
| Odds of high WMH burden | Diastolic BP (mmHg) | South Korea×DBP | 0.2235 | 0.1893 | 0.2378 |
| Odds of high WMH burden | Female prevalence | Mainland China×Female | -0.0131 | 0.0347 | 0.7059 |
| Odds of high WMH burden | Female prevalence | South Korea×Female | 0.0456 | 0.0339 | 0.1789 |
| Odds of high WMH burden | Diabetes prevalence | Mainland China×Diabetes | 0.2023 | 0.0921 | 0.028 |
| Odds of high WMH burden | Diabetes prevalence | South Korea×Diabetes | 0.1528 | 0.0996 | 0.1248 |
| Odds of high WMH burden | Hyperlipidemia prevalence | Mainland China×Hyperlipidemia | 0.0334 | 0.0281 | 0.2338 |
| Odds of high WMH burden | Hyperlipidemia prevalence | South Korea×Hyperlipidemia | 0.0449 | 0.0326 | 0.1684 |
| Odds of high WMH burden | Hypertension prevalence | Mainland China×Hypertension | -0.0061 | 0.0193 | 0.7527 |
| Odds of high WMH burden | Hypertension prevalence | South Korea×Hypertension | 0.0252 | 0.0202 | 0.2124 |
| WMH Volume | Female prevalence | South Korea×Female | -0.2236 | 1.2171 | 0.8543 |
| WMH Volume | Diabetes prevalence | South Korea×Diabetes | -0.801 | 0.3313 | 0.0156 |
| Lacunes | BMI (kg/m²) | Mainland China×BMI | 1.0238 | 0.6645 | 0.1234 |
| Lacunes | BMI (kg/m²) | South Korea×BMI | -0.2096 | 1.0753 | 0.8454 |
| Lacunes | Systolic BP (mmHg) | Mainland China×SBP | -0.0511 | 0.0472 | 0.2788 |
| Lacunes | Systolic BP (mmHg) | South Korea×SBP | 0.0161 | 0.1109 | 0.8845 |
| Lacunes | Diastolic BP (mmHg) | Mainland China×DBP | 0.2462 | 0.2654 | 0.3536 |
| Lacunes | Diastolic BP (mmHg) | South Korea×DBP | -0.0125 | 0.2694 | 0.963 |
| Lacunes | Female prevalence | Japan×Female | 0.1646 | 0.0919 | 0.0733 |
| Lacunes | Female prevalence | Mainland China×Female | 0.137 | 0.0902 | 0.1289 |
| Lacunes | Female prevalence | South Korea×Female | 0.1557 | 0.1038 | 0.1336 |
| Lacunes | Diabetes prevalence | Mainland China×Diabetes | 0.2023 | 0.0921 | 0.028 |
| Lacunes | Diabetes prevalence | South Korea×Diabetes | 0.1528 | 0.0996 | 0.1248 |
| Lacunes | Diabetes prevalence | Taiwan×Diabetes | -0.2373 | 0.4446 | 0.5936 |
| CMBs | Female prevalence | Mainland China×Female | 0.1625 | 0.0897 | 0.0678 |
| CMBs | Female prevalence | South Korea×Female | 0.1152 | 0.0935 | 0.2281 |
| CMBs | Female prevalence | Japan×Female | 0.1546 | 0.0951 | 0.1013 |
| CMBs | Diabetes prevalence | Mainland China×Diabetes | 0.1871 | 0.0872 | 0.0335 |
| CMBs | Diabetes prevalence | South Korea×Diabetes | 0.0959 | 0.092 | 0.2937 |
| CMBs | Diabetes prevalence | Japan×Diabetes | 0.1034 | 0.0835 | 0.2152 |
| Microinfarcts | Diabetes prevalence | South Korea×Diabetes | 0.2211 | 0.1357 | 0.1078 |
| Microinfarcts | Diabetes prevalence | Mainland China×Diabetes | 0.1189 | 0.1423 | 0.4051 |
| Microinfarcts | Female prevalence | South Korea×Female | 0.1734 | 0.1217 | 0.1559 |
| Microinfarcts | Female prevalence | Mainland China×Female | 0.1208 | 0.1284 | 0.3452 |

1. *β*: regression coefficient.
2. *SE*: standard error.
3. *p-value*: significance

# **Figure 8 Tier 1 Interaction analyses Plot**


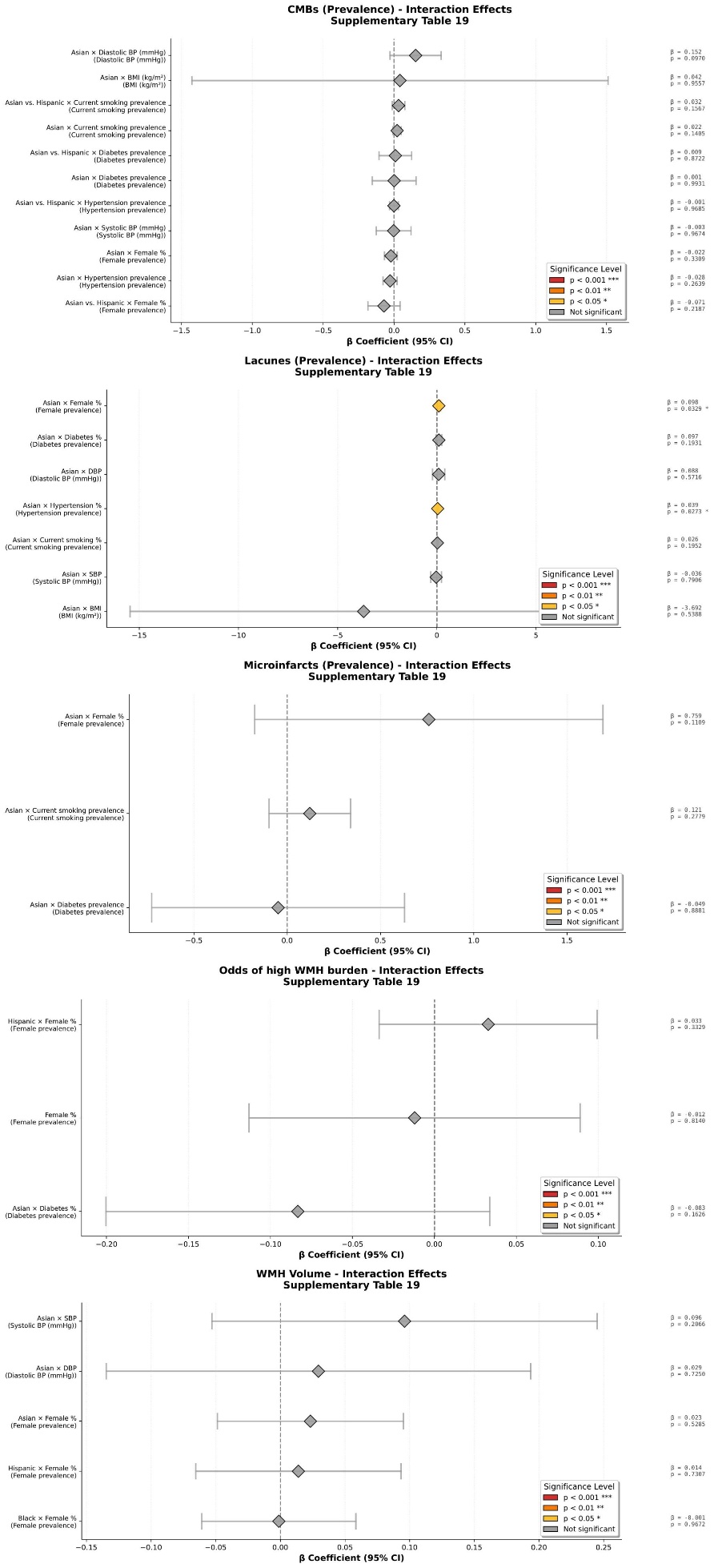


# **Figure 9 Tier 2 Interaction analyses Plot**


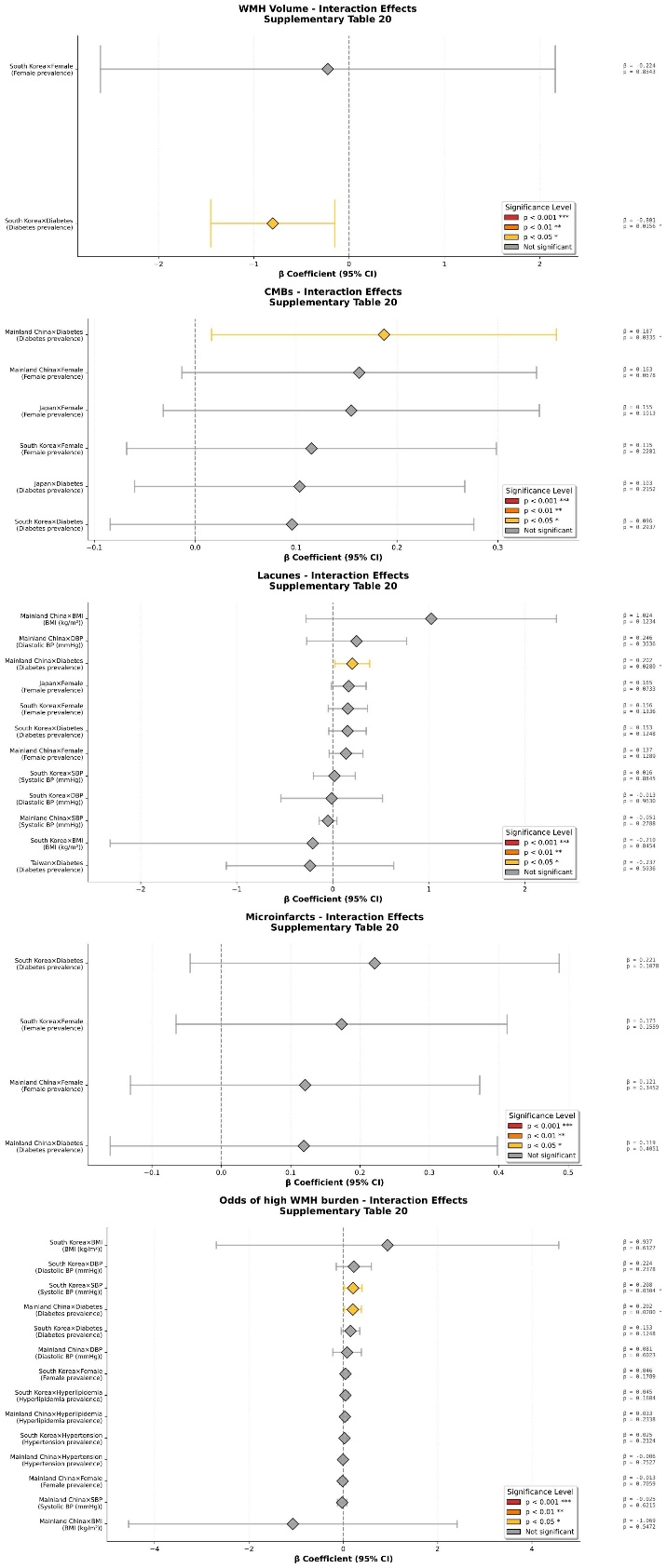

Supplement: Supplementary file 1 — Supporting information [file ALZ-22-e70976-s002.docx]
